# Japanese Classification of Esophageal Cancer, 12th Edition: Part II

**DOI:** 10.1007/s10388-024-01048-w

**Published:** 2024-03-21

**Authors:** Yuichiro Doki, Koji Tanaka, Hiroshi Kawachi, Yasuhiro Shirakawa, Yuko Kitagawa, Yasushi Toh, Takushi Yasuda, Masayuki Watanabe, Takashi Kamei, Tsuneo Oyama, Yasuyuki Seto, Kentaro Murakami, Tomio Arai, Manabu Muto, Shinji Mine

**Affiliations:** 1 Department of Gastroenterological Surgery, Graduate School of Medicine, Osaka University, Osaka, Japan; 2 Division of Pathology, Cancer Institute, Japanese Foundation for Cancer Research, Tokyo, Japan; 3 Department of Surgery, Hiroshima City Hiroshima Citizens Hospital, Hiroshima, Japan; 4 Department of Surgery, Keio University School of Medicine, Tokyo, Japan; 5 Department of Gastroenterological Surgery, National Hospital Organization Kyushu Cancer Center, Fukuoka, Japan; 6 Department of Surgery, Faculty of Medicine, Kindai University, Osaka, Japan; 7 Department of Gastroenterological Surgery, Cancer Institute Hospital of Japanese Foundation for Cancer Research, Tokyo, Japan; 8 Department of Surgery, Tohoku University Graduate School of Medicine, Miyagi, Japan; 9 Department of Endoscopy, Saku Central Hospital Advanced Care Center, Nagano, Japan; 10 Department of Gastrointestinal Surgery, Graduate School of Medicine, The University of Tokyo, Tokyo, Japan; 11 Department of Frontier Surgery, Graduate School of Medicine, Chiba University, Chiba, Japan; 12 Department of Pathology, Tokyo Metropolitan Institute for Geriatrics and Gerontology, Tokyo, Japan; 13 Department of Clinical Oncology, Kyoto University Hospital, Kyoto, Japan; 14 Department of Esophageal and Gastroenterological Surgery, Juntendo University Hospital, 3-1-3, Hongo, Bunkyo-Ku, Tokyo, Japan

**Keywords:** Esophageal cancer, Japanese classification, Endoscopic treatment, Surgery, Chemotherapy, Radiotherapy

## Abstract

This is the second half of English edition of Japanese Classification of Esophageal Cancer, 12th Edition that was published by the Japan Esophageal Society in 2022.

**Contents**

- Abbreviations
- Terminology of the lymph node
- General rules
- Response evaluation criteria in radiotherapy and chemotherapy for esophageal cancer
  - Introduction
- References

- General rules
- 7 Pathological findings
  - 7.1 Description of pathological findings
    - 7.1.1 Histological classification
      - 7.1.1.1 Benign epithelial neoplasia
      - 7.1.1.2 Intraepithelial neoplasia
      - 7.1.1.3 Malignant epithelial neoplasm
      - 7.1.1.4 Non-epithelial tumor
      - 7.1.1.5 Lymphoid tumor
      - 7.1.1.6 Other malignant tumors
      - 7.1.1.7 Tumor-like lesions
    - 7.1.2 Other findings to be described
    - 7.1.3 Report of pathological findings
- 8 Esophagogastric junction and Barrett's esophagus
  - 8.1 Definition of the esophagogastric junction (EGJ)
    - 8.1.1 Endoscopy
    - 8.1.2 X-ray: Upper gastrointestinal imaging
    - 8.1.3 Pathological study
  - 8.2 Barrett’s esophagus
    - 8.2.1 Definition of Barrett’s esophagus
    - 8.2.2 Histological types of Barrett’s mucosa
  - 8.3 Adenocarcinoma in Barrett’s esophagus
  - 8.4 Esophagogastric junction cancer (EGJ cancer)
    - 8.4.1 Definition
    - 8.4.2 Staging
    - 8.4.3 Tumor location
    - 8.4.4 Esophageal invasion (EI)
    - 8.4.5 Gastric invasion (GI)
    - 8.4.6 Barrett’s esophagus (BE)
    - 8.4.7 Hiatus hernia (HH)
- 9 Treatment
  - 9.1 Endoscopic treatment
    - 9.1.1 Endoscopic resection (ER)
      - 9.1.1.1 Endoscopic mucosal resection (EMR)
      - 9.1.1.2 Endoscopic submucosal dissection (ESD)
    - 9.1.2 Other endoscopic treatment
      - 9.1.2.1 Argon plasma coagulation (APC)
      - 9.1.2.2 Laser therapy (Laser)
      - 9.1.2.3 Photodynamic therapy (PDT)
      - 9.1.2.4. Microwave coagulation therapy (MCT)
      - 9.1.2.5 Others
  - 9.2 Surgical treatment
    - 9.2.1 Resection and reconstruction procedure
      - 9.2.1.1 Staged operation
      - 9.2.1.2 Approaches for tumor resection
      - 9.2.1.3 Positions
      - 9.2.1.4 Extent of resection
      - 9.2.1.5 Combined resection
      - 9.2.1.6 Reconstruction
        - 9.2.1.6.1 Routes
        - 9.2.1.6.2 Sites of anastomosis
        - 9.2.1.6.3 Organs for substitution
    - 9.2.2 Conservative/palliative procedure
      - 9.2.2.1 Stoma
      - 9.2.2.2 Bypass
      - 9.2.2.3 Exploratory thoracotomy, exploratory laparotomy, staging thoracoscopy, staging laparoscopy
  - 9.3 Stenting
    - 9.3.1 Esophageal stent
    - 9.3.2 Tracheobronchial stent
    - 9.3.3 Aortic stent
  - 9.4 Balloon dilatation
  - 9.5 Common issues for radiotherapy and chemotherapy
    - 9.5.1 Aim of treatment
  - 9.6 Radiotherapy (RT)
    - 9.6.1 Clinical target volume (CTV)
    - 9.6.2 Methods of radiotherapy
    - 9.6.3 External beam radiotherapy
      - 9.6.3.1 Planning methods
      - 9.6.3.2 Field setting
      - 9.6.3.3 Reference points
      - 9.6.3.4 Dose calculation
      - 9.6.3.5 Dose fractionation of external beam radiotherapy
    - 9.6.4 Intraluminal irradiation
      - 9.6.4.1 Reference points
      - 9.6.4.2 Dose fractionation of intraluminal irradiation
    - 9.6.5 Completion of treatment
    - 9.6.6 Chemoradiation therapy (CRT)
  - 9.7 Chemotherapy (CT), Immune-oncology drug (IO)
    - 9.7.1 Aim of treatment
    - 9.7.2 Agent
    - 9.7.3 Administration route
    - 9.7.4 Administration procedure
    - 9.7.5 Administration dose
    - 9.7.6 Administration schedule
    - 9.7.7 Duration of administration
    - 9.7.8 Total administration dose
    - 9.7.9 Adverse events
  - 9.8 Multi-modality treatment
    - 9.8.1 Surgery in multi-modality treatment
    - 9.8.2 Endoscopic treatment in multi-modality treatment
  - 9.9 Hyperthermia (HT)
- 10 Treatment results
  - 10.1 Long-term outcome
    - 10.1.1 Alive or dead
    - 10.1.2 Recurrence

**Response evaluation criteria in radiotherapy and chemotherapy for esophageal cancer**

- 11. Definitions
  - 11.1. Classification of tumor lesions and lymph nodes
    - 11.1.1. Measurable lesions
    - 11.1.2. Non-measurable lesions
      - 11.1.3. Target lesions
      - 11.1.4. Non-target lesions
- 12. Response evaluation criteria for target lesions
  - 12.1. Complete response (CR)
  - 12.2. Partial response (PR)
  - 12.3. Progressive disease (PD)
  - 12.4. Stable disease (SD)
- 13. Response evaluation criteria for non-target lesions
  - 13.1. Complete response (CR)
  - 13.2. Non-CR/non-PD
  - 13.3. Progressive disease (PD)
- 14. Response evaluation criteria of CT/PET-CT for primary tumors
  - 14.1. Response evaluation criteria of CT
    - 14.1.1. Evaluation using short diameter of primary tumor
    - 14.1.2. Evaluation using esophageal cross-section area
  - 14.2. Response evaluation criteria of PET-CT
- 15. Response evaluation criteria for primary lesion using endoscopy
  - 15.1. Complete response of primary lesion (primary lesion CR)
  - 15.2. Non-CR/non-PD of primary lesion (primary lesion non-CR/non-PD)
    - 15.2.1. Remarkable response of primary lesion (primary lesion RR)
  - 15.3. Progressive disease of primary lesion (primary lesion PD)
  - 15.4. Local recurrence of primary lesion during follow-up (primary lesion LR)
- 16. Overall response
- 17. Best overall response and confirmation
- 18. Criteria for histological response of chemotherapy and/or radiotherapy

**References**

- 19. Naming, number, range and boundary of regional lymph node of esophageal cancer
  - 19.1 Cervical lymph nodes
  - 19.2 Thoracic lymph nodes
  - 19.3 Abdominal lymph nodes
- 20. Diagnostic criteria of CT and PET-CT for lymph node metastasis
- 21. Usefulness and validity of response criteria according to measurement method
  - 21.1. Correlation between the type of primary tumor measurement method and prognosis
  - 21.2. Correlation between the reduction rate in SUVmax and prognosis in PET-CT
- 22. Response evaluation criteria for primary lesion using endoscopy
  - 22.1. Endoscopic findings of CR cases
  - 22.2. Endoscopic findings of non-CR/non-PD cases
  - 22.3. Endoscopic findings of non-CR/non-PD (RR) cases
  - 22.4. Endoscopic findings of a PD case
  - 22.5. Endoscopic findings of LR cases
- 23. Histological criteria for the effects of chemotherapy and/or radiation
- 24. Survival curve according to stage

**General rules**

## 7. Pathological findings

### 7.1 Description of pathological findings

#### 7.1.1 Histological classification

##### 7.1.1.1 Benign epithelial neoplasia

1. Squamous cell papilloma

Squamous cell papilloma is considered not a true neoplasm but a reactive hyperplasia. It contains fibrovascular stroma and shows papillary growth of the squamous epithelium without atypia. This lesion occasionally has clear cytoplasm with vacuoles (koilocytosis), suggesting viral infection. It is important to differentiate this lesion from a verrucous carcinoma in the biopsy specimens.

2. Adenoma ^*Notes 1, 2*^

Adenoma rarely arises from a squamous-lined esophagus. A few reports have described esophageal adenomas arising from the proper esophageal gland or duct.

*Note 1:* Neoplasm arising from Barrett’s mucosa is excluded. The classification of neoplasm in Barrett’s esophagus should be recorded according to the Japanese Classification of Gastric Carcinoma [1].

*Note 2:* There are very few reports of benign tumor in Barrett’s esophagus. However, there is no consensus regarding the diagnostic criteria or method of description of adenoma in Barrett’s esophagus.

##### 7.1.1.2 Intraepithelial neoplasia ^*Note 1–3*^

**Fig. 1:**
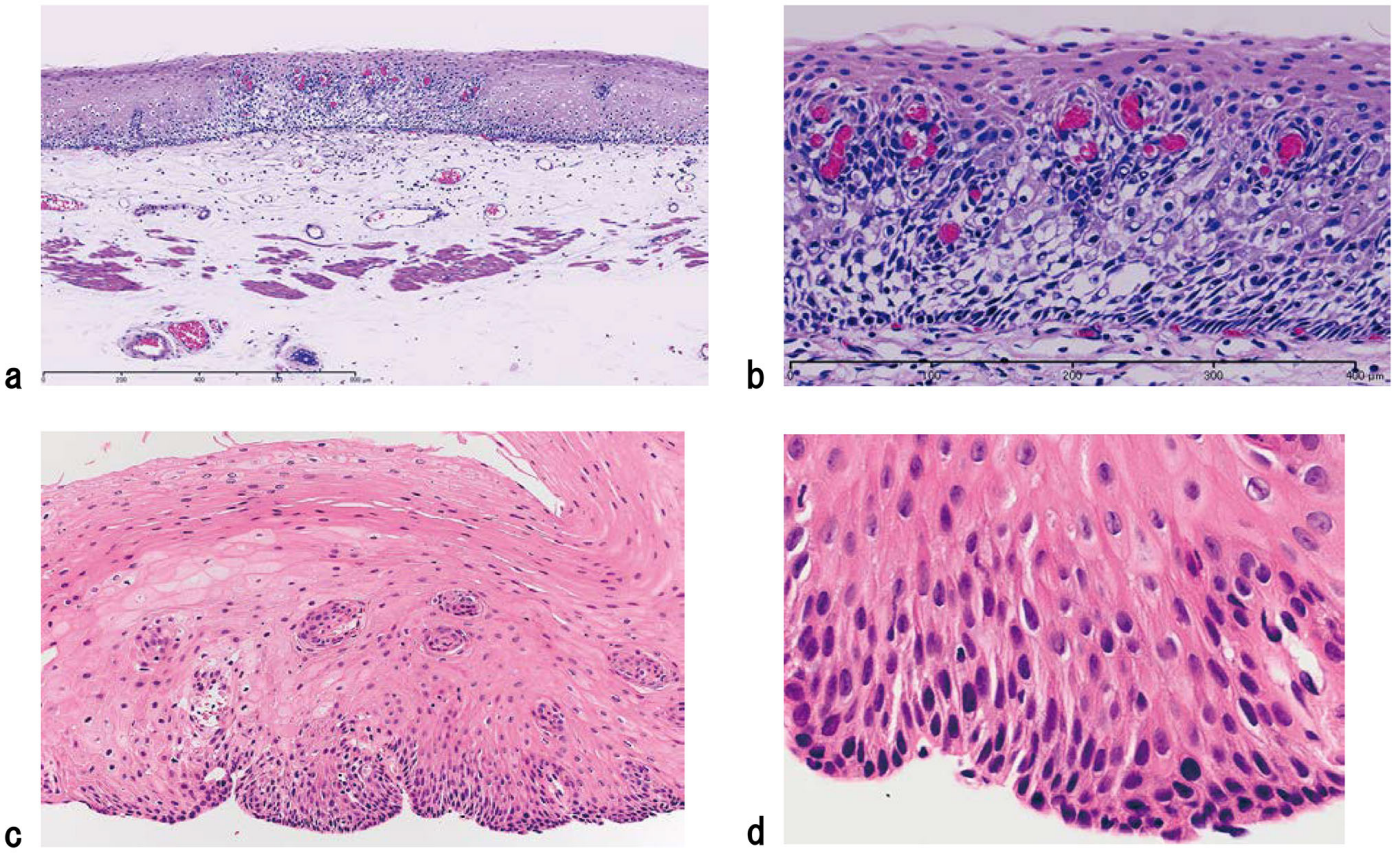
Squamous intraepithelial neoplasia. **a** and **b** Resected specimen of squamous intraepithelial neoplasia. The lesion is visible as an iodine-unstained area measuring 3 mm in size. **a** Middle magnification of squamous intraepithelial neoplasia. Histologically, the lesion is well demarcated. **b** High magnification. The tumor shows mild nuclear atypia with low cellular density and a regular arrangement of the basal layer. **c** Biopsy specimen of the squamous intraepithelial neoplasia. A brownish area appears on endoscopy, which tends to differentiate to a superficial layer with slightly high cellular density. **d** High magnification around the basal side area of Fig. 1c. Tumor cells show slight nuclear enlargement and hyperchromasia (c and d: reprint with permission from the Atlas for differential diagnosis of tumor pathology and esophageal cancer, 2.^nd^ edition [4])

1. Squamous intraepithelial neoplasia (carcinoma in situ is excluded) (Fig. 1)

Intraepithelial neoplasia is defined as an intraepithelial lesion showing structural and cytological abnormalities indicative of a neoplasm; however, it does not include carcinoma in situ.

Explanations

1. Intraepithelial neoplasia that exhibits structural and cytological abnormalities, but does not include carcinoma, is defined as squamous intraepithelial neoplasia (SIN). So far, non-invasive neoplasia of the esophageal squamous epithelium has been known as squamous dysplasia or intraepithelial neoplasia. According to the WHO classification 5th edition (2019) [2], squamous dysplasia was recommended although intraepithelial neoplasia was used until the 4th edition of WHO classification [3]. This Japanese Classification of Esophageal Cancer 12th edition does not use “squamous dysplasia” but SIN as well as the policy of Japanese Classification of Esophageal Cancer 11th edition.
2. According to WHO 5th edition [2], squamous dysplasia is classified as high grade or low grade. In Japan, however, high-grade squamous dysplasia of WHO classification is generally diagnosed as a carcinoma in situ. Therefore, in the Japanese classification, “high-grade squamous dysplasia” or “high-grade intraepithelial neoplasia” is not used and only low-grade squamous dysplasia in WHO classification 5th edition [2] is defined as SIN in this classification, that is, no grading system is used for SIN in this classification. When we diagnose this lesion using both the WHO and Japanese classifications, “grade” status only should be added in the WHO classification (e.g., squamous intraepithelial neoplasia/low-grade squamous dysplasia in WHO classification).
3. Typical histological findings of SIN are as follows: neoplastic cells in the basal side of the squamous epithelium have slightly atypical round or oval nuclei, and show mild-to-moderate cellular density; they transition into flattened cells with flat nuclei to the surface, and their cellular density declines.

*Note 1:* Differential diagnosis of SIN includes lesions with reactive epithelial atypia due to inflammation and regeneration. For instance, a regenerative squamous epithelium with reflux esophagitis sometimes exhibits atypical basal or parabasal cells; however, these lesions should not be diagnosed as intraepithelial neoplasia. When the discrimination of neoplasia from reactive change is difficult, we recommend using “atypical squamous epithelium, indefinite for neoplasia.” For such case, follow-up or re-biopsy is recommended according to the endoscopic findings and clinical course.

*Note 2:* Although intraepithelial neoplasia of the esophagus includes not only squamous epithelial origin but also columnar epithelial origin related to Barrett’s esophagus, this chapter focuses only on the squamous epithelium.

*Note 3:* Most SINs are endoscopically or macroscopically recognized as “small unstained or tan-stained areas”. These lesions can be solitary or multiple (mottled).

##### 7.1.1.3 Malignant epithelial neoplasm

Histological subtyping must be performed based on the predominant histological features of the tumor.

**Fig. 2:**
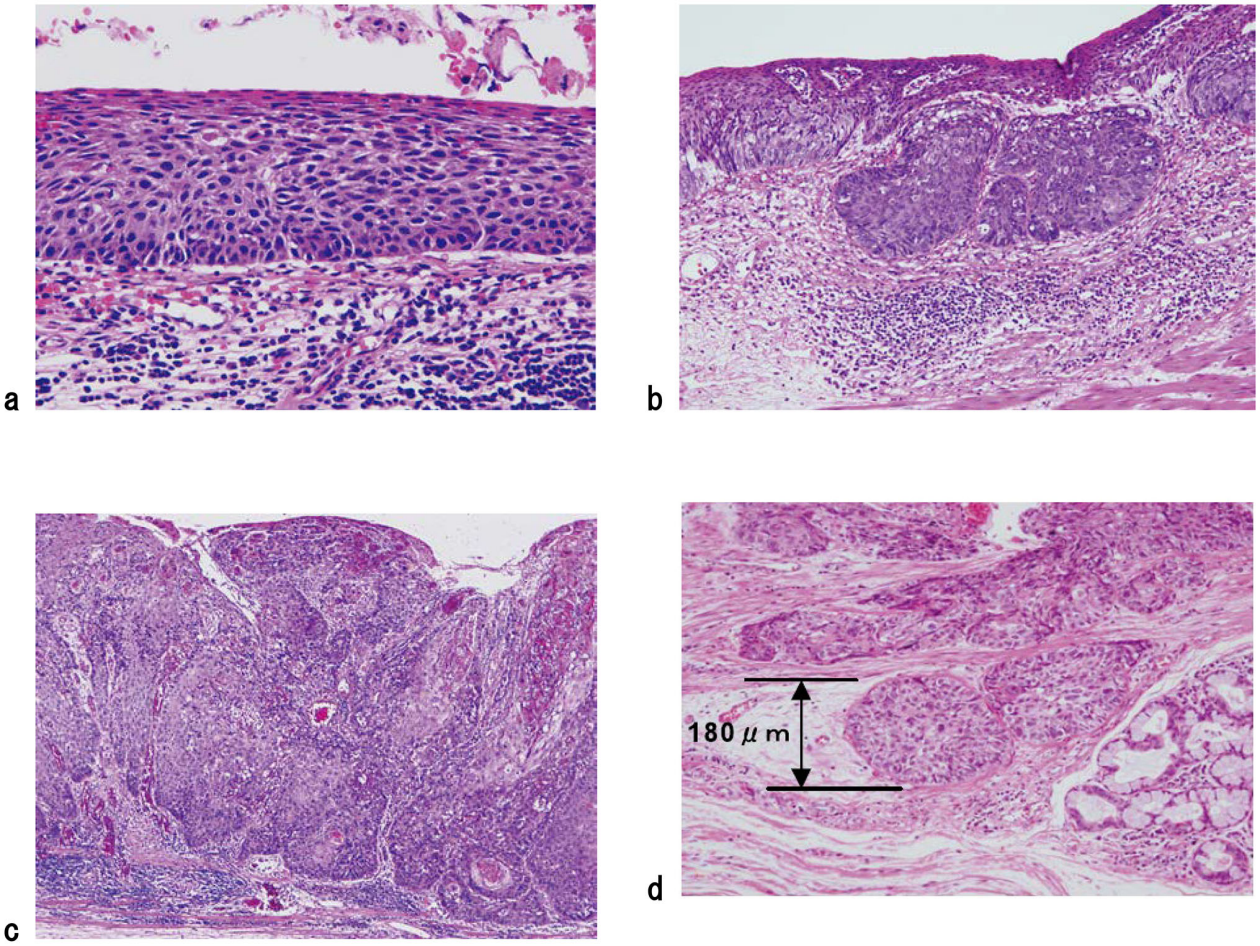
Squamous cell carcinoma (pT1). **a** T1a-EP (Tis): Carcinoma in situ. **b** T1a-LPM: Tumor invades lamina propria mucosa (LPM). **c** T1a-MM: Tumor invades muscular mucosa (MM). **d** T1b-SM1: Tumor invades the upper third of the submucosal layer (the invasion distance from the muscular mucosa was 180 μm, corresponding to SM1 for EMR/ESD)

**Fig. 3:**
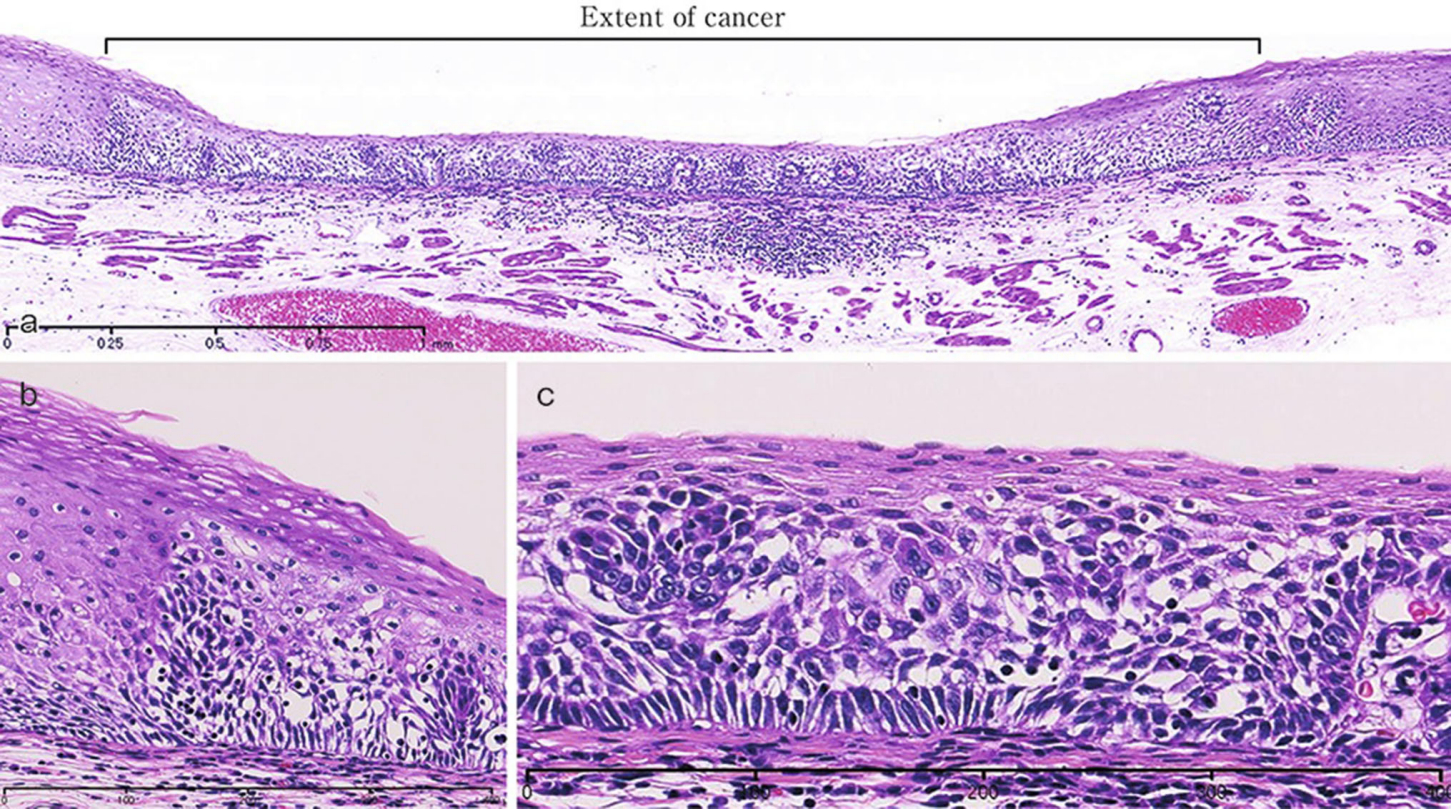
Squamous cell carcinoma (pT1a-EP). **a** The low-power view of squamous cell carcinoma shows a clear border between the carcinoma and the non-neoplastic epithelium. **b** Histology of the border between the squamous cell carcinoma and non-neoplastic squamous epithelium, wherein the cancer tissues exhibit high cellular density with a loss of the basal layer. **c** Histology of the central portion of cancer tissue shows cancer cells have proliferated throughout the entire epithelial layer but does not invade the lamina propria mucosa

**Fig. 4:**
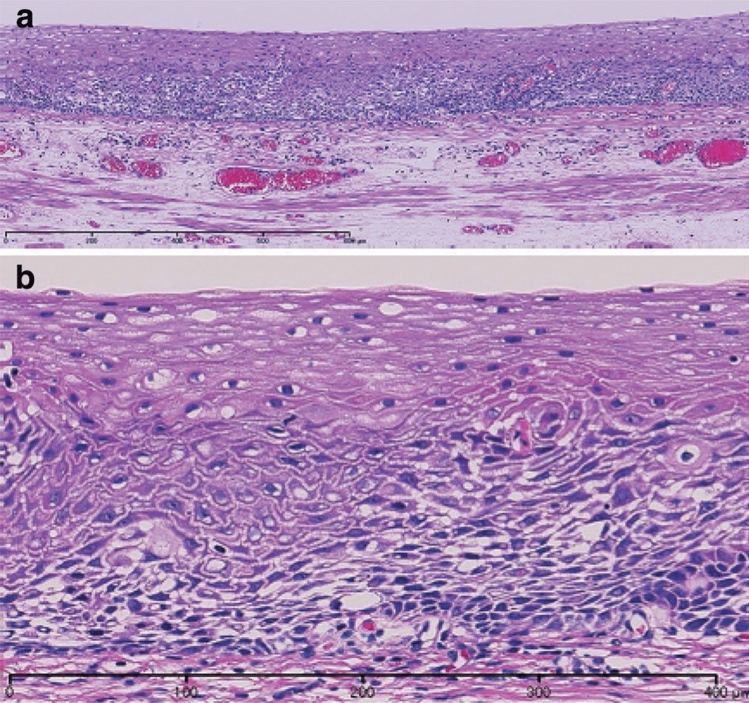
Basal layer-type squamous cell carcinoma (pT1a-EP). **a** Cancer cells are present within the lower half of the epithelium, whereas squamous epithelial cells with minimal atypia are present in the upper half. **b** The high-power view of the shows that non-neoplastic basal cells have disappeared, and that the lower half of the epithelium has been replaced by cancer cells with nuclear atypia and a higher cellular density. In addition, squamous epithelial cells in the upper half have small-sized nuclei and show lower cellular density

**Fig. 5:**
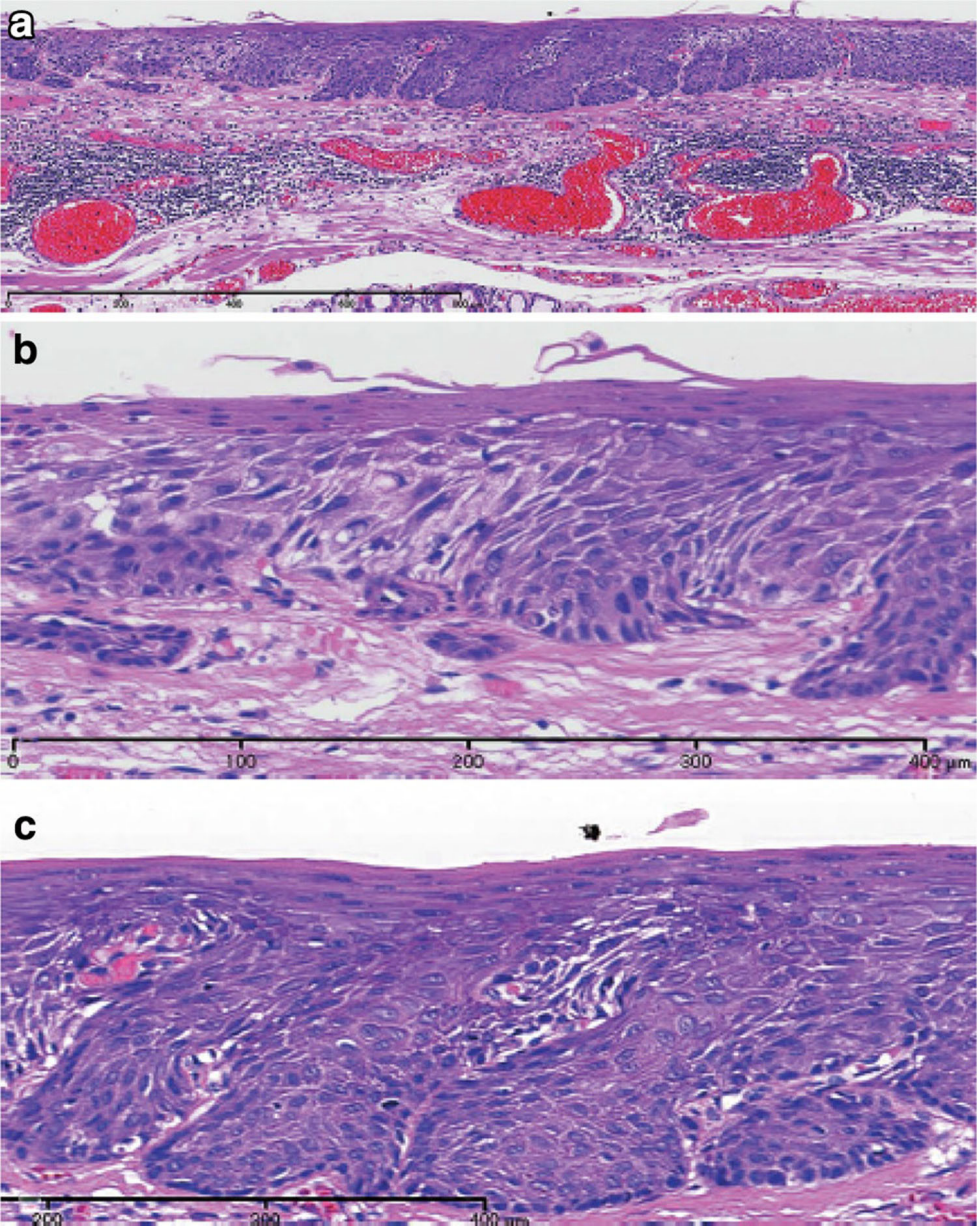
Squamous cell carcinoma invading the lamina propria mucosa (pT1a-LPM). **a** Squamous cell carcinoma shows mild thickening of the epithelial layer and irregular downward growth to the lamina propria mucosa. **b** Droplet infiltration is observed in the lamina propria mucosa. **c** The expansive downward growth of squamous cell carcinoma

**Fig. 6:**
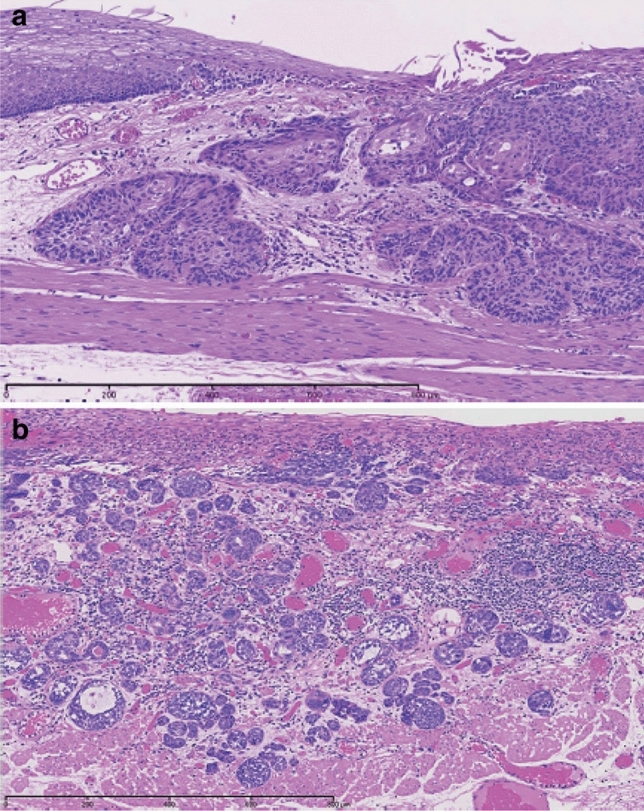
Squamous cell carcinoma with invasion into the muscularis mucosa (pT1a-MM). **a** Cancer cells reach the upper end of the muscularis mucosa and partly invade it. Both are classified as pT1a-MM. **b** Cancer cells invade the muscularis mucosa but not beyond

**Fig. 7:**
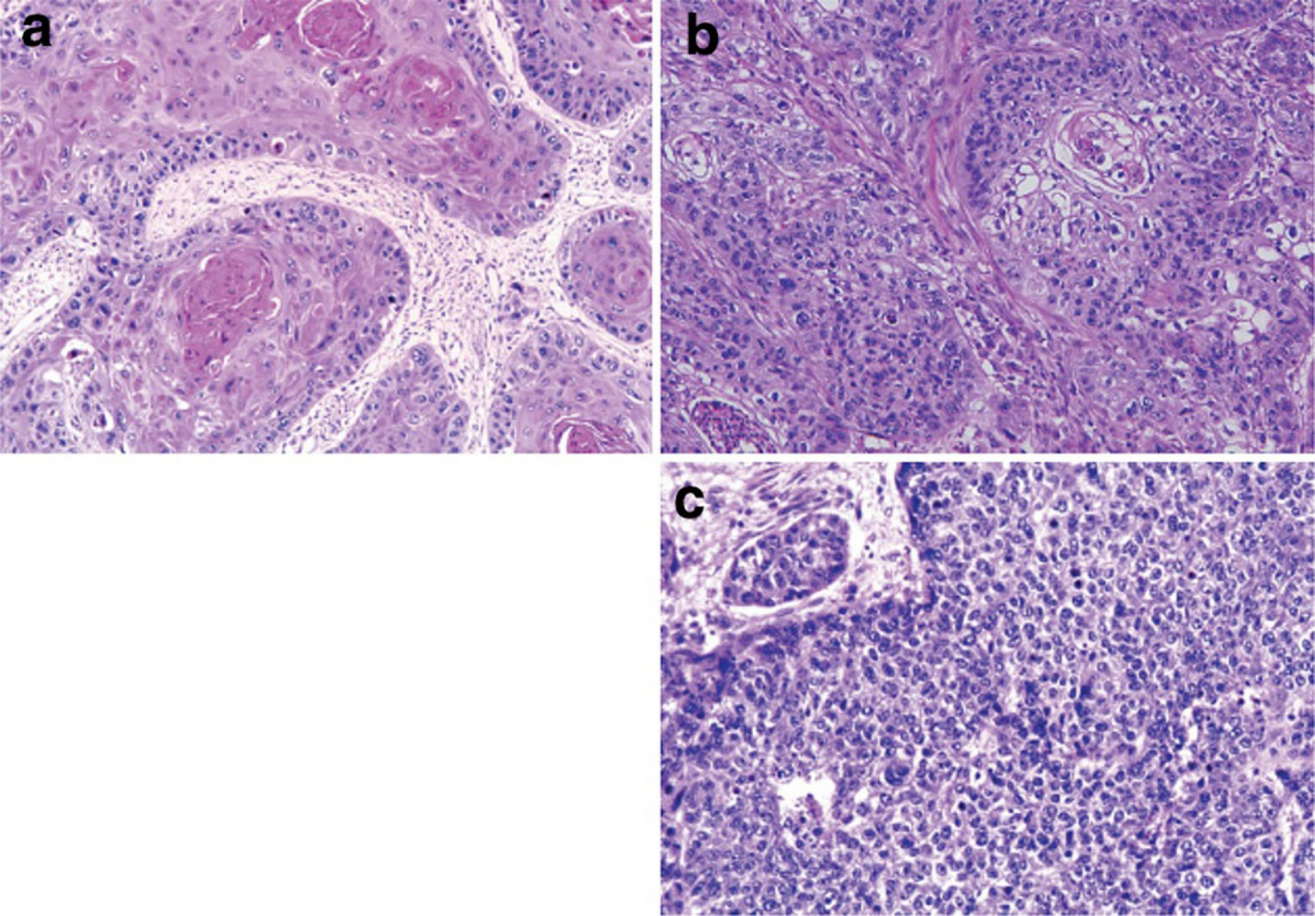
Squamous cell carcinoma. **a** Well-differentiated squamous cell carcinoma: Cancer pearls with marked keratinization are observed. **b** Moderately differentiated squamous cell carcinoma: Sheet-like arrangement of tumor cells with slight keratinization is observed. **c** Poorly differentiated squamous cell carcinoma: Keratinization is not observed, although tumor cells show sheet-like arrangement

1. Squamous cell carcinoma (Figs. 2, 3, 4, 5,6, 7)

- Well differentiated
- Moderately differentiated
- Poorly differentiated.

In situ squamous cell carcinoma is equivalent to squamous cell carcinoma with pT1a-EP. Invasive squamous cell carcinoma frequently forms solid nests of tumor cells that differentiate into stratified squamous epithelial cells. Keratinization, stratification, and intercellular bridges are frequently observed. A few tubules and a small amount of epithelial mucus may exist in the tumor; these features should be noted, if present.

**Fig. 8:**
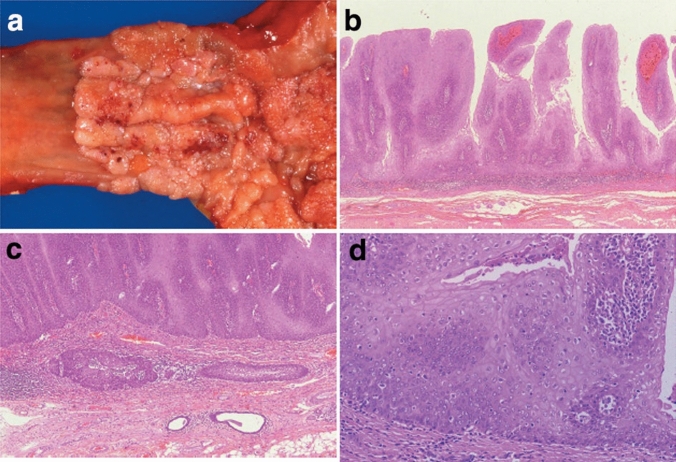
Verrucous squamous cell carcinoma. **a** Gross features of the verrucous squamous cell carcinomas show that the tumor involves the cervical esophagus and hypopharynx, and has a granular surface. **b** Low-power view of verrucous squamous cell carcinoma shows prominent papillary growth, and the basal side is generally flat. **c** Verrucous squamous cell carcinoma with invasion into the lamina propria mucosa. **d** High-power view of the verrucous squamous cell carcinoma. The tumor shows a high cellular density and an irregular arrangement at the basal site, whereas differentiation is apparent toward the surface

*Note:* Squamous cell carcinoma in situ can be diagnosed based on structural and cytological atypia. Structural atypia includes cellular density (cellularity), cell differentiation, and loss of polarity in the basal layer. Cytological atypia includes variations in nuclear size and shape, hyperchromasia, loss of polarity, prominent nucleoli, increased nuclear/cytoplasmic ratios, and mitosis. It is not necessary to describe the degree of differentiation in squamous cell carcinoma in situ. Invasive squamous cell carcinoma is sub-classified into three groups based on the degree of keratinization and stratification: well-differentiated, moderately differentiated, and poorly differentiated. Verrucous squamous cell carcinoma, a type of very well-differentiated squamous cell carcinoma with papillary growth, is known but extremely rare (Fig. 8). Poorly differentiated squamous cell carcinoma can be composed of spindle-shaped cells and may resemble sarcoma; such lesions are classified as spindle cell carcinoma or spindle cell variants of squamous cell carcinoma.

**Fig. 9:**
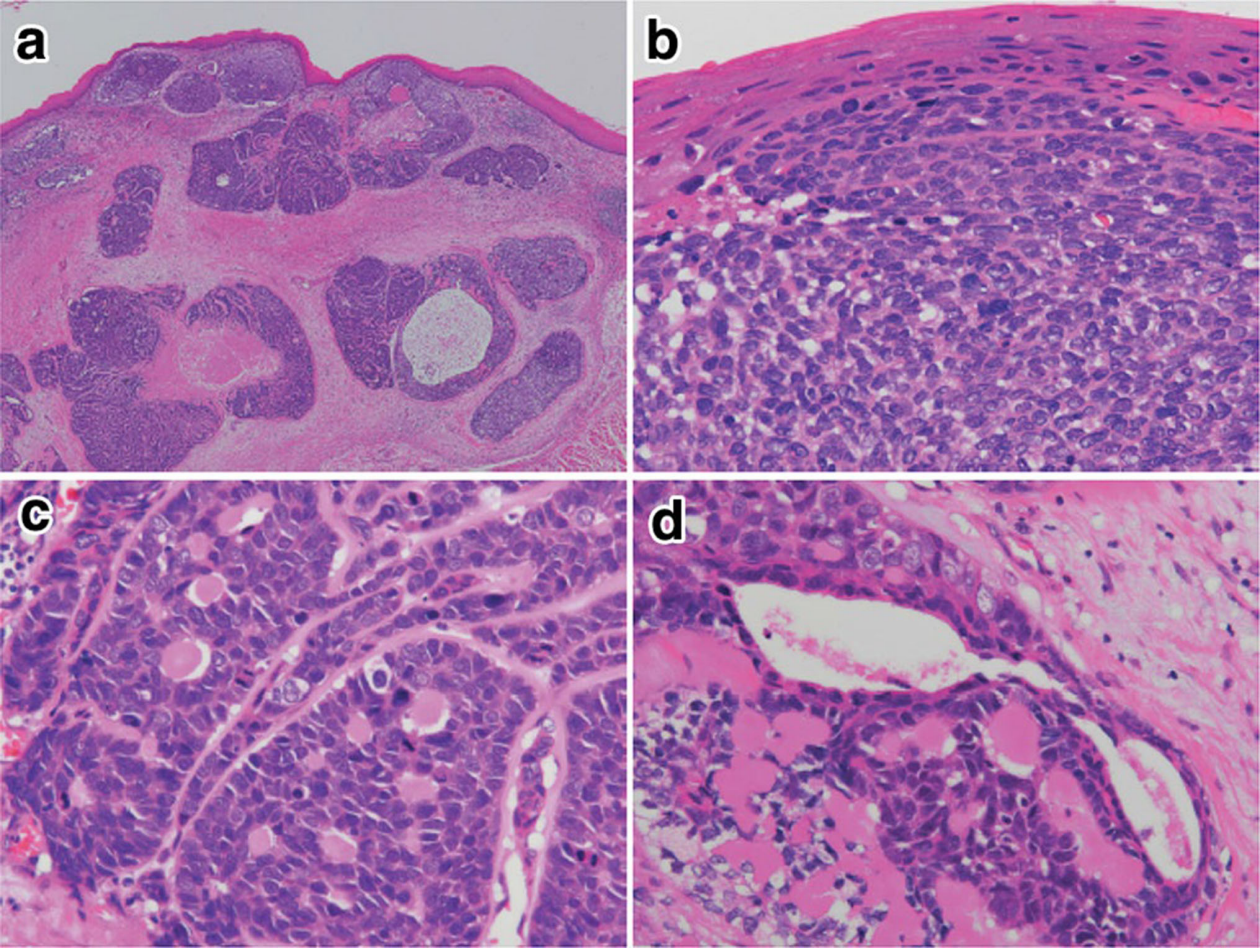
Basaloid squamous cell carcinoma. **a** Basaloid squamous cell carcinoma is often covered by a non-neoplastic squamous epithelium and formed solid nests with occasional cyst formation and comedonecrosis. **b** Tumor cells are similar to basal cells and form a solid nest under the stratified squamous epithelium. **c** The tumor cells exhibit a solid or trabecular arrangement. Eosinophilic basement membrane-like material deposits are present around and within the tumor nest. **d** Basaloid squamous cell carcinoma occasionally shows duct-like features

2. Basaloid squamous cell carcinoma (Fig. 9)

Basaloid squamous cell carcinoma comprises solid or trabecular pattern with basaloid tumor cells, occasionally forming adenoid cystic or ductular patterns. This entity is characterized by hyalinization, comprising basement membrane material, both within and outside the tumor nests. Squamous cell carcinoma component frequently coexists with basaloid component carcinoma especially in the intraepithelial part and is occasionally present in invasive areas.

**Fig. 10:**
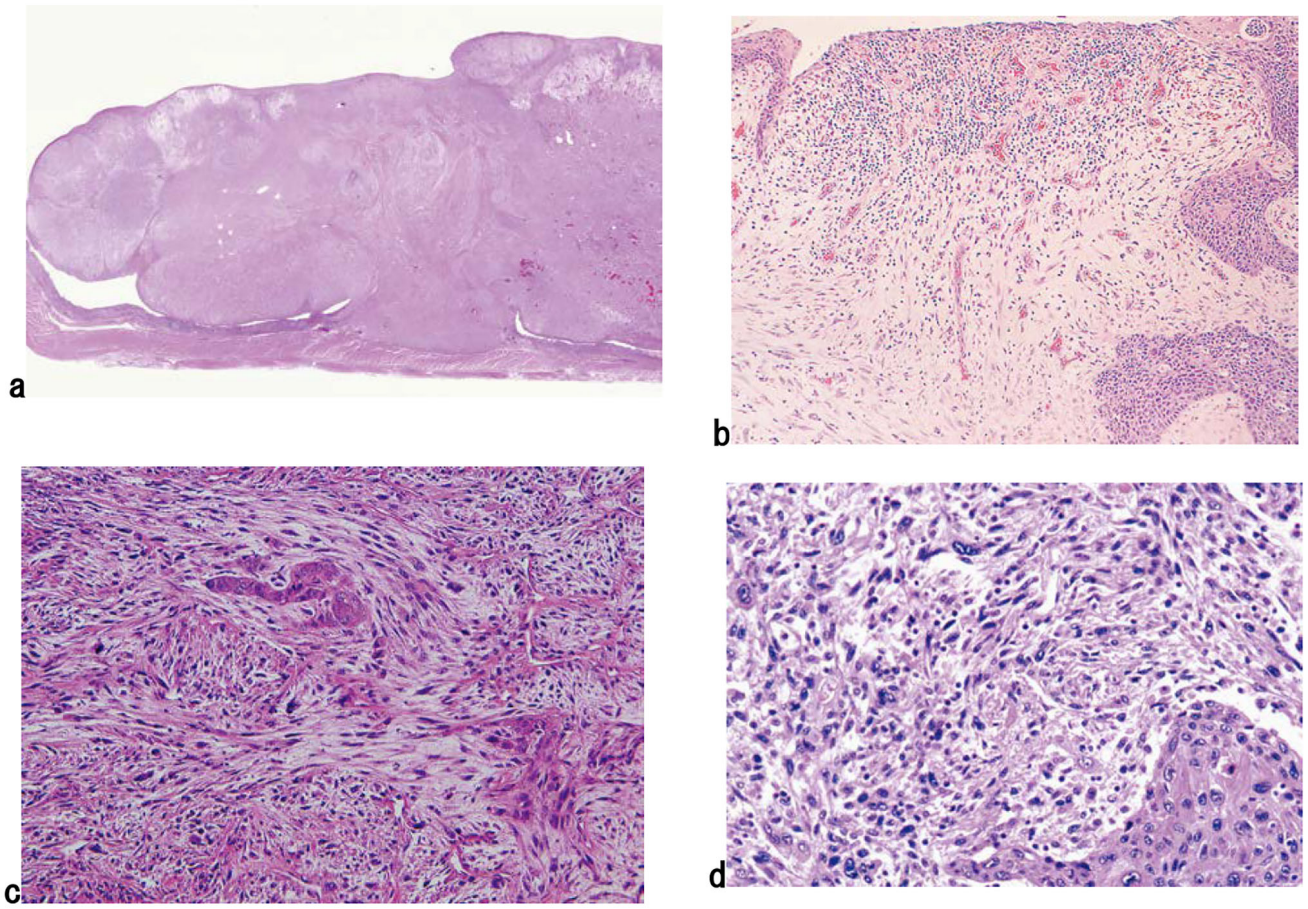
Carcinosarcoma. **a** A large tumor protrudes into the esophageal lumen but has not invaded deeply, in comparison with its size. **b** The polypoid tumor mainly consists of spindle cells, and its surface is covered by squamous cell carcinoma. **c** A large part of the tumor (sarcomatous component) is occupied by spindle-shaped tumor cells with scattered small foci of squamous cell carcinoma. **d** Tumor cells with prominent polymorphous nuclei are present in the sarcomatous component, which is similar to pleomorphic undifferentiated sarcoma

3. Carcinosarcoma (Fig. 10)

Carcinosarcoma is comprised of both carcinomatous and sarcomatous components. Sarcoma components of these tumors consist of spindle or pleomorphic tumor cells with a mesenchymal character, and occasionally contain bone or cartilage differentiation, resembling osteosarcoma or chondrosarcoma, respectively. This tumor often shows polypoid growth with a stalk and is characterized by the presence of squamous cell carcinoma in situ surrounding the stalk.

4. Adenocarcinoma

- Well differentiated
- Moderately differentiated
- Poorly differentiated.

Esophageal adenocarcinoma should be classified in the same manner as gastric carcinoma. Most tumors arise from Barrett’s esophagus. Esophageal adenocarcinoma rarely arises from the heterotopic gastric mucosa.

**Fig. 11:**
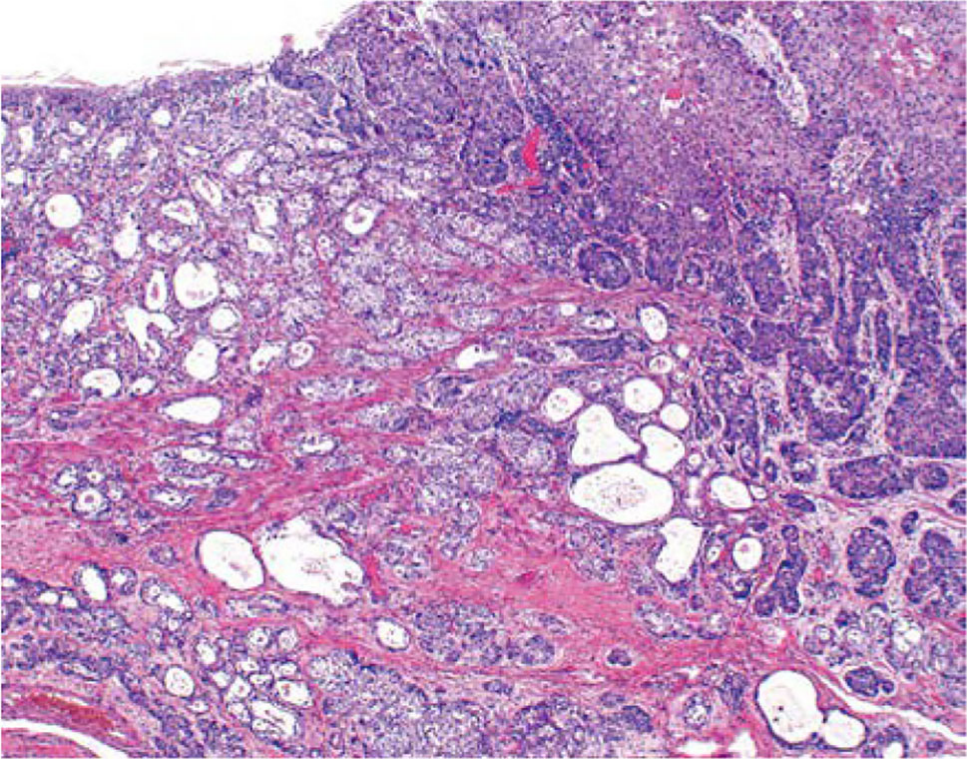
Adenosquamous carcinoma. Adenosquamous carcinoma consists of squamous cell carcinoma and adenocarcinoma. Invasive squamous cell carcinoma is present on the right side of the tumor, while adenocarcinoma is mainly on the left side

5. Adenosquamous carcinoma (Fig. 11)

Adenosquamous carcinoma contains components of both adenocarcinoma and squamous cell carcinoma, each of which can be easily recognized. When either of the two components is limited to a small area (< 20%), the tumor should be classified as a major component, with an additive note regarding the minor component, e.g., squamous cell carcinoma with an adenocarcinoma component.

**Fig. 12:**
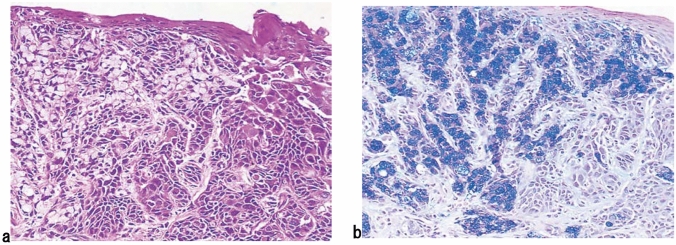
Mucoepidermoid carcinoma. **a** Signet-ring cell carcinoma is observed within squamous cell carcinoma. **b** Mucus in signet-ring cell carcinoma is stained blue by Alcian-blue/PAS. The serial section in Fig. 12a

6. Mucoepidermoid carcinoma (Fig. 12)

Mucus-containing cells (adenocarcinoma cells) are present in the squamous cell carcinoma nests. Typically, no distinct tubular structures are observed. Mucus-containing cells may be of the goblet cell or signet-ring cell type. Mucus is occasionally discharged into the stroma and the intercellular spaces.

**Fig. 13:**
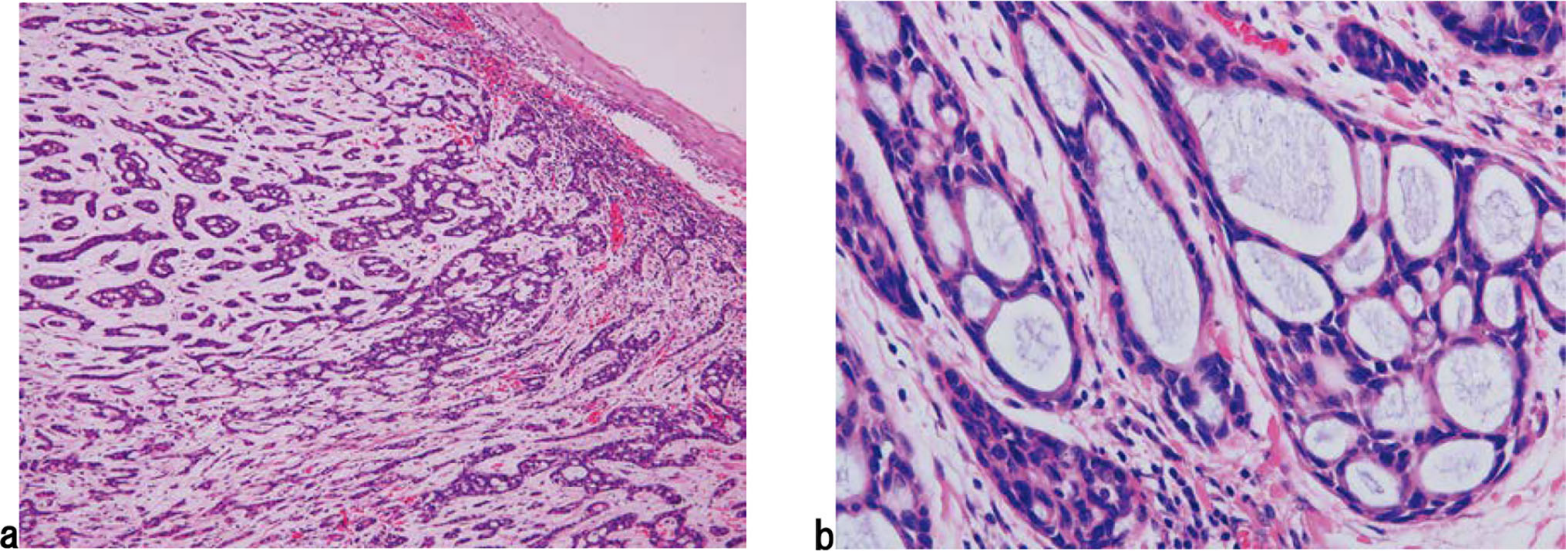
Adenoid cystic carcinoma. **a** A tumor with duct-like structures and a cribriform pattern has invaded downwards. **b** The histology of esophageal adenoid cystic carcinoma is almost the same as that of carcinoma of salivary gland origin. Nuclear atypia is more prominent in esophageal tumors than in salivary gland tumors

7. Adenoid cystic carcinoma (Fig. 13)

Adenoid cystic carcinoma is a rare tumor with the same histological appearance as that of salivary glands. Small cells with scant cytoplasm form cribriform, solid nest, or trabecular structures. Mucoid material within small cystic spaces inside and outside the tumor nests is stained light blue with Alcian-blue staining. Epithelial mucus production is not observed in the cribriform areas, unlike in adenocarcinomas with a cribriform pattern. A pattern of small duct-like structures is occasionally observed, in which the ducts consist of tumor cells aligned in a double layer containing epithelial mucus. This tumor should be carefully distinguished from basaloid squamous cell carcinoma.

**Fig. 14:**
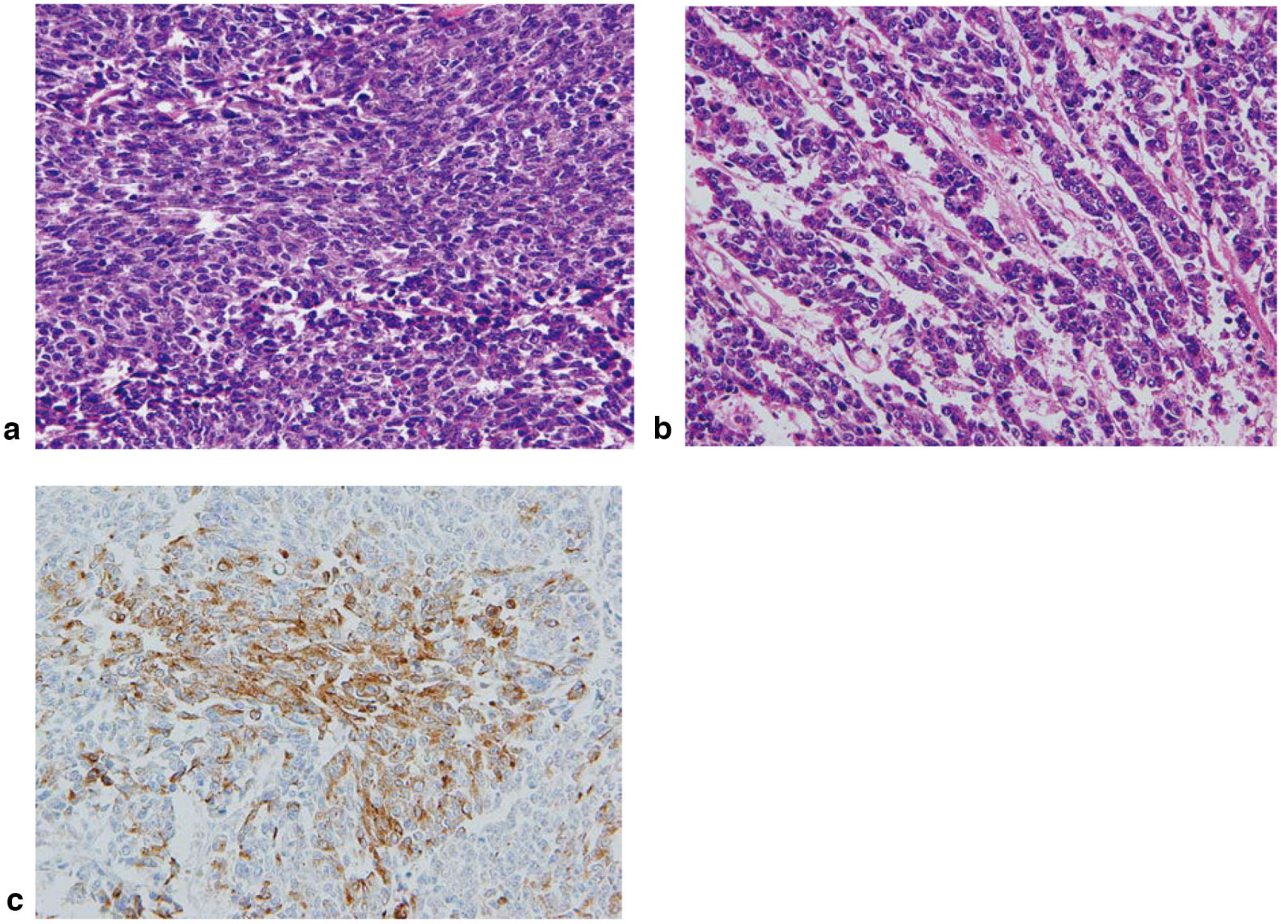
Neuroendocrine carcinoma, small cell type. **a** Small round cells with scant cytoplasm proliferate densely. **b** Tumor cells show an irregular arrangement with trabecular or ribbon-like patterns. **c** Immunohistochemically, the tumor cells are positive for chromogranin A, indicating differentiation to neuroendocrine cells

8. Neuroendocrine neoplasm (Fig. 14)

- Neuroendocrine tumor (NET) G1, G2, and G3
- Neuroendocrine carcinoma (NEC).

Neuroendocrine neoplasms include both neuroendocrine tumors (NET, formerly known as carcinoid tumors) and neuroendocrine carcinomas (NEC, formerly known as endocrine cell carcinomas). NET of the esophagus is rare and have the same histological appearance as NETs of other organs. Histologically, NEC forms nests of tumor cells of various sizes and occasionally shows trabecular or ribbon-like growth patterns or rosette formation. Esophageal NEC can be classified into two types: small cell type and large cell type. A definitive diagnosis requires immunohistochemical positivity for endocrine markers, such as chromogranin A, synaptophysin, and CD56 (N-CAM).

*Note:* Although tumors exhibiting neuroendocrine differentiation were previously classified as undifferentiated carcinoma, NEC should be distinguished from undifferentiated carcinomas based on the presence of neuroendocrine differentiation in the 12th edition.

9. Undifferentiated carcinoma

This tumor shows a medullary growth pattern consisting of small and large tumor cells without differentiation to any kind of cells, such as squamous, columnar, or neuroendocrine cells. Various kinds of evaluation including immunohistochemistry fail to detect any cell differentiations.

10. Others

This category includes malignant epithelial tumors that cannot be classified into any of the aforementioned groups.

##### 7.1.1.4 Non-epithelial tumor

**Fig. 15:**
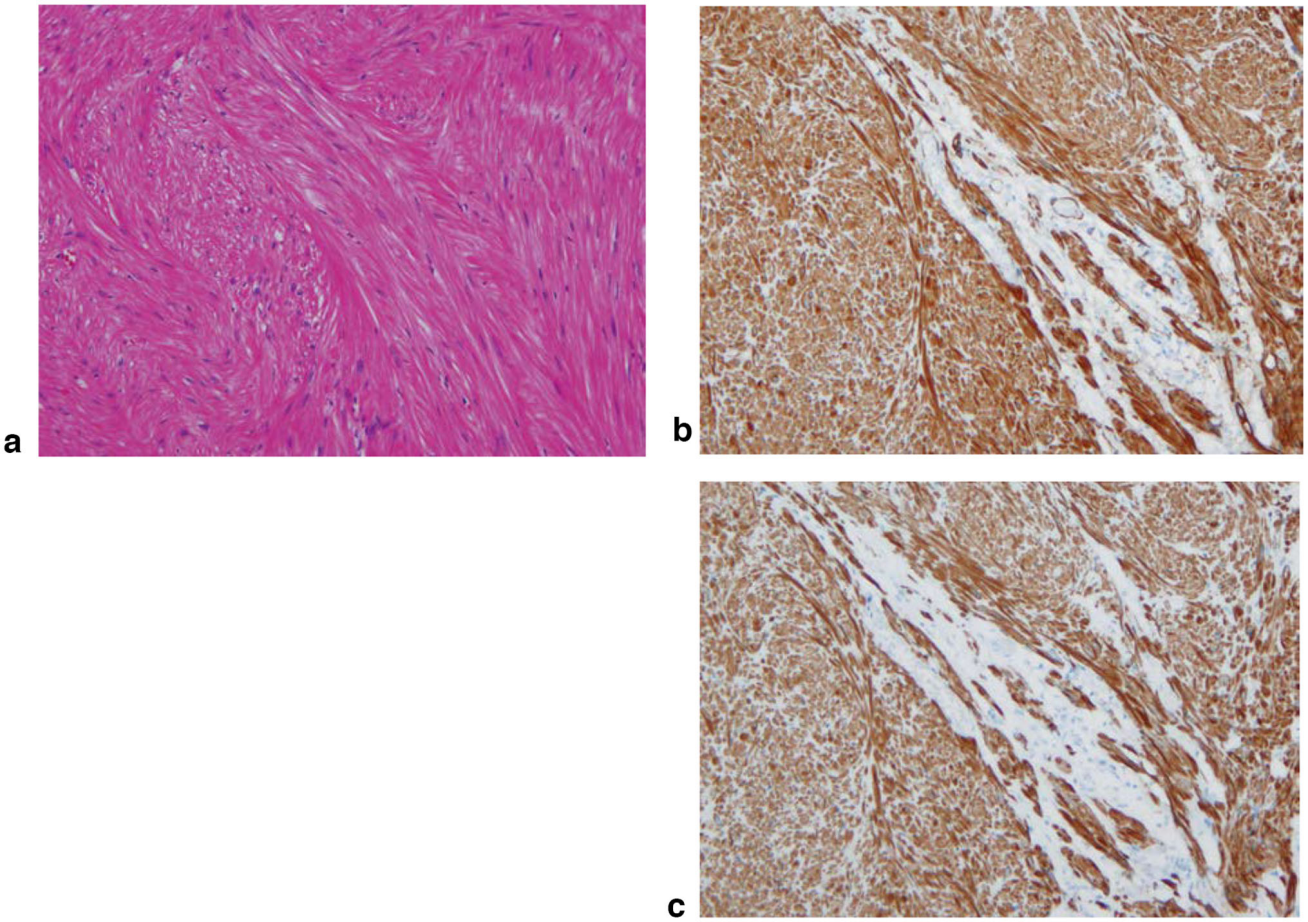
Leiomyoma. **a** Spindle cells with eosinophilic cytoplasm scantily grow in the fascicular pattern. **b** Immunohistochemically, the tumor cells are positive for α-smooth muscle actin. **c** Tumor cells are showing positive for desmin

1. Smooth muscle tumor (Fig. 15)

Leiomyoma and leiomyosarcoma

Histologically, the tumor shows an interlacing pattern of spindle-shaped tumor cells with elongated spindle nuclei, tapered ends, and cytoplasm with eosinophilic filaments. The epithelioid type shows a nest-like growth pattern composed of round tumor cells with eosinophilic or clear cytoplasm. These tumors are often small nodules in the muscularis propria of the lower esophagus, muscularis mucosa, or middle esophagus. This tumor shows positive immunohistochemical reactions for smooth muscle actin and desmin, and a negative reaction for KIT (CD117). The differential diagnosis between benign and malignant tumors is based on cellularity, nuclear pleomorphism, and mitosis. Leiomyoma has no nuclear pleomorphism or mitotic figures and exhibits low cellularity, whereas leiomyosarcoma has abundant mitotic figures and exhibits high cellularity.

**Fig. 16:**
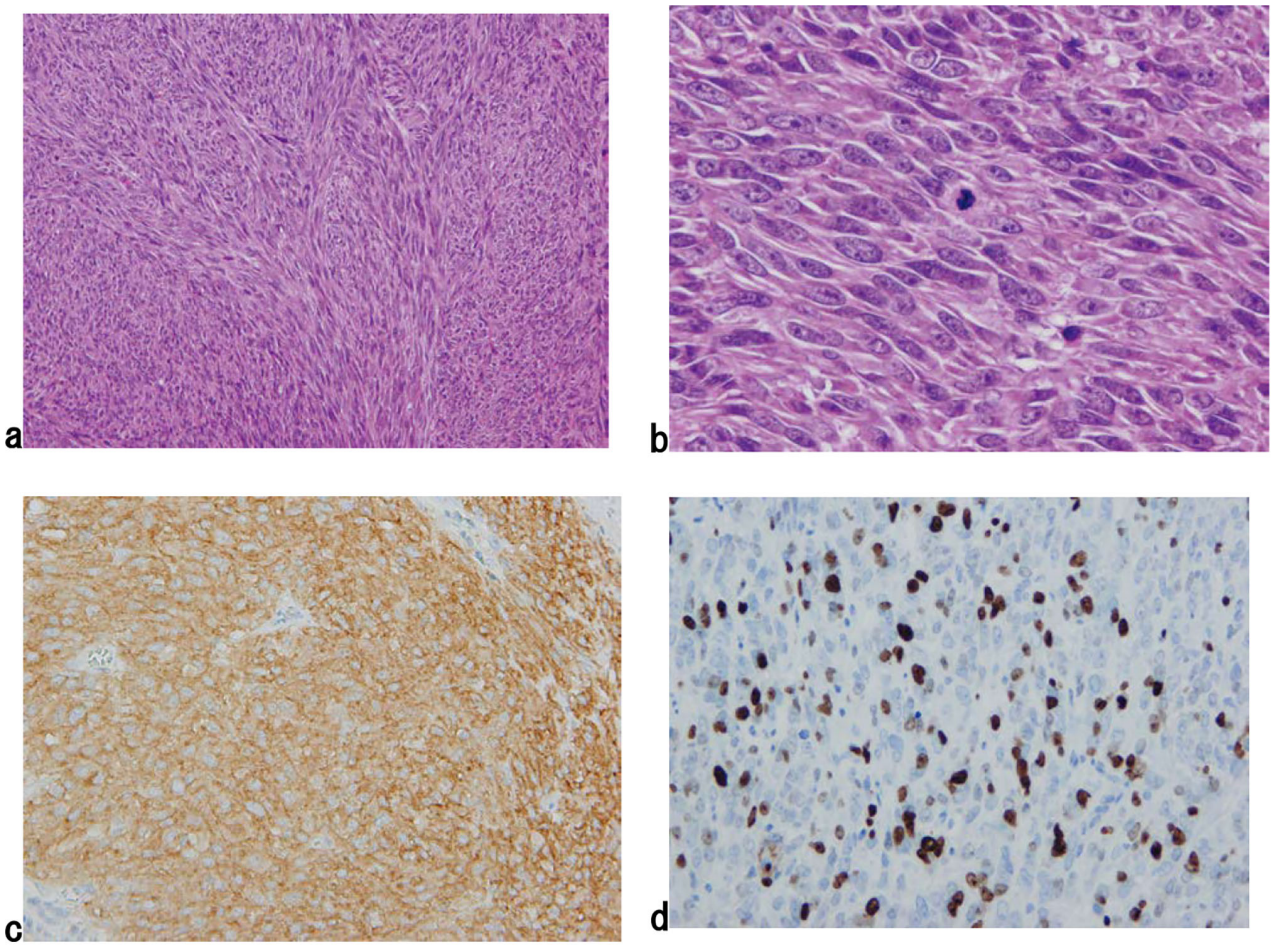
A gastrointestinal stromal tumor (GIST). **a** Spindle cells densely proliferate with a fascicular arrangement. **b** Mitotic figures are shown among tumor cells with plump nuclei. **c** Immunohistochemically, the tumor cells are positive for KIT (CD117). **d** The Ki-67 (MIB-1) labeling index was 30%, indicating a high-risk tumor

2. Gastrointestinal stromal tumor (GIST) (Fig. 16)

This tumor has specific features of thick spindle cells or epithelial cells. It is occasionally difficult to differentiate GIST from smooth muscle tumors without immunohistochemical (IHC) staining. Immunohistochemically, GIST exhibits KIT (CD117) and/or DOG1 positivity. CD34 is positive in approximately 80% of GISTs.

*Note:* Risk assessment follows the Guidelines for the diagnosis and treatment of GIST [5].

4. Neurogenic tumor

**Fig. 17:**
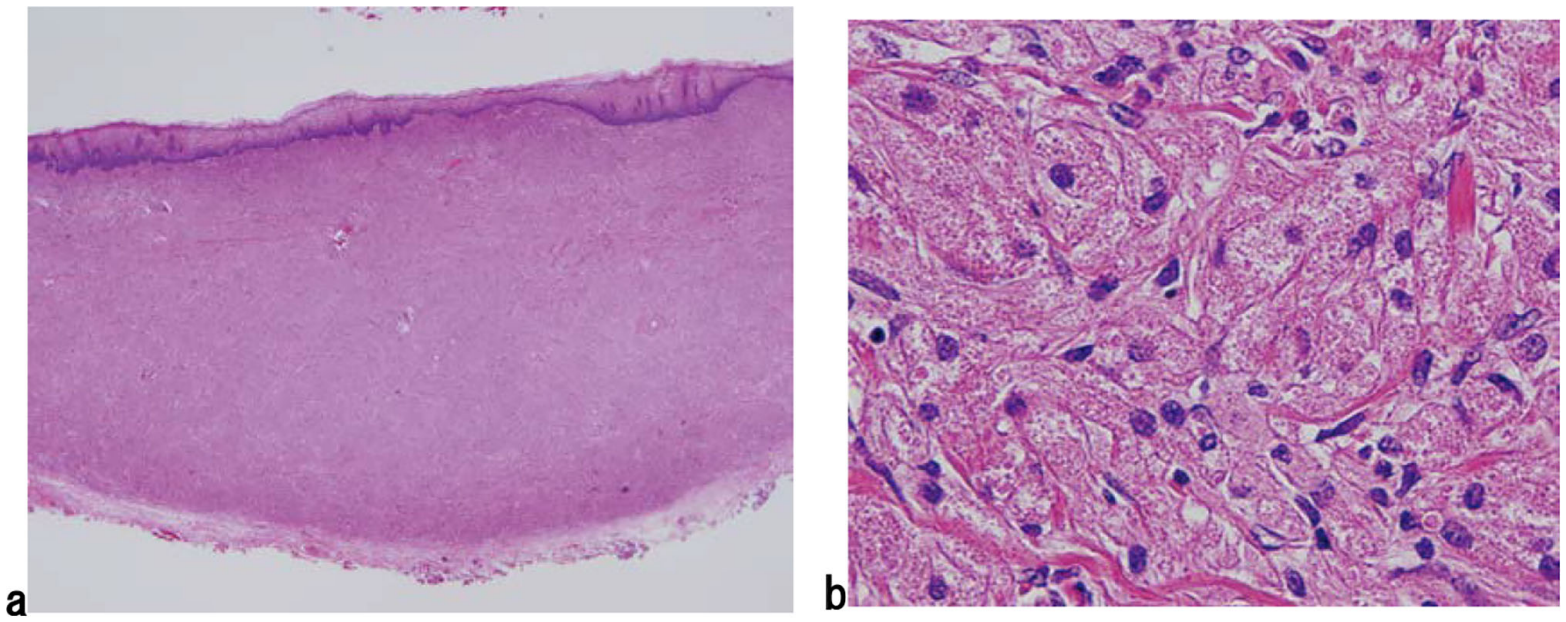
Granular cell tumor. **a** The tumor has grown mainly within the lamina propria mucosa and is covered by stratified squamous epithelium. **b** The large and round tumor cells exhibit abundant granular and eosinophilic cytoplasm

Neurogenic tumors include schwannoma, neurofibroma, and granular cell tumor (Fig. 17). Granular cell tumor is composed of large round cells with abundant eosinophilic granules in the cytoplasm, forms solid nests of various sizes, and grows predominantly in the lamina propria mucosa or submucosa. Stratified squamous epithelium commonly covers these tumors and occasionally exhibits pseudoepitheliomatous hyperplasia.

5. Others

Hemangioma, lymphangioma, and lipoma.

##### 7.1.1.5 Lymphoid tumor

There have been few case reports of B-cell lymphoma of the esophagus. The latest version of the WHO classification is used for esophageal malignant lymphoma [2].

##### 7.1.1.6 Other malignant tumors

**Fig. 18:**
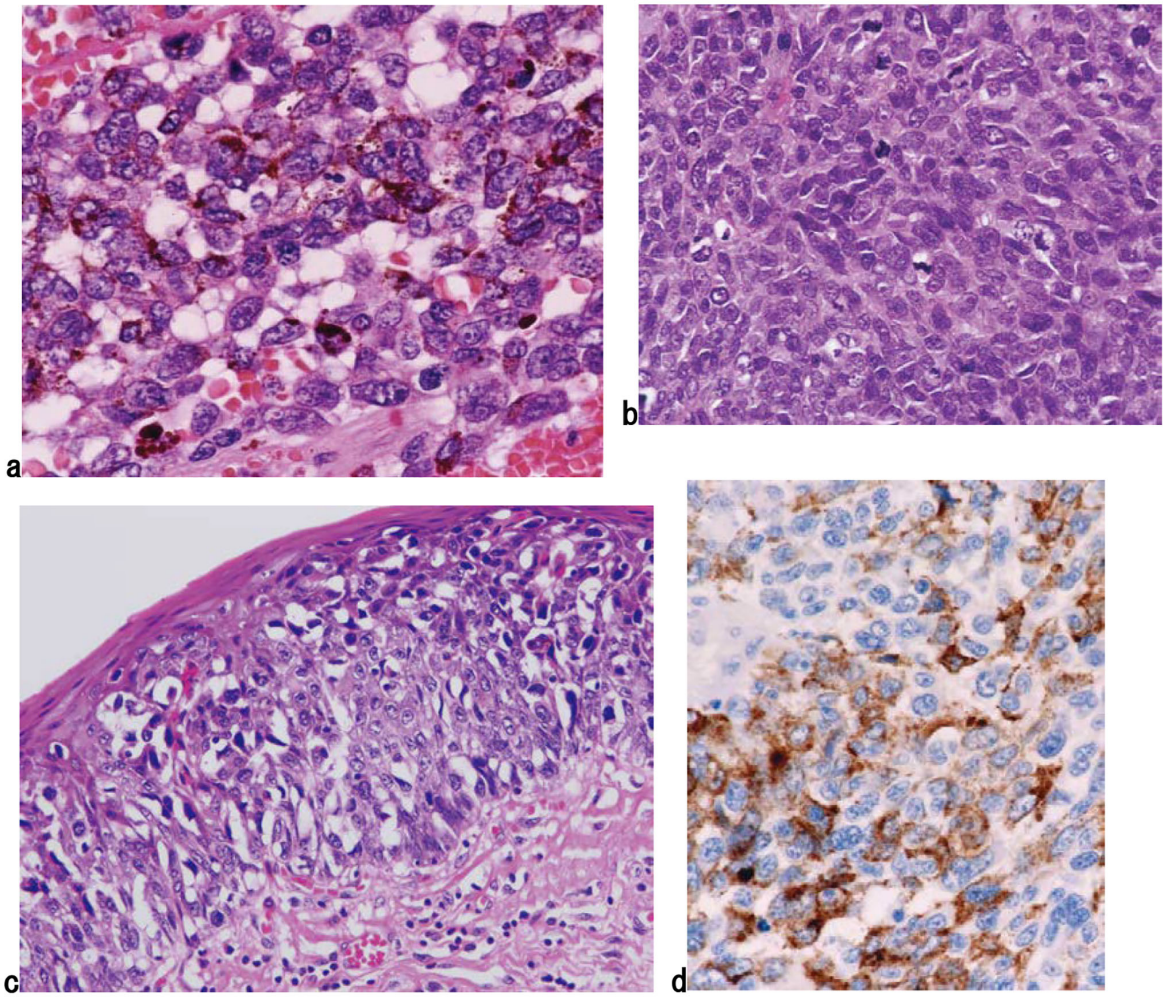
Malignant melanoma. **a** The short spindle-shaped or round tumor cells with abundant melanin granules have proliferated densely. **b** There are no melanin granules are observed in amelanotic melanoma. **c** Tumor cells with a clear cytoplasm show intraepithelial spread adjoining an invasive tumor. **d** Immunohistochemically, the tumor cells are positive for HMB-45 (Human Melanoma Black-45)

1. Malignant melanoma (Fig. 18)

To diagnose primary malignant melanoma, it is necessary to histologically identify tumor cells that produce melanin granules that proliferate in the basal layer of the squamous epithelium.

2. Others

Choriocarcinoma has been reported in the esophagus.

##### 7.1.1.7. Tumor-like lesions

Heterotopic gastric mucosa

Heterotopic sebaceous gland

Glycogenic acanthosis

Fibrovascular polyp^*Note 1*^

*Note 1:* As some of these tumors have chromosomal abnormalities or amplifications of the MDM2 gene, they should be recognized as neoplasms and differentiated from liposarcomas.

#### 7.1.2. Other findings to be described

1. Metastatic or invasive cancer from other organs
2. Co-existing tumor
  Leiomyoma and others
3. Other non-neoplastic lesions
  Barrett’s esophagus, Achalasia, and others

#### 7.1.3. Report of pathological findings

All of the above-mentioned factors should be described, and the attachment of a figure showing a general view of the resected specimen with depths of tumor invasion and lymphovascular invasion is recommended. It is better to attach a schematic showing the pathological findings on the cut surface, when necessary.

## 8. Esophagogastric junction and Barrett’s esophagus

Definitions and description methods for the esophagogastric junction and Barrett’s esophagus.

### 8.1. Definition of the esophagogastric junction (EGJ)

The EGJ should be defined systematically following criteria, such as endoscopic findings, that should be prioritized over those obtained using other diagnostic modalities.

1. Endoscopic findings

- Lower margin of palisading small vessels
- If the palisading small vessels are unclear, the oral margin of the longitudinal folds of the greater curvature of the stomach is defined as the EGJ.

2. Upper gastrointestinal imaging (UGI)

- Narrowest locus of the lower esophagus
- The upper end of the longitudinal folds

3. Pathological assessment

- Macroscopic definition of the surgically resected specimen: The EGJ should be defined macroscopically as the point wherein the luminal caliber changes in the area where the tubular esophagus is connected to the vestibular lumen of the stomach.
- Microscopic definition of the surgically or endoscopically resected specimen: The EGJ should be defined based on the presence of histological features found in the esophagus (squamous epithelium [including squamous island], proper esophageal glands and their ducts, double-layer muscularis mucosa, or palisading small vessels above muscularis mucosa).

#### 8.1.1 Endoscopy (Fig. 19)

Commentary:

The esophageal sphincters are located at both the proximal and distal ends of the esophagus and are referred to as the upper esophageal sphincter (UES) and lower esophageal sphincters (LES), respectively. The propria mucosa layer of these two loci contains small palisading vessels that penetrate the muscularis mucosa layer after branching from the submucosal vessels. As the sphincter muscles are characteristic features of the esophagus, the esophagus is defined as the tract between the UES’ upper margin and the LES’ lower margin. As the LES’ lower margin is defined as the EGJ, the EGJ level can be defined as the lower margin level of the lower palisading vessels. Considering this interpretation, most Japanese experts endoscopically define the EGJ as the lower margin of the lower palisading vessels. Endoscopic observation of palisading small vessels should be performed under conditions, wherein the lower esophagus is adequately stretched after suctioning the air from inside of the stomach and the examinee has inhaled deeply.

Palisading small vessels may be difficult to distinguish in endoscopic examinations of patients with gastroesophageal reflux disease or long-segment Barrett’s esophagus (LSBE). In addition, constant esophageal stretching cannot be attained with deep inhalation if the patient is sedated. Most Western experts have long defined the EGJ as the upper end of the longitudinal folds of the stomach according to the Prague C&M Classification developed by the International Working Group for the Classification of Esophagitis (IWGCO) in 2006 [6]. Endoscopic observation of the upper ends of the longitudinal folds should be performed with optimal decompression of the stomach through suction; however, “optimal decompression” has not been strictly defined. The oral margins of the longitudinal folds of the stomach cannot be reliably defined, because the air volume inside the stomach varies. Thus, the diagnostic concordance rate for detecting short-segment Barrett’s esophagus (SSBE) extending to < 1 cm lengths is low. Moreover, the gastric folds tend to be smaller in the presence of atrophic gastritis, which is more common in Japan. Considering these points, the upper ends of the longitudinal folds can be difficult to define.

When making a diagnosis, the EGJ should be defined with a comprehensive interpretation of both the palisading small vessels and longitudinal folds. In Japan, LSBE is less and atrophic gastritis more common, respectively. Therefore, in the Japanese Classification of Esophageal Cancer, the lower margins of the palisading vessels are primarily used to define the EGJ. The upper end of the longitudinal folds was used as a secondary criterion in cases wherein the palisading vessels are difficult to distinguish.

**Fig. 19:**
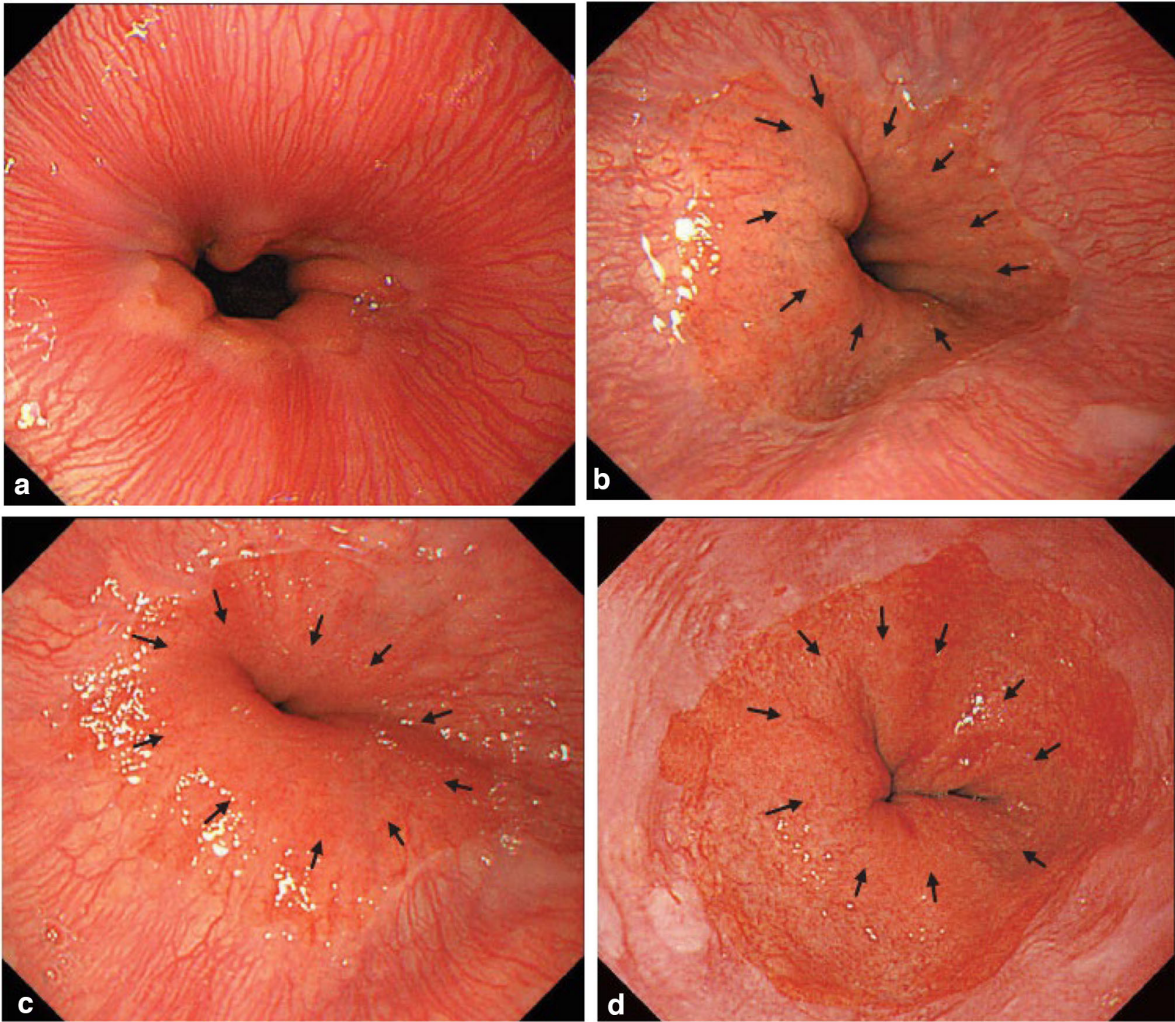
**a** The lower margin of the palisading small vessels and the oral margin of the longitudinal folds of the greater curvature of the stomach are both clearly visible and coincident at the same level. In such cases, this site of coincidence is defined as the EGJ and is nearly identical to the SCJ. **b** The lower margin of the palisading small vessels and the oral margin of the longitudinal folds of the greater curvature of the stomach are both clearly visible and coincident at the same level. In such cases, this site of coincidence is defined as the EGJ (black arrow). The gap between the SCJ and the EGJ is diagnosed as Barrett’s esophagus. **c** The palisading small vessels are visible, but the longitudinal folds are unclear. The lower margin of the palisading small vessels is defined as the EGJ (black arrow). The gap between the SCJ and the EGJ is diagnosed as Barrett’s esophagus. **d** The longitudinal folds are visible, but the lower margin of the palisading vessels is unclear. The upper oral margin of the longitudinal fold is defined as the EGJ (black arrow). The gap between the SCJ and the EGJ is diagnosed as Barrett’s esophagus

#### 8.1.2 X-ray: upper gastrointestinal imaging (Figs. 20, 21, 22)

**Fig. 20:**
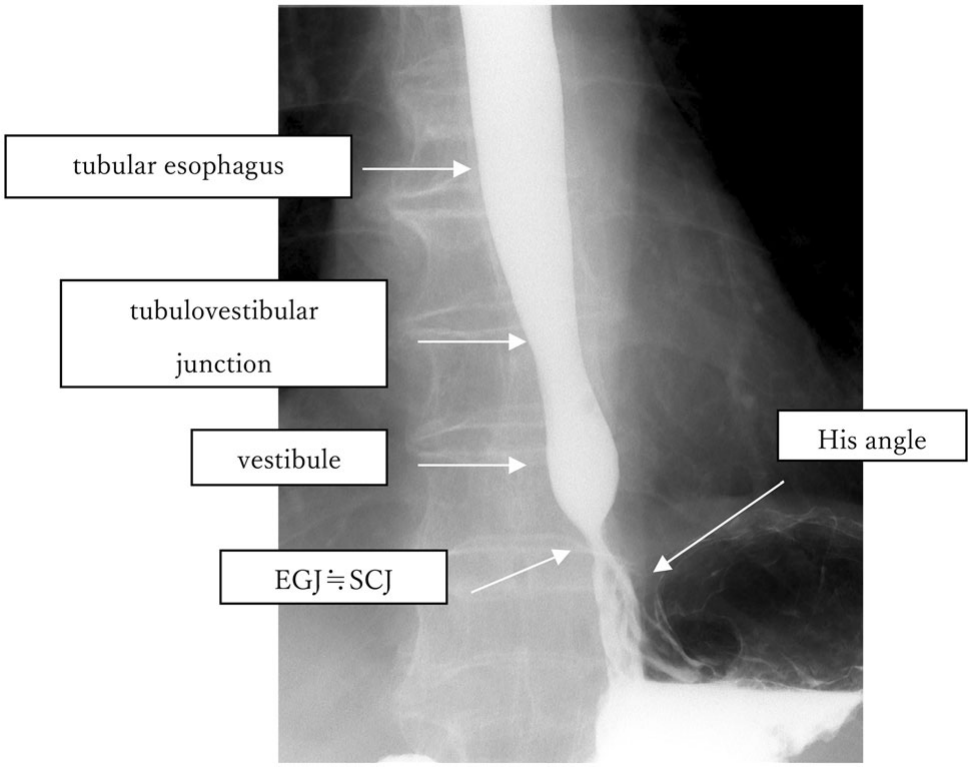
Barium contrast image of a normal EGJ

**Fig. 21:**
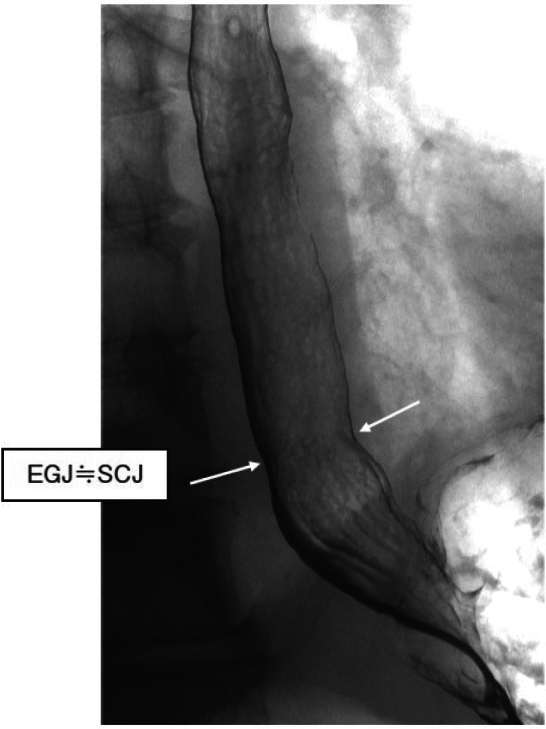
Barium contrast image of the EGJ in a subject with a hiatal hernia

**Fig. 22:**
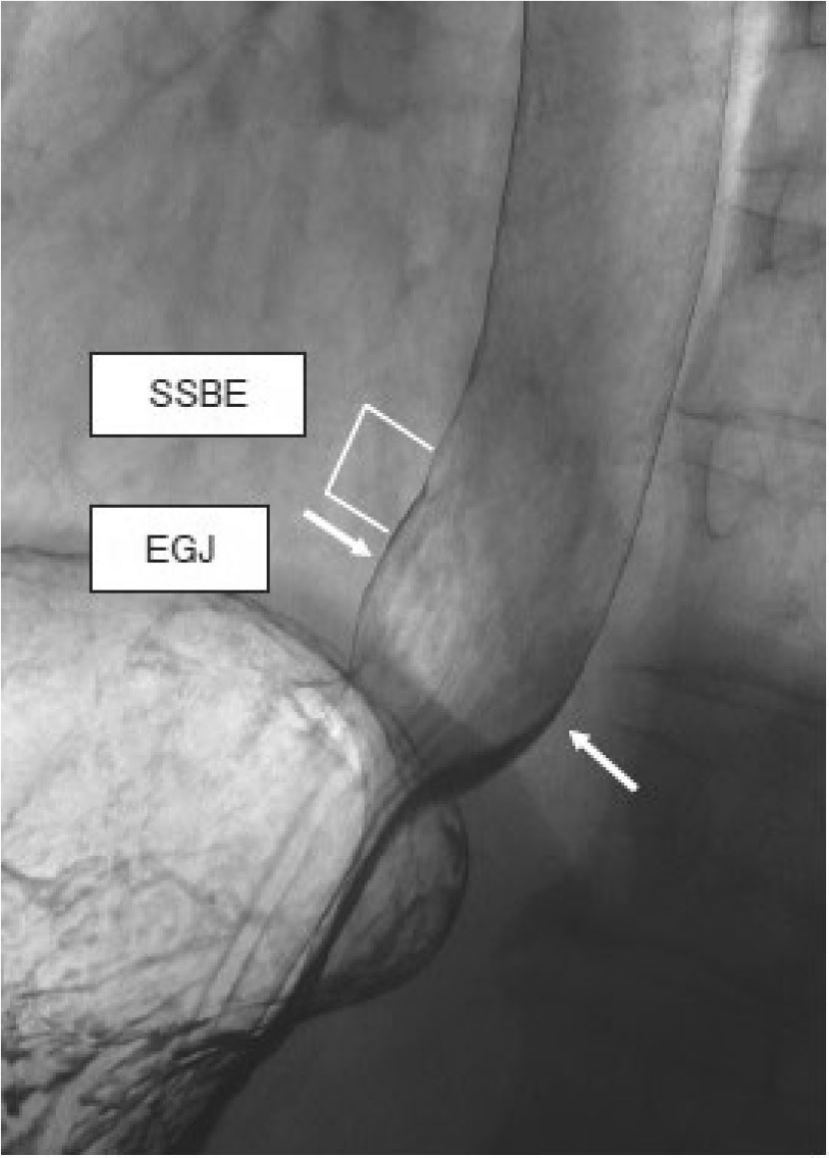
Barium contrast image of the EGJ in an individual with Barrett’s mucosa

- The EGJ is defined as the narrowest locus of the lower esophagus (Fig. 20).
- In cases of a sliding hiatal hernia, the EGJ should be identified as the oral margin of the longitudinal folds (Fig. 21).
- In the presence of Barrett’s esophagus, the SCJ is located on the oral side of the EGJ, and Barrett’s mucosa appears as mucosa containing reticular structures in a double-contrast study (Fig. 22). The EGJ is identified as the oral margin of the longitudinal folds.

Commentary:

In a barium contrast series, the esophagus can be divided into two segments, the tubular and vestibular (or saccular) esophagus, with a junction called the tubulovestibular junction, where the lumen of the esophagus gradually narrows. The Angle of His designates the angle formed by the esophagus and fornix of the stomach. In healthy individuals, the Angle of His and the EGJ is identical to the narrowest part of the lower esophageal lumen. A muscular fiber lies between the left side of the esophagus and the entry of the stomach, resulting in acuteness of the angle of His. This fiber is located inside the propria muscle of the stomach and is called the “sling fiber”. In healthy individuals, the EGJ level is nearly identical to that of the SCJ level.

Commentary:

In cases with a hiatal hernia, a circular “neck” of the esophageal lumen is observed close to the hiatus or at the lower level of the pleural cavity during a barium contrast study, and this neck is referred to as a mucosal ring (B ring) or “Z-line.” Although this neck is referred to as a “ring,” the ring is not formed by a circular fiber of the muscular propria, but is presumably formed by the sling fiber and is prominent on the left or fornix side of the esophagus. In cases of a hiatal hernia, the EGJ is usually imaged at the oral margin of the longitudinal fold of the stomach.

Commentary:

The “circular neck” of the esophagus accompanying a hiatal hernia is often obscured in the presence of Barrett’s mucosa. Therefore, the EGJ is usually defined as “the oral margin of the longitudinal folds of the stomach” in patients with Barrett’s mucosa.

#### 8.1.3 Pathological study

Commentary:

The EGJ was previously defined as “the border between the muscular structures of the esophagus and stomach”; however, histological discrimination of the muscular structures in each area is difficult. Therefore, pathological definition of the EGJ depends on the macroscopic findings or histological features of the mucosal or submucosal layer. Thus, the surgical specimen must be adequately extended and optimally fixed to define the EGJ and the histological structures must be examined using a full section of the entire area of the EGJ. If small palisading vessels are visible in a fresh specimen, the anal margins of the vessels should be marked before histological inspection.

Macroscopically, the EGJ in the surgically resected specimen should be identified as the point at which the luminal caliber changes in the area where the tubular esophagus is connected to the vestibular lumen of the stomach. In patients with hiatal hernia, the EGJ should be decided not only macroscopically but also based on the histological findings, because the caliber site change is unclear.

Histologically, the EGJ in the surgically or endoscopically resected specimen should be identified based on the findings which anatomically belong to the esophagus (squamous epithelium [including squamous island], proper esophageal glands and their ducts, double-layer muscularis mucosa ^*Note 1*^, or palisading small vessels ^*Note 2*^). According to the distribution of the aforementioned histological features, the EGJ is defined as the most distal site where any of them are observed.

When the macroscopic EGJ is almost identical to the SCJ, there is no finding of Barrett’s esophagus. When the macroscopic EGJ is not the same as the SCJ, it leads to having Barrett’s esophagus; however, these decisions should be made comprehensively based on macroscopic and histological findings.

*Note 1:* Smooth muscle fibers are occasionally generated in the lamina propria of the esophageal squamous mucosa. Such newly generated smooth muscle fibers form layer structure, defined as superficial muscularis mucosae (SMM), above original muscularis mucosa, called deep muscularis mucosae (DMM). The discrimination of SMM from DMM is occasionally difficult because of the fusion of both layers.

*Note 2:* Histologically, when the veins with diameters of greater than 100 µm are found above the muscularis mucosae of the lower esophagus, they may be considered as palisading vessels. They can be used for the pathological assessment of Barrett’s esophagus.

### 8.2. Barrett’s esophagus (Figs. 23, 24, 25, 26)

#### 8.2.1. Definition of Barrett’s esophagus

An esophagus containing Barrett’s mucosa should be designated as Barrett’s esophagus. Barrett’s mucosa refers to the columnar epithelium expanding from the stomach to the esophagus, irrespective of the presence of intestinal metaplasia ^*Note 1*^.

**Fig. 23:**
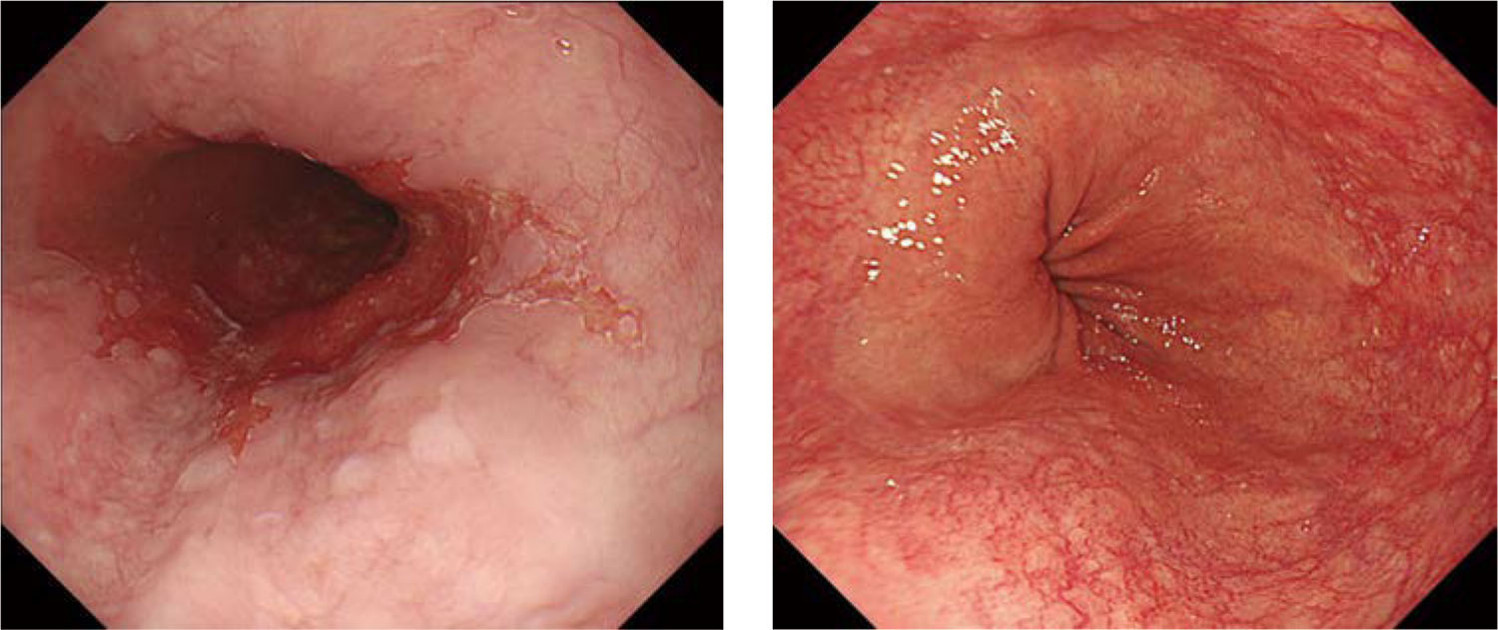
Long-segment Barrett’s esophagus (LSBE). Barrett’s esophagus at C9M10 is classified as an LSBE in both the former and current editions

**Fig. 24:**
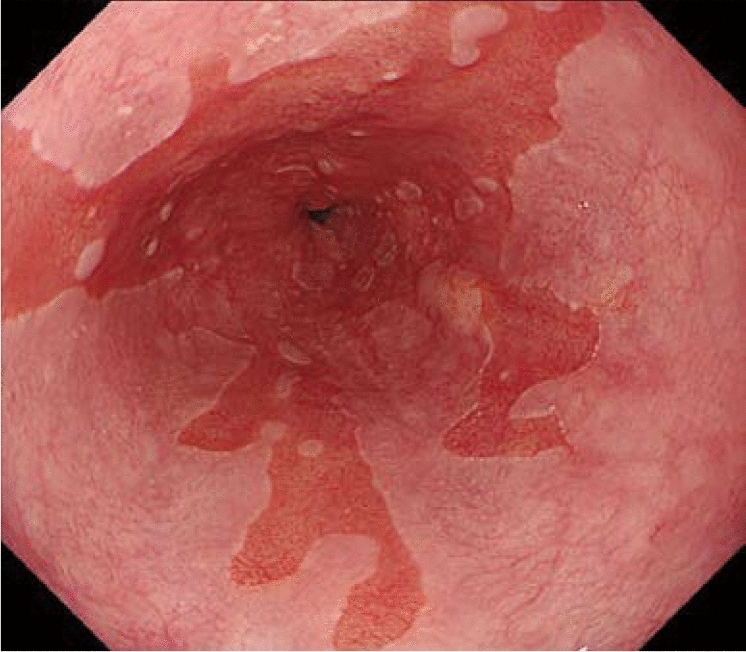
Long-segment Barrett’s esophagus (LSBE). Barrett’s esophagus at C2M4 was classified as short-segment Barrett’s esophagus (SSBE) in the former edition, but is classified as LSBE in the current edition

**Fig. 25:**
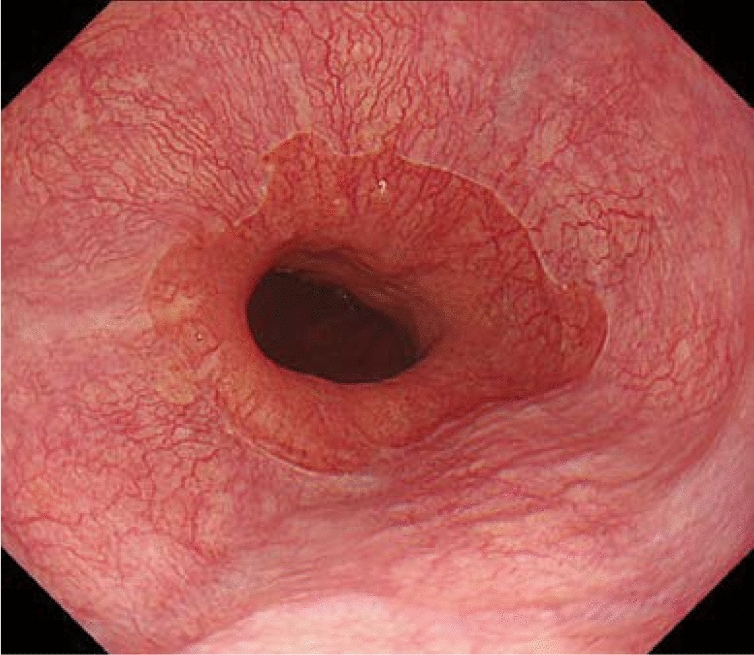
Short-segment Barrett’s esophagus (SSBE). Barrett’s mucosa with < 3 cm in maximum length is defined as SSBE

*Note 1:* The presence of Barrett’s mucosa extending longitudinally for 3 cm or more, even if it is not circular, is called long-segment Barrett’s esophagus (LSBE) (Fig. 23). In the previous Japanese classification of Esophageal Cancer 11th edition [7], only circular Barrett’s mucosa extending ≥3 cm had been defined as LSBE. For instance, C2M4 (Prague classification) was defined as short-segment Barrett’s esophagus (SSBE) in the 11th edition. In the 12th edition, the definition of LSBE has been changed to M3 or higher (Fig. 24) [8]. Therefore, in the 12th edition, C2M4 is defined as LSBE. In contrast, Barrett’s mucosa <3 cm in maximum length is defined as an SSBE (Fig. 25).

**Fig. 26:**
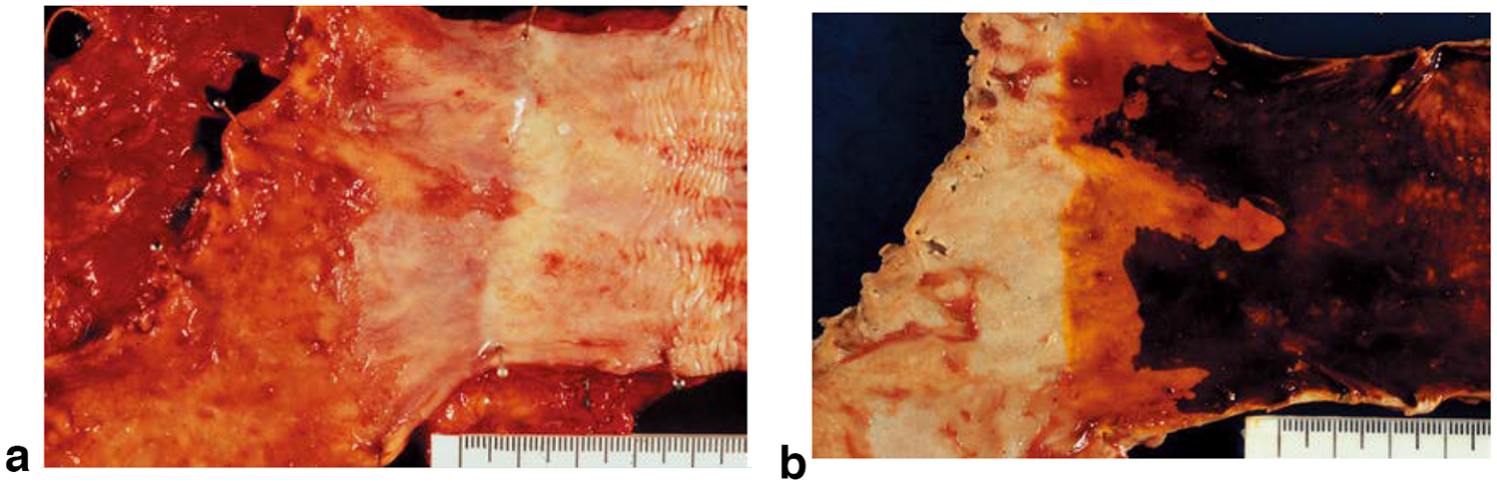
**a** Barrett’s esophagus and adenocarcinoma in Barrett’s esophagus, 0-IIb. The squamocolumnar junction shows an irregularity, with a tongue-like extension of the columnar-lined mucosa toward the esophagus. **b** Iodine-stained specimen. Iodine staining clearly shows a tongue-like extension of columnar-lined mucosa measuring 30 mm (long-segment Barrett’s esophagus: LSBE). A cancerous lesion was detected pathologically in Barrett’s mucosa, but is invisible macroscopically. 0-IIb, pT1a-SMM

#### 8.2.2. Histological types of Barrett's mucosa (Figs. 27, 28, 29)

**Fig. 27:**
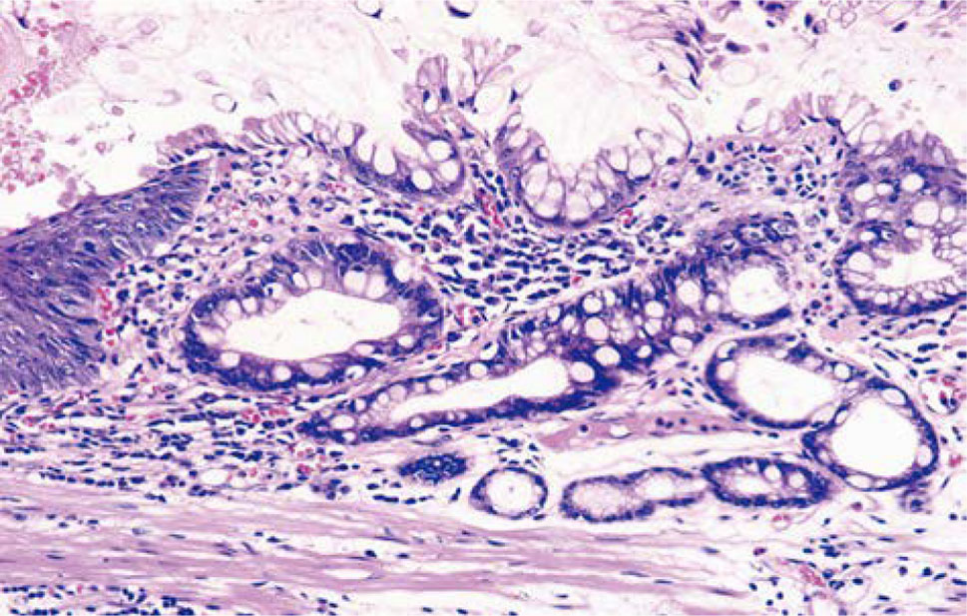
Barrett’s mucosa (specialized columnar epithelium). The esophagus is covered by specialized columnar epithelium with intestinal metaplasia. Squamous epithelium is visible on the distal side (squamous island)

**Fig. 28:**
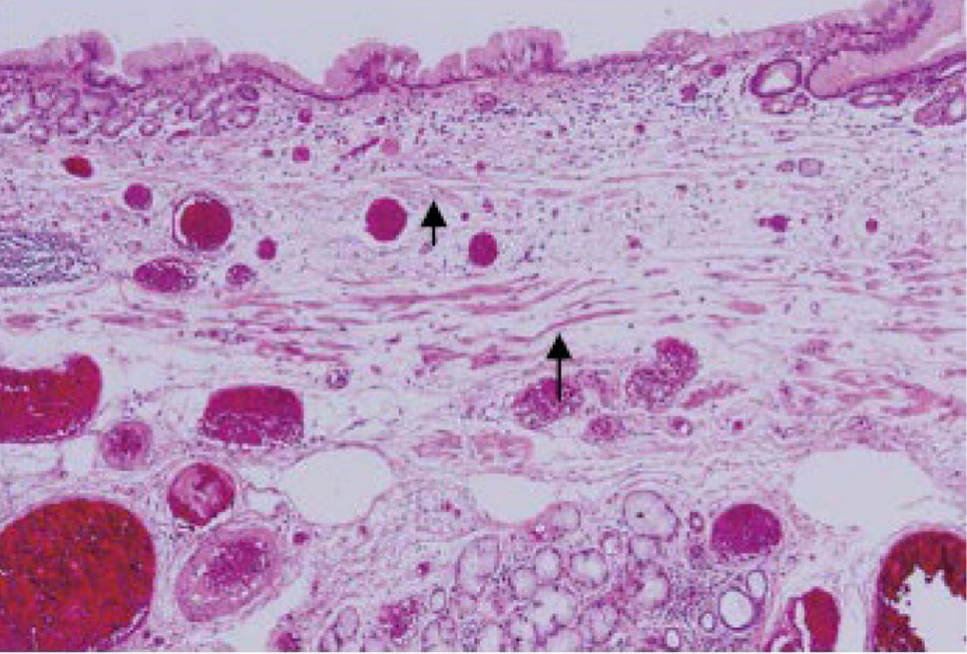
Barrett’s mucosa (junctional type). A double-layered muscularis mucosa (arrows) is present beneath the overlying columnar epithelium. Esophageal glands are also observed in the submucosal layer. Overlying columnar epithelium is of the cardiac gland type

**Fig. 29:**
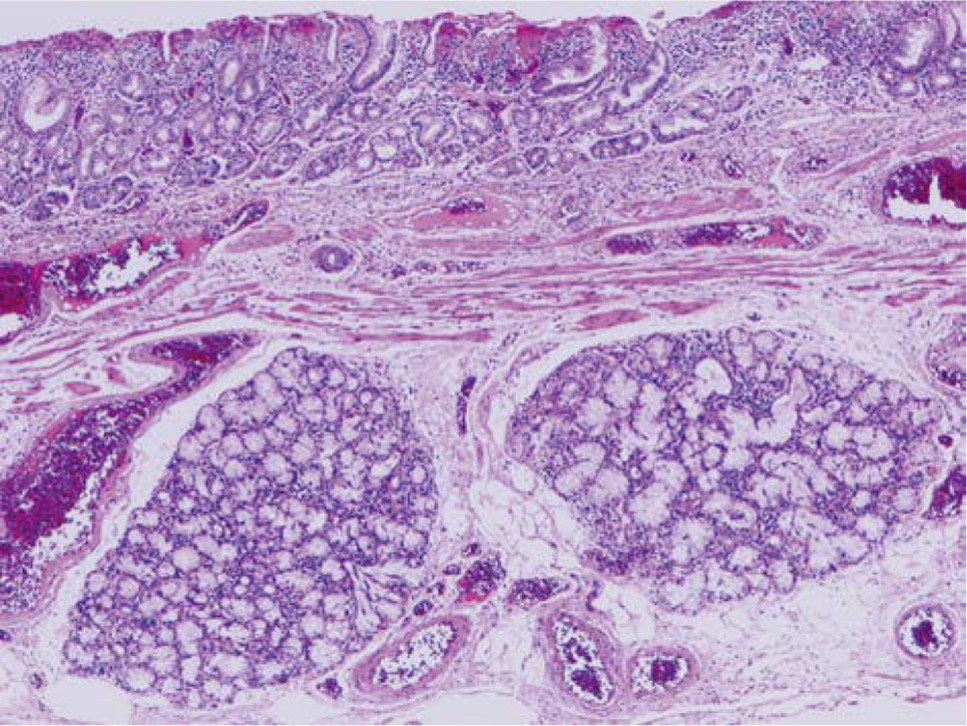
Barrett’s mucosa (gastric fundic type). The mucosa consists of fundic gland-type columnar epithelium. Esophageal glands proper are observed in the submucosal layer

Barrett’s mucosa is comprised of the following types of the epithelium:

- Specialized columnar epithelium (SCE).^*Note*^: Columnar epithelium with intestinal metaplasia. Paneth cells rarely appear (Fig. 27)
- Junctional type: Columnar epithelium similar to the cardiac gland mucosa, sometimes accompanied by parietal cells (Fig. 28)
- Gastric fundic type: Columnar epithelium similar to the fundic gland with chief or parietal cells (Fig. 29)

Note: When intestinal metaplasia is found in the Barrett’s mucosa, it should be recorded, for example, Barrett’s mucosa SCE (+).

Types of Barrett’s mucosa.

### 8.3 Adenocarcinoma in Barrett’s esophagus (Figs. 30 and 31)

Adenocarcinoma in Barrett’s esophagus is adenocarcinoma arising from Barrett’s mucosa. It differs from adenocarcinoma arising from the gastric mucosa of the esophagogastric junction (gastric cancer) in terms of origin. However, it is often difficult to discriminate them in large tumors. Histological classification of the adenocarcinoma in Barrett’s esophagus complies with the Japanese classification of gastric cancer [1, 9].

**Fig. 30:**
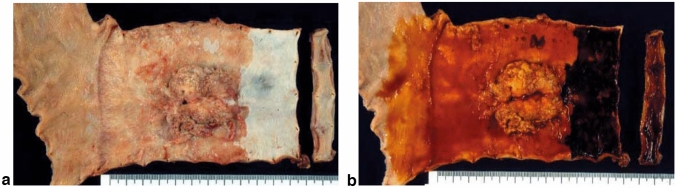
**a** Adenocarcinoma in Barrett’s esophagus: Type 1. A section of columnar epithelium measuring 85 mm in length and continuously extending to the esophagus is regarded as a long-segment of Barrett’s esophagus. A protruding tumor (Type 1) is visible within the section of Barrett’s esophagus. **b** Iodine-stained specimen shown in (**a**). Iodine staining clearly reveals the area of Barrett’s mucosa with scattered iodine-stained squamous islands. The depth of tumor invasion is pT1b

**Fig. 31:**
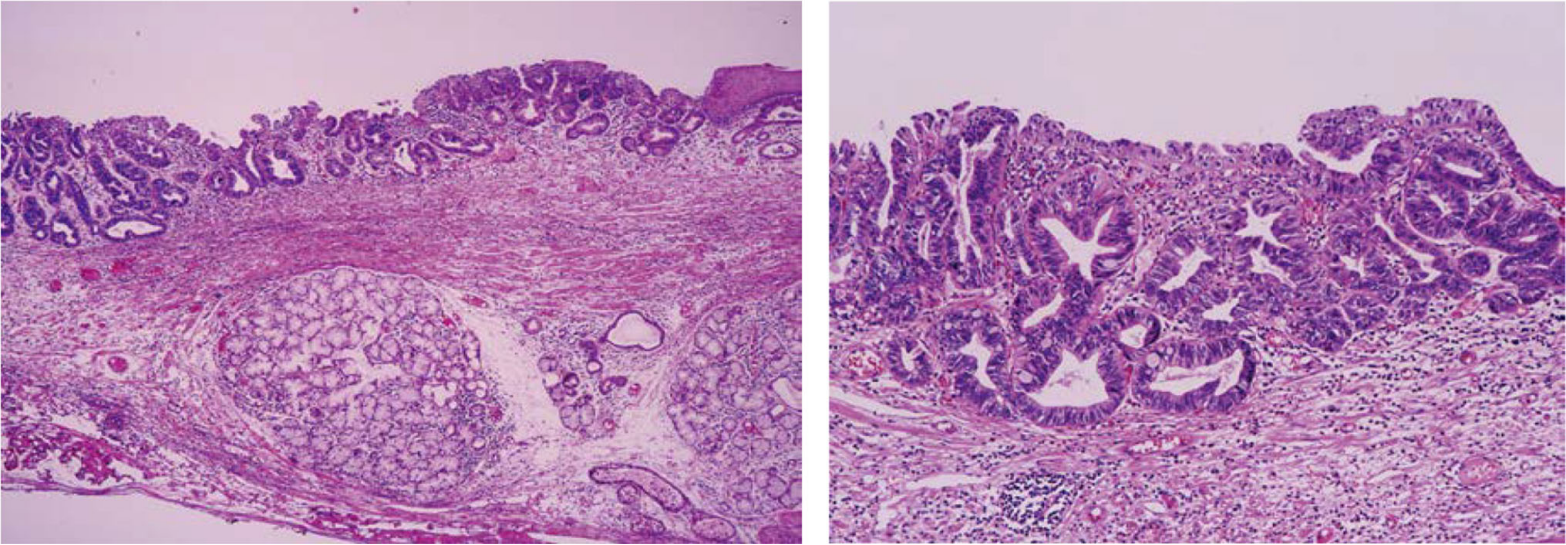
Adenocarcinoma in Barrett’s esophagus. Barrett’s esophagus, with esophageal glands proper in the submucosal layer covered with a well-differentiated adenocarcinoma. The tumor invaded beyond the superficial muscularis mucosa but did not reach the original (deep) muscularis mucosa. Therefore, the depth of tumor invasion should be assessed in pT1a-LPM

### 8.4. Esophagogastric junction cancer (EGJ cancer)

#### 8.4.1. Definition

**Fig. 32:**
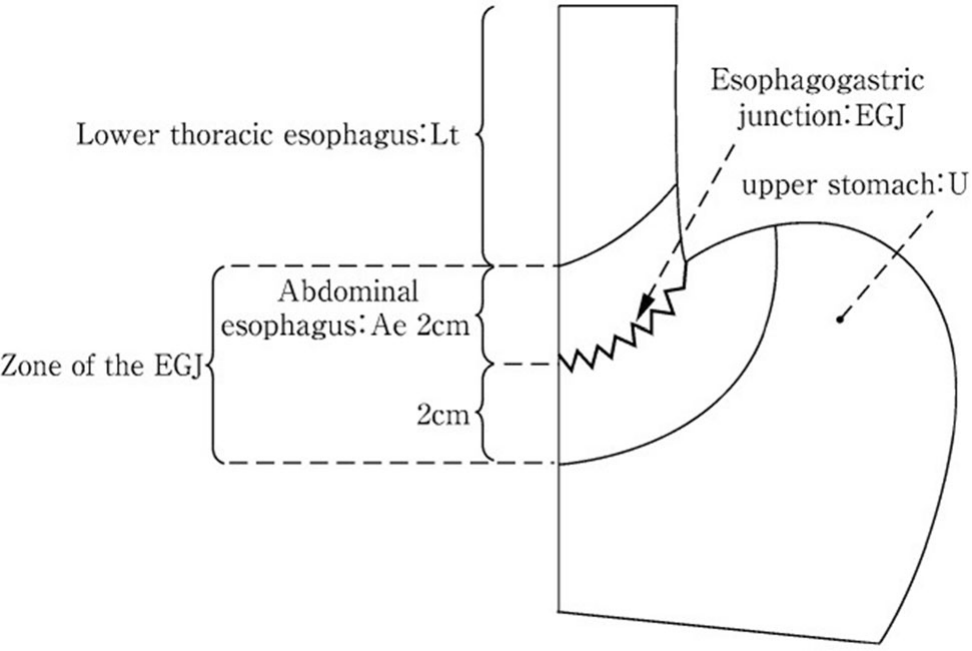
Definition and description of esophagogastric junction according to Nishi’s classification

The area between 2 cm above the esophagogastric junction and 2 cm below the esophagogastric junction is defined as “Zone of esophagogastric junction, Jz,” and a tumor of which center is located in Jz, irrespective of histology and with or without esophageal invasion, is called EGJ cancer. This definition is same as “Nishi’s classification” [10] (Fig. 32).

Until the previous Japanese Classification of Esophageal Cancer [7, 11], EGJ cancers had been used for both Nishi’s classification of cancer and the Siewert classification of Type II adenocarcinoma (adenocarcinoma with a tumor center located within 1 cm distal and 2 cm proximal to the EGJ). In this edition, EGJ cancer is recorded based only on Nishi’s classification. In addition, EGJ cancer is sometimes called “Junctional cancer.”

EGJ cancers are classified into three types according to their histology: adenocarcinoma, squamous cell carcinoma (SCC), and others (OTH), such as Jz (AC), Jz (SCC), and Jz (OTH).

The following descriptions should be recorded.

#### 8.4.2. Staging

The staging of EGJ cancer depends on histology. For adenocarcinoma, staging from the Japanese classification of gastric cancer should be used [1, 9]. For squamous cell carcinoma, the staging based on the classification of thoracic esophageal cancers in this edition (refer to 5.1 in Part I) should be used. However, the regional lymph nodes and extent of lymph-node dissection are determined as previously described (3.2 Regional lymph nodes in Part I) (5.3.3 Definition of the extent of lymph-node dissection (D) in Part I), irrespective of histology.

#### 8.4.3. Tumor location

**Fig. 33:**
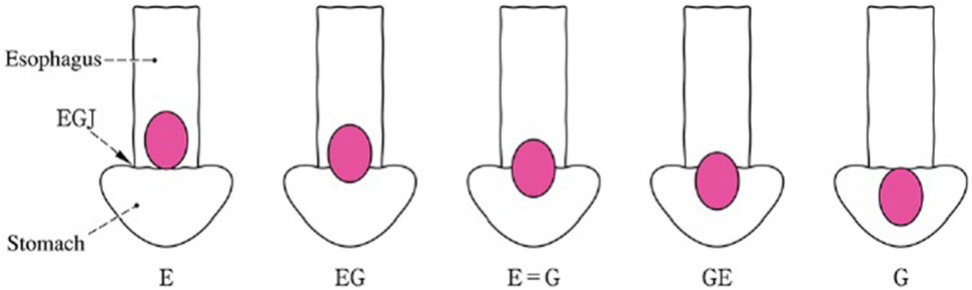
Subclassification and methods of description of cancer at the esophagogastric junction

The proximal and distal areas from the EGJ are described as “E” and “G,” respectively. As shown in Fig. 33, the terms “E,” “EG,” “E = G”, “GE,” and “G” can be used depending on the tumor location.

The distance between the tumor center (deepest site) and the EGJ is recorded as follows: minus, when the tumor center was proximal to the EGJ; plus, when the tumor center was distal to the EGJ.

#### 8.4.4. Esophageal invasion (EI)

The distance between the proximal end of the tumor and the EGJ must be recorded as the esophageal invasion (EI) ^*Note 1*^.

#### 8.4.5. Gastric invasion (GI)

The distance between the distal end of the tumor and the EGJ must be recorded as gastric invasion (GI) ^*Note 1*^.

#### 8.4.6. Barrett’s esophagus (BE)

According to 8.2 Barrett’s esophagus, its presence or absence should be recorded.

#### 8.4.7. Hiatal hernia (HH)

In case with clinically obvious hiatal hernia, its presence should be recorded.

e.g., Jz (SCC), T2N2M0, EG − 1 cm, EI:3 cm, GI:1 cm, BE (+), HH (+).

e.g., Jz (AC), T3N1M0, GE + 1 cm, EI: 2 cm, GI: 4 cm, BE (–), HH (–).

*Note 1:* Even in patients with obscure EGJ because of circumferential tumors or something else, the site of the EGJ should be estimated, and the tumor center, esophageal and gastric invasion should be described as much as possible.

*Note 2:* In some clinical databases, tumors are classified as esophageal or gastric cancers according to the tumor center. In these databases, if the tumor center is located at the EGJ (0 cm from the EGJ), adenocarcinoma should be recorded as gastric cancer and squamous cell carcinoma as esophageal cancer.

## 9 Treatment

### 9.1 Endoscopic treatment

#### 9.1.1 Endoscopic resection (ER)

9.1.1.1 Endoscopic mucosal resection (EMR)

9.1.1.2 Endoscopic submucosal dissection (ESD)

#### 9.1.2 Other endoscopic treatment

9.1.2.1 Argon plasma coagulation (APC)

9.1.2.2 Laser therapy (Laser)

9.1.2.3 Photodynamic therapy (PDT)

9.1.2.4. Microwave coagulation therapy (MCT)

9.1.2.5 Others

### 9.2 Surgical treatments

#### 9.2.1 Resection and reconstruction procedure

##### 9.2.1.1 Staged operation

- One-stage operation
- Staged operation

##### 9.2.1.2 Approaches for tumor resection

- Endoscopic
- Thoracoscopic and thoracoscopy-assisted
- Mediastinoscopic
- Laparoscopic and laparoscopy-assisted
- Robot-assisted
- Transcervical
- Thoracotomy
  Right
  Left
- Laparotomy
- Thoracoabdominal incision
  Right
  Left
- Transhiatal
- Sternotomy

*Note:* When several approaches are adopted, only the primary approach is described.

##### 9.2.1.3 Positions

- Left lateral decubitus position
- Prone position
- Supine position

##### 9.2.1.4 Extent of resection

- Total esophagectomy: Cervical, thoracic, and abdominal esophagus are resected regardless of whether laryngectomy is performed.
- Cervical esophagectomy: Cervical esophagus, with or without part of the upper esophagus, is resected regardless of whether laryngectomy or sternotomy is performed.
- Subtotal esophagectomy: Almost all of the thoracic esophagus is resected.
- Middle and lower esophagectomy: Middle and lower esophagi (including the esophagogastric junction zone) are resected ^*Note 1*^.
- Lower esophagectomy: Lower esophagus (including the esophagogastric junction) is resected ^*Note 1*^.
- Partial esophagectomy: Resection of full-thickness partial esophagus.^*Note 2*^
- Mucosal resection: Resection of mucosal and submucosal layers
- Others.

*Note 1:* (Middle and) lower esophagectomy with proximal gastrectomy is included in (middle and) lower esophagectomy.

*Note 2:* In a partial esophagectomy, the location of the resected esophagus should be described.

##### 9.2.1.5 Combined resection

The organ(s) resected together due to cancer invasion should be described.

*Note:* Total or partial resection of the organ(s) should be described.

##### 9.2.1.6 Reconstruction

**9.2.1.6.1 Routes**

- Antethoracic (subcutaneous)
- Retrosternal
- Posterior mediastinal

**9.2.1.6.2 Sites of anastomosis**

- Neck
- Antethoracic (subcutaneous)
- Thoracic cavity (proximal, distal) ^*Note*^
- Lower mediastinum.

*Note:* The border between the proximal and distal sites of the anastomosis is at the upper level of the aortic arch.

**9.2.1.6.3. Organs for substitution**

- Stomach
  Whole stomach
  Gastric tube
- Jejunum
- Colon
  Left colon
  Right colon
  Ileum and colon
- Skin and muscle^*Note 1*^
  Skin flap
  Local skin flap
  Musculocutaneous flap

*Note 1:* The name of the skin or muscle flap should be described.

E.g.: free forearm skin flap, right pectoralis major musculocutaneous flap

*Note 2:* In case of reconstruction not using stomach, pedicle or free reconstruction should be recorded.

#### 9.2.2 Conservative/palliative procedures

##### 9.2.2.1 Stoma

- Pharyngostomy
- Esophagostomy
- Gastrostomy
- Jejunostomy

**9.2.2.2 Bypass**

**9.2.2.3 Exploratory thoracotomy, exploratory laparotomy, staging thoracoscopy, and staging laparoscopy**

### 9.3 Stenting

#### 9.3.1 Esophageal stent

Type of stent, covered or uncovered, anti-reflux valve (yes, no), diameter, and length.

#### 9.3.2. Tracheobronchial stent

Type of stent.

#### 9.3.3. Aortic stent

Type of stent.

### 9.4 Balloon dilation

Balloon type, size, and atmospheric pressure.

### 9.5 Common issues for radiotherapy and chemotherapy

#### 9.5.1 Aim of treatment

Definitive, palliative, neoadjuvant, adjuvant, addictive, recurrent disease, and others.

*Note:* “Adjuvant” means a treatment after R0 resection and “additive” means a treatment after R1/R2 resection.

### 9.6 Radiotherapy (RT)

#### 9.6.1 Clinical target volume (CTV)

Primary lesion, entire esophagus, lymph-node metastasis, lymph-node area for prophylaxis (supraclavicular, mediastinal, and abdominal), and distant organ metastasis.

#### 9.6.2 Methods of radiotherapy

External beam radiation therapy, external beam radiation therapy plus intracavitary radiation therapy, and intracavitary radiation therapy alone.

#### 9.6.3 External beam radiotherapy

X-ray, proton, carbon, gamma-ray, and electron.

##### 9.6.3.1 Planning methods

X-ray simulator, and three dimensional.

##### 9.6.3.2 Field setting

Opposing, anterior oblique, three directions, four fields or more, rotation, and intensity-modulated radiotherapy (IMRT).

##### 9.6.3.3 Reference points

CTV center, field center, and others.

##### 9.6.3.4 Dose calculation

Non-homogeneity correction and algorithm.

##### 9.6.3.5 Dose fractionation of external beam radiotherapy

Dose per fraction, number of fractions per week, total dose, and overall treatment duration.

#### 9.6.4 Intraluminal irradiation

Low-dose rate (226Ra) and high-dose rate (192Ir, 60Co, and 137Cs).

##### 9.6.4.1 Reference points

Mucosal surface, how many_mm below mucosa.

##### 9.6.4.2 Dose fractionation of intraluminal irradiation

Dose per fraction, number of fractions per week, total dose, and overall treatment duration.

#### 9.6.5 Completion of treatment

Complete, complete with break, and incomplete.

#### 9.6.6 Chemoradiotherapy (CRT)

With or without chemotherapy.

*Note 1:* Drug name for chemotherapy should be described.

*Note 2:* The time of chemotherapy should be divided into “concurrent” or “sequential.” In patients who undergoes (chemo) radiotherapy after chemotherapy, the preceding chemotherapy should be classified as “induction” based on 9.7.1.

### 9.7 Chemotherapy (CT), immuno-oncology drug (IO)

#### 9.7.1 Aim of treatment

Induction, neoadjuvant, adjuvant, and palliative.

#### 9.7.2 Agent

Name of agent (generic name should be recorded).

#### 9.7.3 Administration route

Intravenous, oral, transarterial, local injection (including abdominal and chest cavities), and others.

#### 9.7.4 Administration procedure

With or without radiation, bolus, continuous, and others.

#### 9.7.5 Administration dose

The dose is recorded as per body surface area (m^2^) or per body (body).

#### 9.7.6 Administration schedule

Course duration, course interval, and upper limit in number of courses.

#### 9.7.7 Duration of administration

Initial date of administration, last date of administration, and total number of courses.

#### 9.7.8 Total administration dose

The total administration dose of each agent is calculated per body surface area or per body.

#### 9.7.9 Adverse events

Adverse events are recorded according to the “Common Terminology Criteria for Adverse Events v5.0, (Japanese version) JCOG or JSCO edition” [12].

### 9.8 Multi-modality treatment

#### 9.8.1 Surgery in multi-modality treatment

- Planned surgery: Surgery planned after chemotherapy, radiotherapy, or chemoradiotherapy as neoadjuvant treatment for a patient with a resectable tumor at the initial visit.
- Salvage surgery: surgery for residual or recurrent tumors after definitive (chemo) radiotherapy. The total radiation dose is limited to ≥ 50 Gy.
- Conversion surgery: Surgery for a tumor that was unresectable at the initial visit and resectable after treatment.

The types of surgery, including esophagectomy, lymph-node removal (dissection), and endoscopic treatment, should be described.

#### 9.8.2 Endoscopic treatment in multi-modality treatment

- Salvage endoscopic treatment: Endoscopic treatment for a residual or recurrent tumor after definitive (chemo) radiotherapy. Total radiation dose is limited to ≥ 50 Gy.

Type of endoscopic treatment should be described (for example, APC, EMR, ESD, and PDT).

**9.9 Hyperthermia (HT)**

## 10. Treatment results

The following data should be recorded to allow precise statistical analysis of a comprehensive registry of esophageal cancer.

### 10.1 Long-term outcome

The following items should be recorded for survival analysis.

#### 10.1.1 Alive or dead

Alive: The date of the most recent follow-up

Death: The date of death

Unknown: The date of the most recent follow-up

Cause of death:

Treatment-related deaths are as follows:

Surgical treatment, chemotherapy, radiotherapy, or other therapies

Esophageal cancer

Another cancer (primary cancer-related deaths should be recorded)

Another disease (the name(s) of the disease(s) should be recorded)

Accident (including suicide)

Unknown cause(s) (this category should be regarded as death due to esophageal cancer if recurrence is confirmed)

#### 10.1.2 Recurrence

Yes or no

Date of recurrence

Pattern and recurrence site: Each recurrence should be recorded chronologically.

Local recurrence:

Primary lesion (esophagus) ^*Note 1*^

In the mediastinum adjacent to the primary lesion

At the anastomotic site or in the esophageal stump

In the regional lymph nodes

Others, including intramural metastasis in the esophagus or stomach

Distant metastasis: ^*Note 2*^

Lymphogenous recurrence (distant lymph node)

Hematogenous recurrence (distant organ(s))

Disseminated recurrence (pleura, peritoneum, and pericardium)

Unknown

*Note 1:* Recurrence at the same location as the primary lesion in the esophagus can occur after esophagus-preserving treatments, including endoscopic mucosal resection, chemotherapy, and radiotherapy.

*Note 2:* Recurrent organs (s) are indicated by abbreviations in the TNM classification.

Abbrev iations: Liver, HEP; Lung, PUL; Peritoneum, PER; Lymph node, LYM; Bone, OSS; Brain, BRA; Kidney, REN; Adrenal gland, ADR; Skin, SKI; Other, OTH

**Response evaluation criteria in radiotherapy and chemotherapy for esophageal cancer**

**Introduction**

In this edition, we add new response evaluation criteria using CT and endoscopy for primary lesions, considering the growing importance of neoadjuvant treatment. We create new evaluation criteria by CT, because responses of the primary lesion to neoadjuvant treatment significantly affect survival, and we need a more standardized evaluation using CT. Therefore, based on findings from our retrospective multi-institutional study, we regard the primary lesion as measurable under certain circumstances. Evaluation by endoscopy should be revised, because small tumors cannot be measured by CT. In this edition, we create a new sub-classification of remarkable response (RR) for the primary lesion by endoscopy in non-CR/non-PD patients. This new sub-classification of RR is confirmed to be associated with histological response and survival and is expected to be clinically important. Furthermore, we add new endoscopic evaluation criteria for local recurrence after primary lesion CR. Although many studies have reported the significance of evaluating responses using PET–CT, we decide not to adopt PET–CT as a formal modality of evaluation because its outcomes are affected by equipment, measurement conditions, or the patient’s status. Furthermore, we do not know which index is the most appropriate (maximum standardized uptake value (SUVmax), SUV normalized by lean body mass (SUL), metabolic tumor volume (MTV), or total lesion glycolysis (TLG)) for response evaluation using PET–CT. In this edition, we refer to PET–CT for the supplementary findings. In the near future, positron emission tomography/computed tomography or other modalities should be adopted.

## 11. Definitions

This is response evaluation criteria for patients with esophageal cancer treated with chemotherapy, radiotherapy, or chemoradiotherapy.

### 11.1 Classification of tumor lesions and lymph nodes

#### 11.1.1 Measurable lesions

Tumor lesions (except for primary lesion and lymph node):

Lesions that could be accurately measured in at least one dimension (the greatest dimension should be recorded) and met the following criteria:

≥10 mm by CT scan (slice of CT scan with ≤5 mm)

≥ 10 mm with a caliper by clinical exam (lesions which cannot be accurately measured with a caliper should be recorded as “non-measurable.”

≥ 20 mm by chest X-ray.

Malignant lymph nodes

To be considered pathologically enlarged and measurable, a lymph node must be ≥ 15 mm in short axis when assessed by CT scan (CT scan slice thickness recommended to be less than 5 mm). Only the short axis is measured at baseline and follow-up.

Primary esophageal lesion

In a previous series on the Japanese Classification of Esophageal Cancer, esophageal primary lesions were considered non-measurable. In this current edition, it is defined as measurable in case with ≥ 20 mm in long axis by CT scan. Lesions < 20 mm along the long axis are defined as non-measurable, and response evaluation should be performed using endoscopy (including biopsies).

#### 11.1.2. Non-measurable lesions

All other lesions, including small lesions (longest diameter < 10 mm or pathological lymph nodes with ≥ 10 mm to < 15 mm short axis) and truly non-measurable lesions.

#### 11.1.3 Target lesions

When more than one measurable lesion is present at baseline, all lesions up to a maximum of five lesions (and a maximum of two lesions per organ) representative of all involved organs should be identified as target lesions and recorded and measured at baseline (the instances where patients have only one or two organ sites involved, a maximum of two and four lesions, respectively, should be recorded).

#### 11.1.4 Non-target lesions

Any other lesions or disease sites should be identified as non-target lesions.

## 12. Response evaluation criteria for target lesions

1. When measurable lesions or non-measurable lesions are detected other than primary esophageal lesions, they should be evaluated according to the RECIST criteria [13].
2. The same methods as used in baseline evaluation should be adopted.
3. All measurements should be recorded in metric notation.
4. All baseline evaluations should be performed before treatment, as close as possible to the beginning of treatment. They should be performed in the last 4 weeks before the treatment begins.
5. When more than one measurable lesion is present at baseline, all lesions up to a maximum of five (a maximum of two lesions per organ), representative of all involved organs, should be identified as target lesions and must be recorded and measured at baseline (when patients have only one or two organ sites involved, a maximum of two and four lesions, respectively, will be recorded). The sum of the diameters (longest for non-nodal lesions and short axis for nodal lesions) for all target lesions was calculated and reported as the baseline sum of the diameters. If lymph nodes are to be included in the sum, only the short axis should be added, as noted above. The baseline sum diameters should be used as a reference to further characterize any objective tumor regression in the measurable dimension of the disease.

### 12.1 Complete response (CR)

Disappearance of all target lesions. Any pathological lymph node (target or non-target) must have a reduction in the short axis to < 10 mm.

### 12.2 Partial response (PR)

At least a 30% decrease in the sum of the diameters of target lesions should be observed, taking the baseline sum diameters as a reference.

### 12.3 Progressive disease (PD)

At least a 20% increase in the sum of diameters of target lesions, taking as reference the smallest sum on the study (including the baseline sum if it is the smallest on the study). In addition to a relative increase of 20%, the sum had to demonstrate an absolute increase of at least 5 mm.

### 12.4 Stable disease (SD)

Neither sufficient shrinkage to qualify for PR nor a sufficient increase to qualify for PD, taking as reference the smallest sum of diameters during the study.

## 13 Response evaluation criteria for non-target lesions

### 13.1 Complete response (CR)

The disappearance of all non-target lesions and normalization of tumor marker levels. All lymph nodes were non-pathological in size (<10 mm short axis). The CR of primary esophageal lesions should be determined based on endoscopic CR (refer to 15.1 and 22.1).

### 13.2 Non-CR/non-PD

Persistence of one or more non-target lesion(s) and/or maintenance of tumor marker levels above normal limits. Non-CR/non-PD of primary esophageal lesions should be determined based on the PR/SD on CT (refer to 4.1), and endoscopic non-CR/non-PD (refer to 15.2 and 22.2).

### 13.3 Progressive disease (PD)

Unequivocal progression of existing non-target lesions or appearance of one or more new lesions. PD of primary esophageal lesions should be determined based on PD on CT (refer to 4.1), and endoscopy (refer to 15.3 and 22.4).

## 14. Response evaluation criteria of CT/PETCT for primary tumors (refer to 21)

### 14.1 Response evaluation criteria of CT

#### 14.1.1 Evaluation using short diameter of primary tumor (refer to 21.1) [14]

Method

For the primary lesion with ≥ 20 mm of long diameter in CT (axial), its short diameter is measured on the plane with the longest diameter of the primary lesion.

Reduction rate of primary lesion (∆tumor short diameter) = [(short diameter before treatment − short diameter after treatment)/(short diameter before treatment)] × 100%

Response evaluation criteria of the short diameter of the primary lesions by CT

PR: ∆tumor short diameter ≥30%

SD: ∆tumor short diameter ≥− 20%, <30%

PD: ∆tumor short diameter <− 20% (at least 20% increase in short diameter).

#### 14.1.2 Evaluation using esophageal cross-section area (refer to 21.1) [15]

If it is difficult to evaluate the primary lesion using the method 14.1.1 (described above), the method using reduction rate of cross-section area of the esophagus can be chosen as an alternative for the primary lesion with ≥ 20 mm of long diameter in CT (axial) before treatment. This method is not applicable to esophagogastric junction cancer (Jz).

Method

Cross-sectional area is calculated by multiplying the long esophageal diameter by the short esophageal diameter in the plane with the longest diameter of the primary lesion.

Reduction rate of cross-section area (∆ esophageal cross-section area) = [(cross-section area before treatment − cross-sectional area after treatment) / (cross-sectional area before treatment)] × 100%

*Note:* If the long diameter of the tumor becomes obscure on CT after treatment, cross-sectional area should be calculated on the same plane as the plane with the longest axis before treatment.

Response evaluation criteria of cross-section area of the esophagus by CT

PR: ∆ esophageal cross-section area ≥40%

SD: ∆ esophageal cross-section area ≥− 20%, <40%

PD: ∆ esophageal cross-section area <− 20% (at least a 20% increase in cross-sectional area).

### 14.2. Response evaluation criteria of PET–CT (refer to 21.2) [16]

Method

In patients with a primary tumor with cT2-4 and SUVmax ≥ 3 before treatment, the reduction rate of SUVmax (∆SUVmax) is calculated.

Reduction rate of SUVmax (∆SUVmax) = 

[(SUVmax before treatment − SUVmax after treatment) / (SUVmax before treatment)] × 100%.

Response evaluation criteria of SUVmax of the primary lesions by PET- CT

PR: ∆SUVmax ≥50%

SD: ∆SUVmax ≥− 10% and <50%

PD: ∆SUVmax <− 10%.

*Note 1:* PET–CT examinations before and after treatment should be performed using the same machine.

*Note 2:* We should consider that SUVmax can be affected by blood sugar level and inflammation (reflux esophagitis, radiation pneumonitis, etc.).

*Note 3:* If the sites at which SUVmax was measured before and after treatment are different, the reduction rate of SUVmax can be evaluated if these sites are in the same primary lesions.

*Note 4:* CR should not be defined using CT or PET/CT. In cases achieving PR on CT/PET–CT and CR on endoscopy (described below), the primary lesion is defined as overall CR.

## 15. Response evaluation criteria for primary lesion using endoscopy (refer to 22)

CR can be defined only by endoscopy irrespective of the tumor’s depth or size, then this classification defines CR using endoscopy. However, response evaluation other than CR should be performed using CT. In cases where CT evaluation is difficult, such as primary lesions < 20 mm in size, response evaluation using endoscopy should be prioritized over CT evaluation.

### 15.1 Complete response of primary lesion (primary lesion CR)

When conditions (1)–(5) are satisfied, the response is judged as CR.

1. Disappearance of any endoscopic findings suggesting the presence of a tumor.^*Note 1*^
  Eroding changes in the mucosa
  Irregular surface
  Ulcerative lesions
  Distinctly protruded changes (including protrusions suggestive of a submucosal tumor).
2. Entire esophagus can be observed using endoscopy.
3. No endoscopic findings of active esophagitis, such as flat erosive findings or white coating.
4. Histologically negative for carcinoma in the endoscopic biopsy specimens from the area of the primary lesion.^*Note 2*^
5. Even if the primary lesion is ≥ 20 mm of long axis in CT, 1 to 4 criteria must be met to define CR.

*Note 1:* The findings noted below should not be considered possible tumor lesions. The lesions were then judged to be in CR.

- Scars
- Stenosis
- Iodine-unstained areas or poorly stained areas
- Biopsy-negative granulomatous small elevated lesion

*Note 2:* If cancer cells are recognized in biopsy specimens, the lesion should be defined as non-CR/non-PD (ref. to 5.2).

### 15.2 Non-CR/non-PD (progressive disease) of primary lesion (primary lesion non-CR/non-PD)

Response of primary lesion is judged as non-CR/non-PD if it does not meet the criteria for CR (refer to 15.1) or PD (refer to 15.3). However, when the primary lesion shrinks remarkably to a long diameter of < 20 mm on CT, and the entire esophagus can be observed using endoscopy, the following evaluation should be noted in this classification.

#### 15.2.1 Remarkable response of primary lesion (primary lesion RR)

When tumor volume remarkably shrinks to nearly T1 depth after treatment and does not meet the criteria of CR (refer to 15.1), then the primary lesion is defined as the primary lesion RR and recorded as non-CR/non-PD (RR). This evaluation does not depend on the diagnosis of the biopsy specimens.

*Note 1:* Primary lesion of cT1 (T1a or T1b) before treatment should be determined as the primary lesion RR only when the reduction rate of the primary lesion is ≥ 90% endoscopically.

### 15.3 Progressive disease of primary lesion (primary lesion PD)

When a distinct tumor growth ^*Note*^ is found compared to that before treatment or the best response during treatment, it is defined as a progressive disease of primary lesion (primary lesion PD).

*Note:* Distinct tumor growth is expected to increase one and a half time or more.

### 15.4 Local recurrence of primary lesion during follow-up (primary lesion LR)

Local recurrence (LR) is determined when cancer cells are identified in biopsy specimens during follow-up after CR. In cases with some endoscopic findings at the previous primary lesion site, a biopsy should be performed if local recurrence is suspected. Even if no cancer cells are detected in biopsy specimens, close re-examination is required. Endoscopic findings of LR are as follows:

- Eroding changes in the mucosa
- Irregular surface
- Ulcerative lesions
- Distinctly protruded changes (including protrusions suggestive of a submucosal tumor)
- New iodine staining.

## 16. Overall response

All possible combinations of tumor responses in target and non-target lesions with or without the appearance of new lesions (Table 1).

**Table 1:** Overall response assessment for primary lesions, metastasis, and new lesions

Primary lesions		Metastasis		New lesions	Overall response
CT evaluation	Endoscopic evaluation (supplementary findings)	Target lesions	Non-target lesions		
PR	CR	CR	CR	No	CR
PR	Non-CR/Non-PD	CR or PR	CR or Non-CR/non-PD	No	PR
SD	Non-CR/Non-PD	CR or PR	CR or Non-CR/non-PD	No	SD ^Note 1^
Non-PD	Any	SD	Non-PD	No	SD
Any	Any	PD	Any	Yes or No	PD
PD	Any	Any	Any	Yes or No	PD
Any	Any	Any	PD	Yes or No	PD
Any	Any	Any	Any	Yes	PD

*Note 1:* When the primary lesion is SD with target lesions of CR or PR and non-target lesions (other than the primary lesion) of non-PD, the overall response is determined as SD (defined as PR in the RECIST criteria)

*Note 2:* When only non-target lesions are available without any target lesions (e.g., primary esophageal cancer without lymph-node metastasis), the overall response is determined only by those from the non-target lesions

## 17. Best overall response and confirmation

The best overall response is recorded from the start of the study until the end of the treatment, considering any requirement for confirmation. The patient’s best overall response assignment will depend on the findings of both target and non-target diseases, and will also take into consideration the appearance of new lesions. The best overall response is determined when the patient’s data became available (RESIST should be referred to) [13].

## 18. Criteria for histological response of chemotherapy and/or radiotherapy (Fig. 34) (refer to 23)

In cases of preoperative chemotherapy and/or radiation, dose of chemotherapy, radiation dose and method of administration, type, and time interval between preoperative therapy and surgical resection of tumor are described. In cases of preoperative treatment, all specimens in which primary tumor is macroscopically present should be examined histologically [1].

Grade 0: Ineffective

No recognizable cytological or histological therapeutic effect.

Grade 1: Slightly effective

Viable cancer cells (including those with eosinophilic cytoplasm with vacuolation and swollen nuclei) account for 1/3 or more of the tumor tissue; however, there is some evidence of degeneration of cancer tissue or cells.

Grade 1a: Viable cancer cells account for 2/3 or more tumor area.

Grade 1b: Viable cancer cells account for ≥1/3 but <2/3 of the tumor area.

Grade 2: Moderately effective

Viable cancer cells account for <1/3 of the tumor area, whereas other cancer cells are severely degenerated or necrotic.

Grade 3: Markedly effective

No viable cancer cells are evident.

*Note:* Definite re-proliferation of tumor cells in treated cancer lesions, after preoperative treatment, should be recorded as ‘‘re-proliferation (+)’’.

**Fig. 34:**
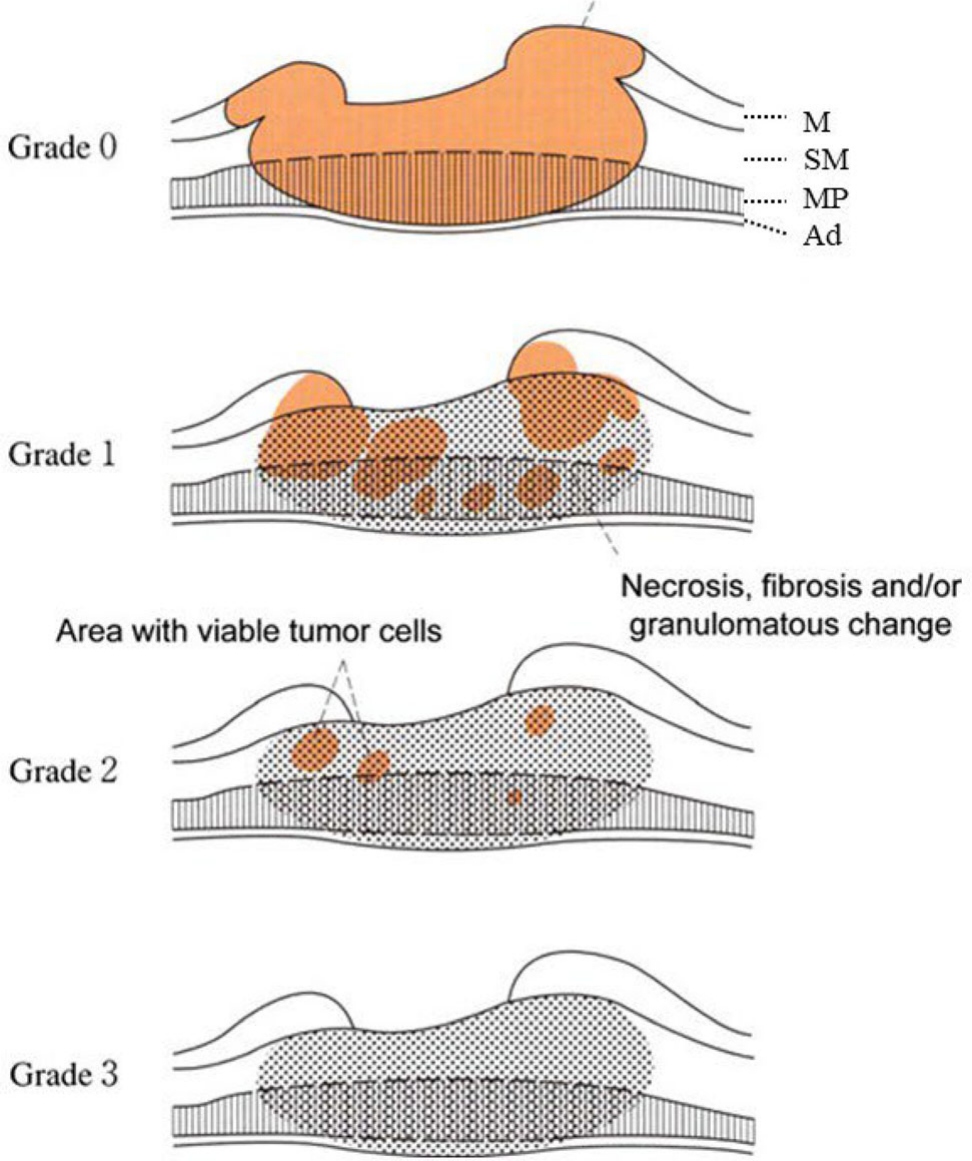
Histological efficacy of chemotherapy and/or radiotherapy. This figure is modified from ref. [1] (reproduced with permission of reproduced with permission of Japanese Gastric Cancer Association. Japanese Classification of Gastric Carcinoma–15.^th^ Edition)

**References**

## 19. Naming, number, range and boundary of regional lymph node of esophageal cancer (refer to 3.1 of Part I)

### 19.1. Cervical lymph nodes (Figs. 35, 36, 37)

No. 100: Superficial lymph nodes of the neck.

(Cervical lymph nodes except for deep cervical nodes, according to the General Rules for Clinical Studies on Head and Neck Cancer [17]).

No. 100spf: Superficial cervical lymph nodes: Lymph nodes located along the external and anterior jugular veins beneath the superficial cervical fascia.

No. 100sm: Submandibular lymph nodes: Lymph nodes located around the submandibular and parotid glands, anterior to the mylohyoid muscle.

No. 100tr: Cervical pretracheal lymph nodes: Lymph nodes located in the pretracheal fatty tissue, extending from the hyoid bone to the left brachiocephalic vein, including the prethyroidal and prelaryngeal lymph nodes.

No. 100ac: Accessory nerve lymph nodes: lymph nodes located along the accessory nerve(s) and anterior to the trapezius muscle.

No. 101: Cervical paraesophageal lymph nodes.

The lymph nodes are located around the cervical esophagus, including the recurrent laryngeal nerve and cervical paratracheal lymph nodes. The lateral boundary is located at the medial border of the carotid sheath. The left and right sides are distinguished.

No. 102: Deep cervical lymph nodes.

Lymph nodes located around the internal jugular vein and the common carotid artery.

No.102up: Upper deep cervical lymph nodes: lymph nodes located at the caudal border of the digastric muscle superior to the carotid artery bifurcation.

No. 102 mid: Middle deep cervical lymph nodes: Lymph nodes located from the carotid artery bifurcation to the lower border of the cricoid cartilage inferiorly.

No. 103: Peripharyngeal lymph nodes.

The lymph node was located medial to the carotid sheath and extended from the caudal border of the digastric muscle to the lower border of the cricoid cartilage. Postpharyngeal and parapharyngeal lymph nodes are included.

No. 104: Supraclavicular lymph nodes.

The lymph nodes, including the lower internal deep cervical lymph nodes, are located in the supraclavicular fossa and extend to the lower border of the cricoid cartilage. The medial boundary is located at the medial border of the carotid sheath. The left and right sides are distinguished.

**Fig. 35:**
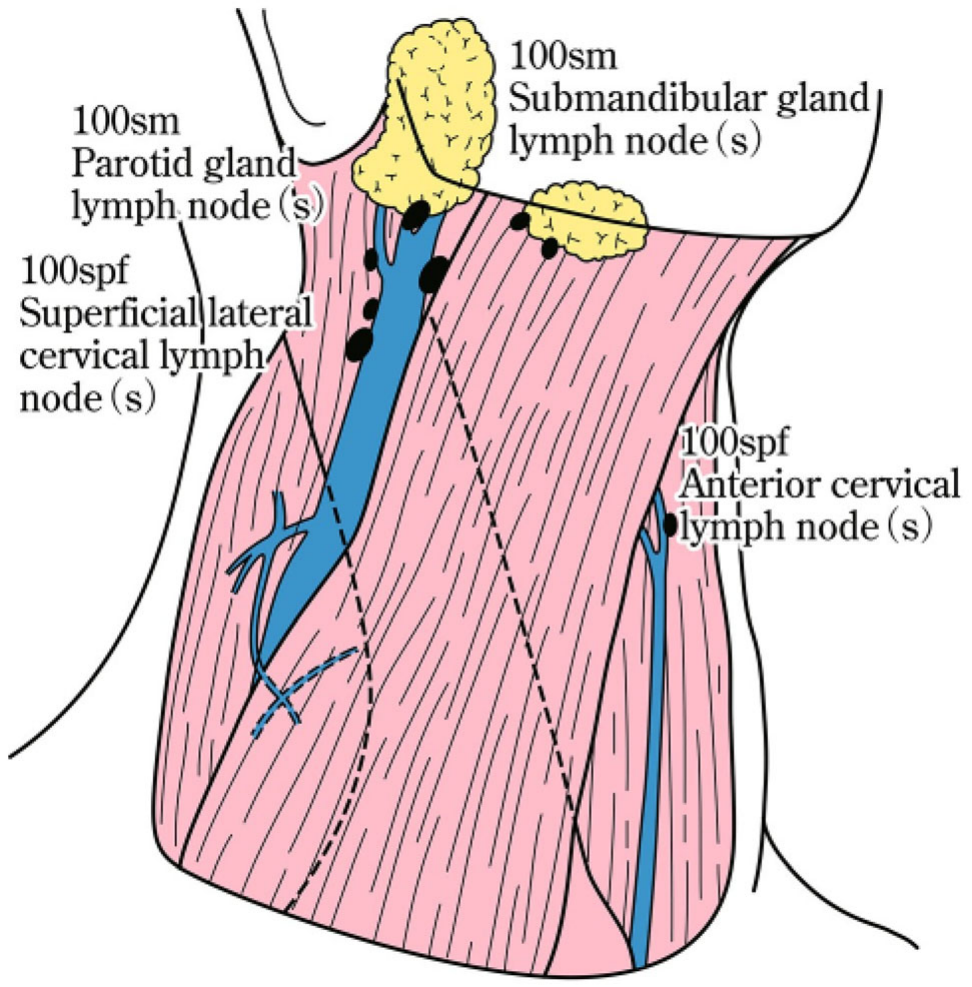
Superficial cervical lymph nodes

**Fig. 36:**
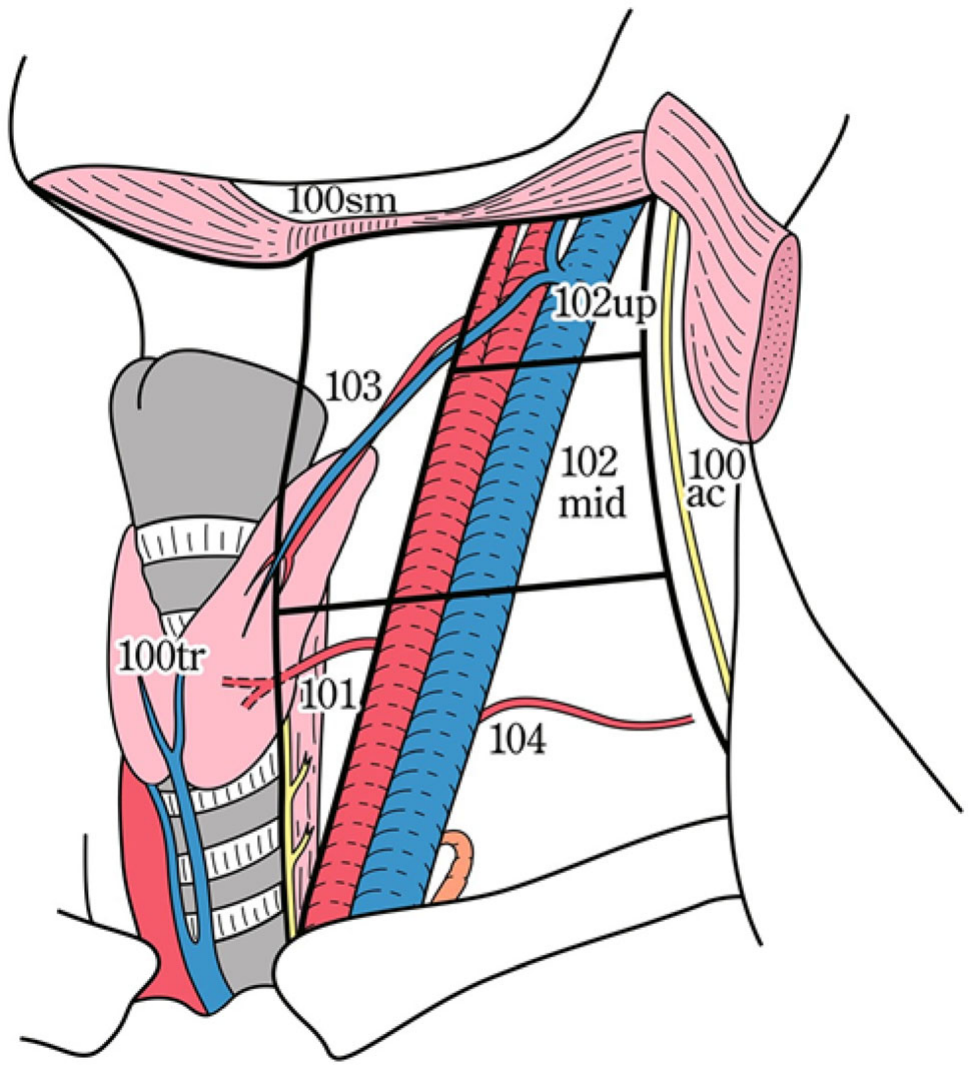
Deep cervical lymph nodes

**Fig. 37:**
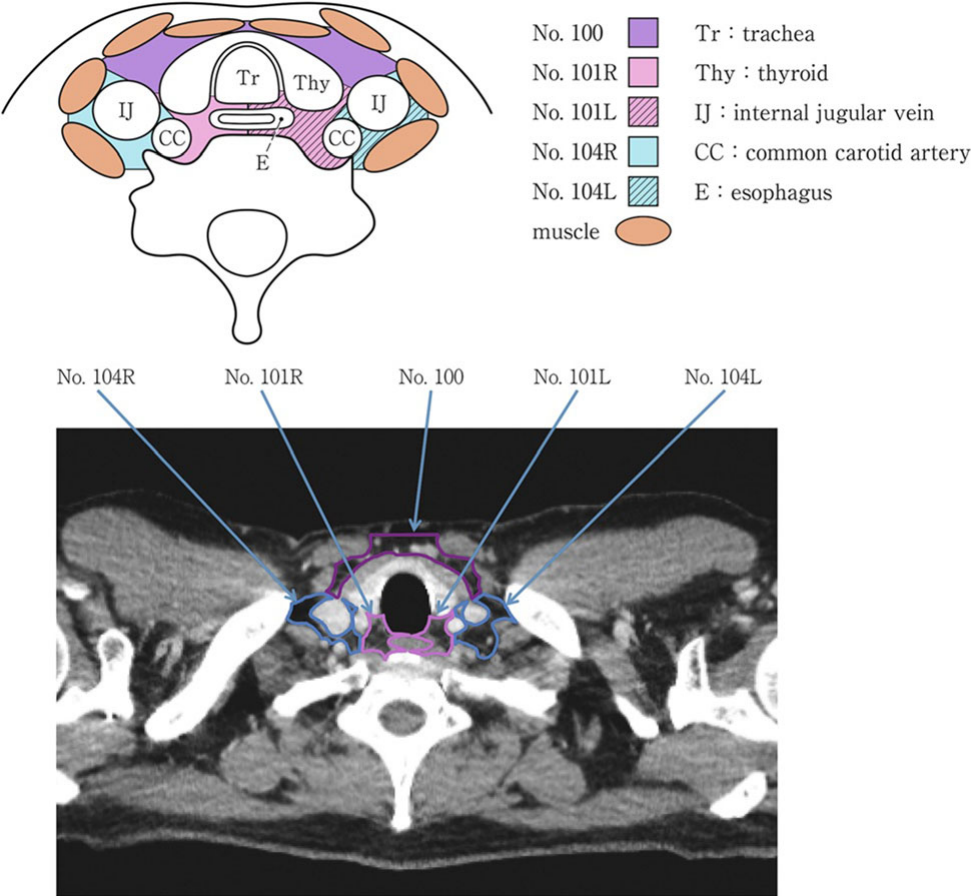
Cervical lymph nodes

### 19.2. Thoracic lymph nodes (Figs. 38, 39, 40, 41, 42, 43, 44, 45, 46)

No. 105: Upper thoracic paraesophageal lymph nodes.

The lymph nodes are located around the upper thoracic esophagus, posterior to the right vagal nerve on the right side. Lymph nodes located along the azygos vein arch and the right bronchial artery are included. The superior boundary is drawn from the cephalic border of the subclavian artery to the suprasternal notch.

No. 106: Thoracic paratracheal lymph nodes.

Lymph nodes are located along the anterior and lateral wall of the thoracic trachea.

No. 106rec: Recurrent nerve lymph nodes: lymph nodes located along the recurrent laryngeal nerves in the mediastinum. The superior boundary is drawn from the cephalic border of the subclavian arteries to the suprasternal notch, and the inferior boundary is drawn from the caudal border of the recurrent laryngeal nerve, curving upward on both sides.

No. 106recL: Left recurrent nerve lymph nodes

Lymph nodes located along the left recurrent laryngeal nerve

No. 106recR: Right recurrent nerve lymph nodes

Lymph nodes are located along the right recurrent laryngeal nerve.

No. 106pre: Pretracheal lymph nodes

Lymph nodes are located in front of the anterior wall of the thoracic trachea and anterior to the right vagal nerve.

No. 106tb: Tracheobronchial lymph nodes

Lymph nodes are located in the tracheobronchial angle.

No. 106tbL: Left tracheobronchial lymph nodes

The superior border is the inferior wall of the aortic arch, and the lymph nodes are located in the area surrounded by the medial wall of the aortic arch.

No. 106tbR: Right tracheobronchial lymph nodes

The superior border is the inferior wall of the azygos vein.

No. 107: Subcarinal lymph nodes.

Lymph nodes are located caudal to the carina of the trachea. The lateral boundaries are the extended lines of the lateral margins of the trachea.

No. 108: Middle thoracic paraesophageal lymph nodes.

Lymph nodes located around the middle thoracic esophagus.

No. 109: Main bronchus lymph nodes.

Lymph nodes in the caudal area of the main bronchus. The internal boundary is the border of the subcarinal lymph nodes, and the external boundary is the lung.

No. 110: Lower thoracic paraesophageal lymph nodes.

Lymph nodes are located around the lower thoracic esophagus.

No. 111: Supradiaphragmatic lymph nodes.

Lymph nodes are located in the area surrounded by the diaphragm, pericardium, and esophagus.

No. 112: Posterior mediastinal lymph nodes.

Lymph nodes located in the area surrounded by the descending aorta, inferior pulmonary vein, and pericardium. The lymph nodes are divided into subgroups.

No. 112aoA: Anterior thoracic para-aortic lymph nodes: lymph nodes located around the descending aorta and on the same side of the esophagus, including lymph nodes along the thoracic duct.

No. 112aoP: Posterior thoracic para-aortic lymph nodes: lymph nodes located around the descending aorta and on the opposite side of the esophagus.

No. 112pul: Pulmonary ligament lymph nodes

Lymph nodes located in the pulmonary ligament(s), including lymph nodes adjacent to the pericardium and the inferior pulmonary vein. The left and right sides are distinguished.

No. 113: Ligamentum arteriosum lymph nodes (Botallo’s lymph nodes).

Lymph nodes are located on the left side of the arterial ligament.

No. 114: Anterior mediastinal lymph nodes.

Lymph nodes are located anterior to the superior vena cava, including the lymph nodes of the brachiocephalic venous angle and those around the thymus gland.

**Fig. 38:**
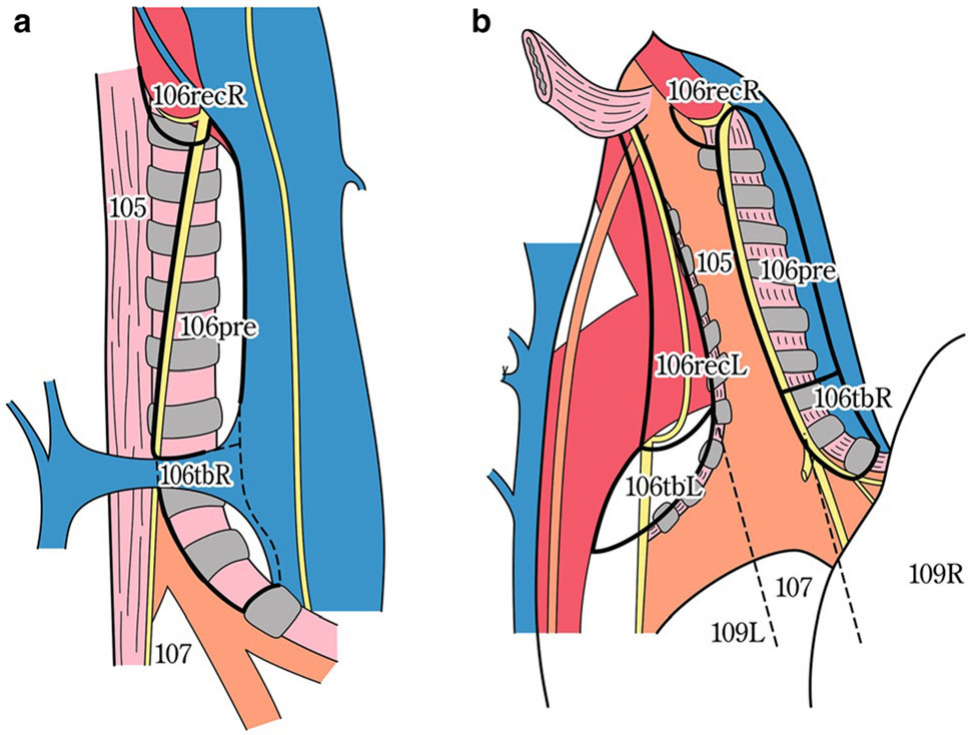
A and b Tracheobronchial lymph nodes (a: right view of the trachea; b: posterior view of the trachea)

**Fig. 39:**
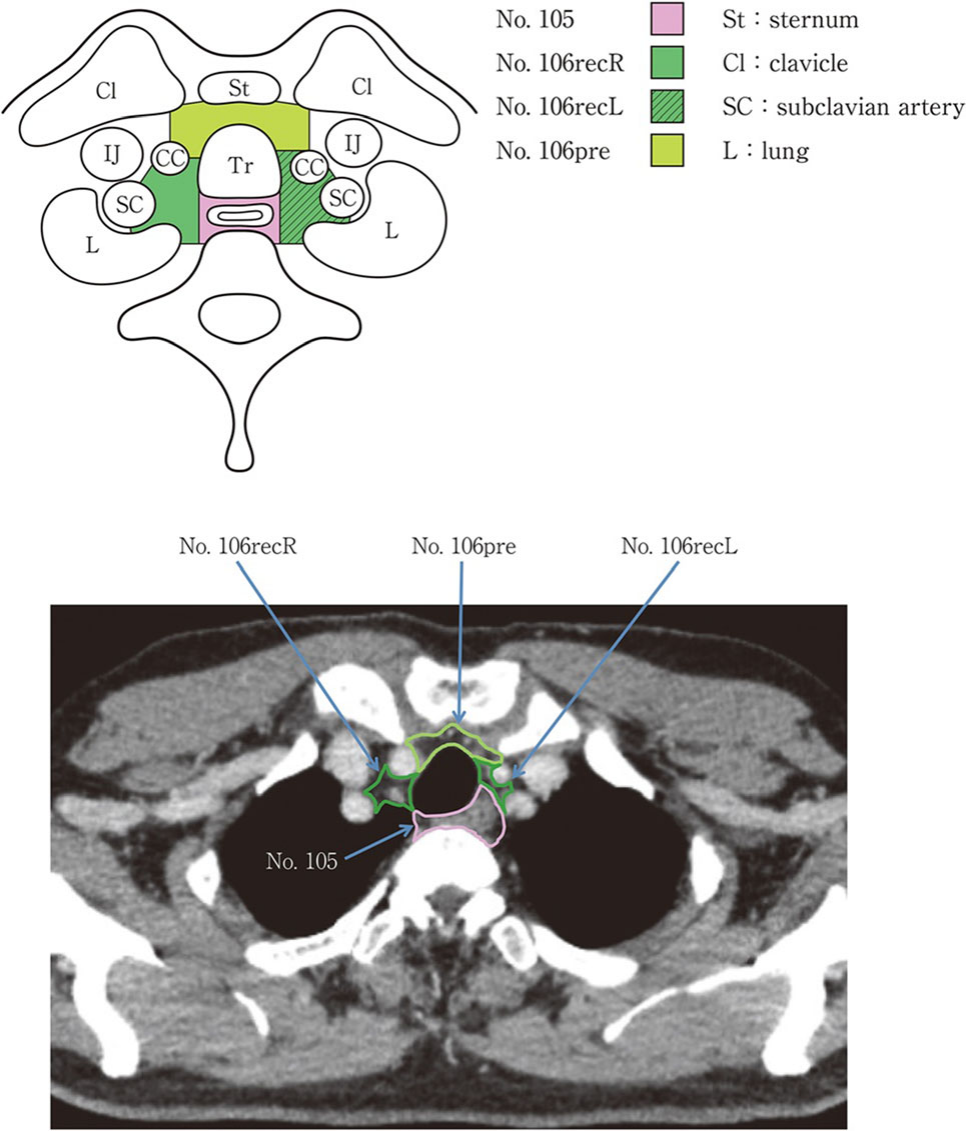
Highest part of the upper mediastinal lymph nodes (border between the neck and the thorax). Abbreviations: *Tr* trachea, *Thy* thyroid, *IJ* internal jugular vein, *CC* common carotid artery, *E* esophagus

**Fig. 40:**
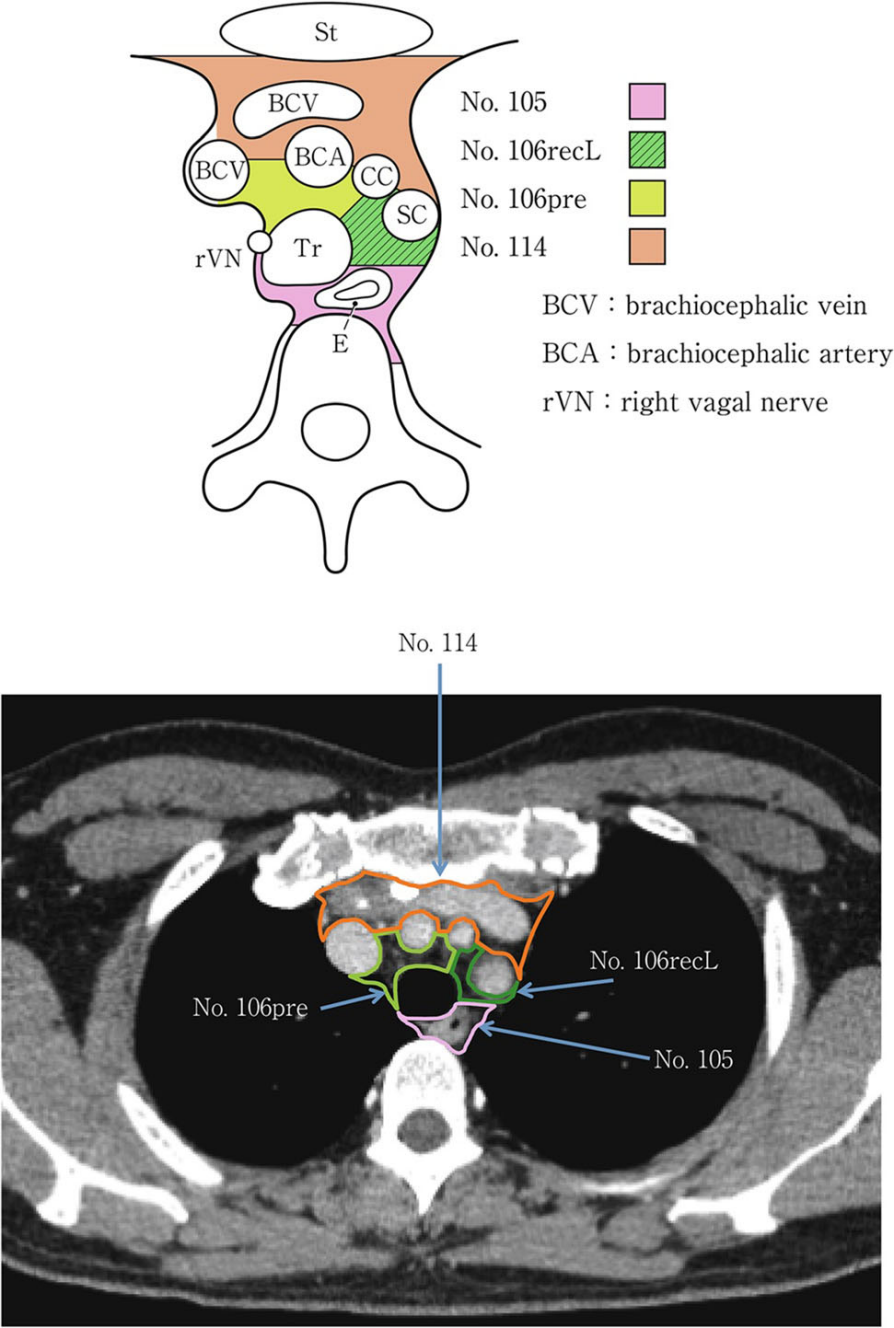
Upper mediastinal lymph nodes at the level above the aortic arch

**Fig. 41:**
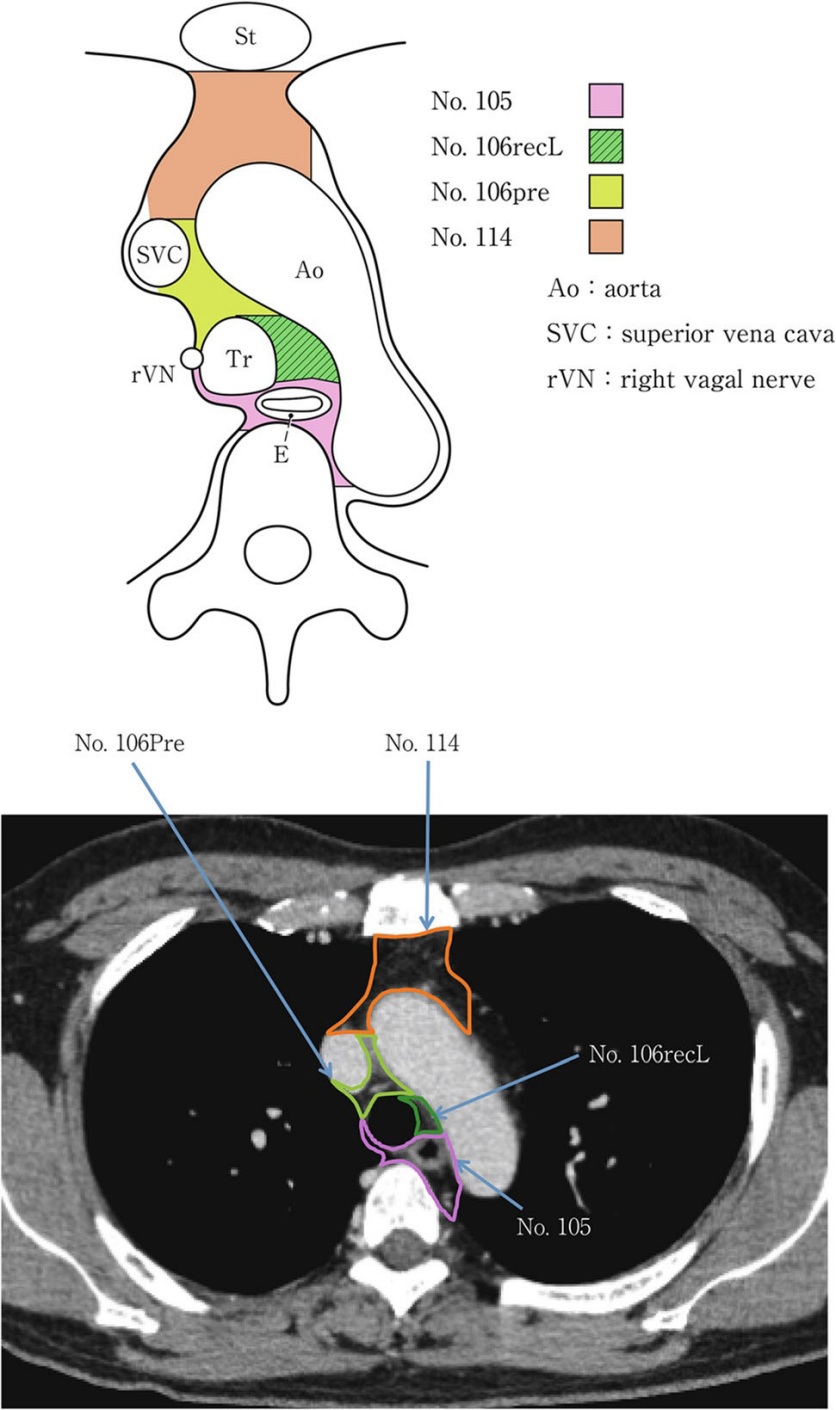
Upper mediastinal lymph nodes at the aortic arch level

**Fig. 42:**
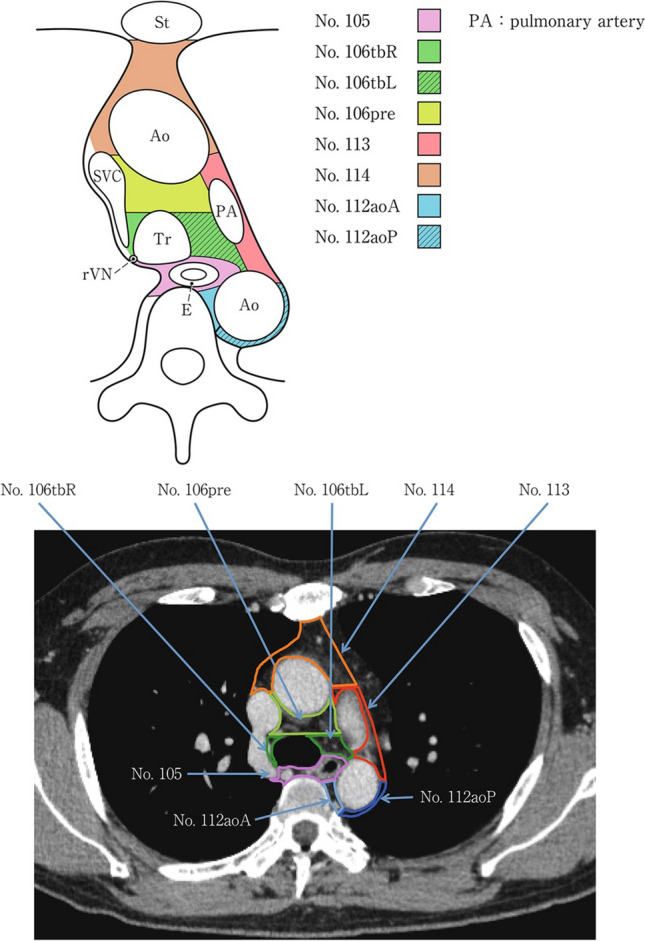
Upper mediastinal lymph nodes below the aortic arch level

**Fig. 43:**
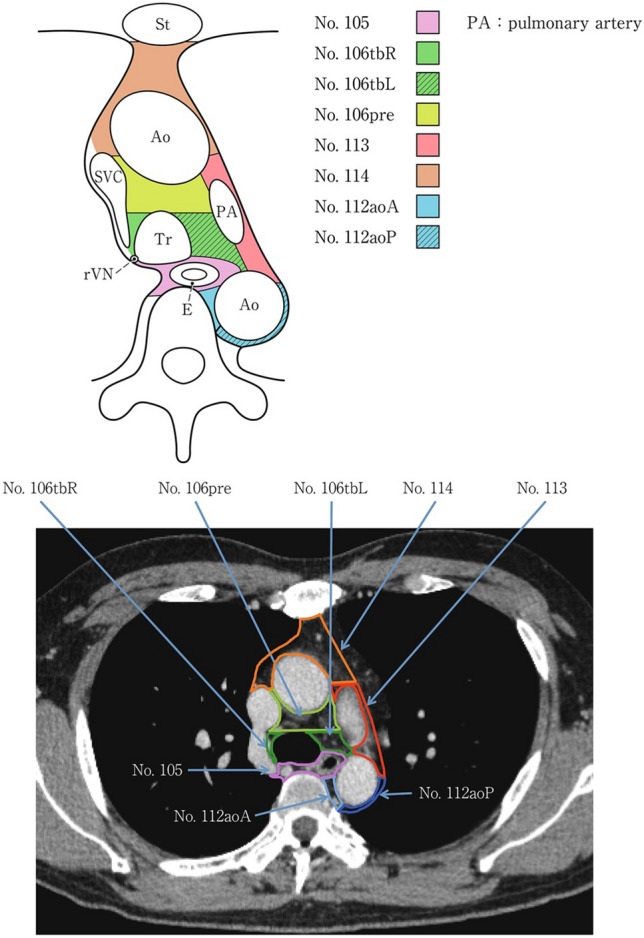
Mediastinal lymph nodes in the level below the carina

**Fig. 44:**
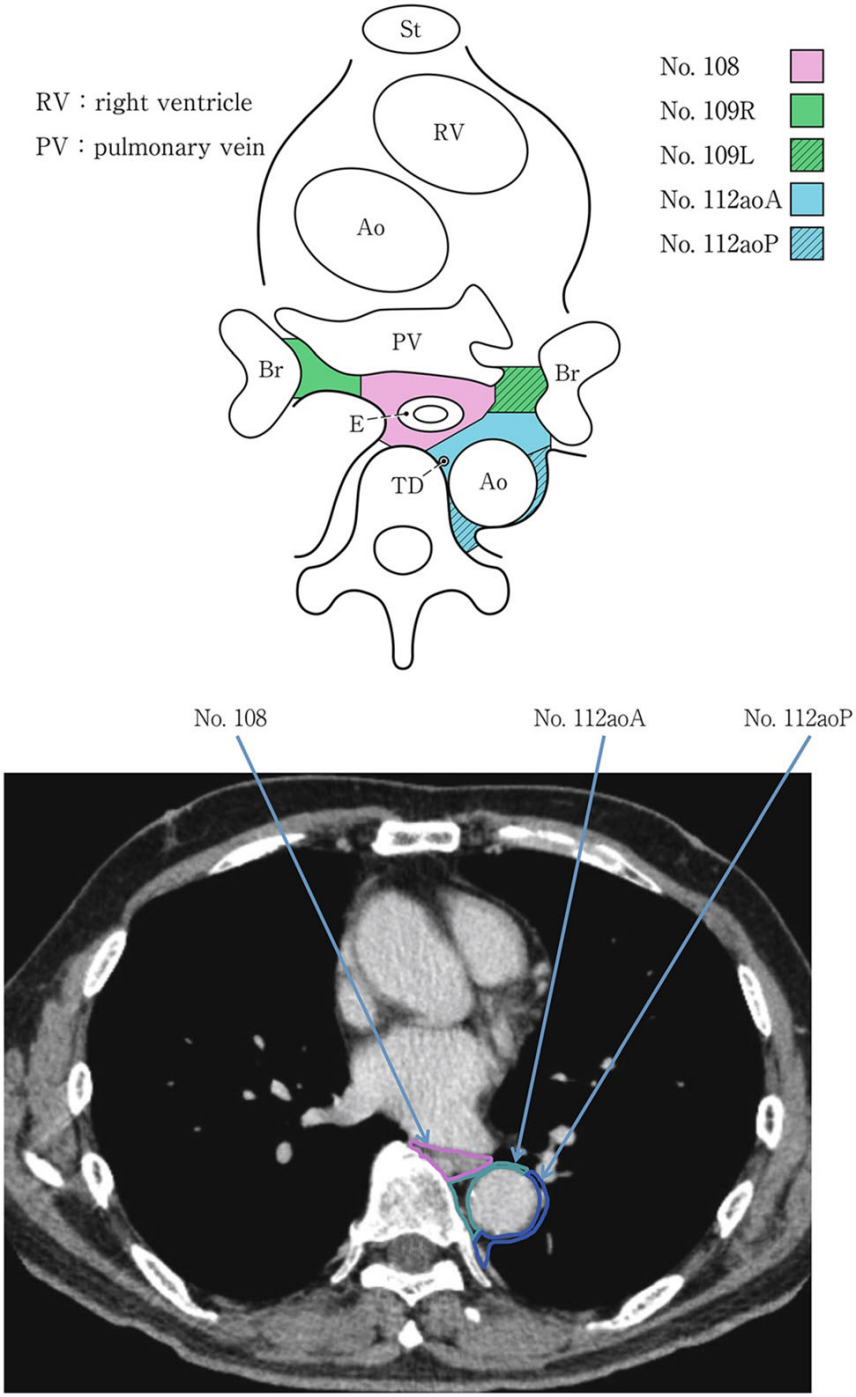
Lower mediastinal lymph nodes are in the inferior pulmonary vein level

**Fig. 45:**
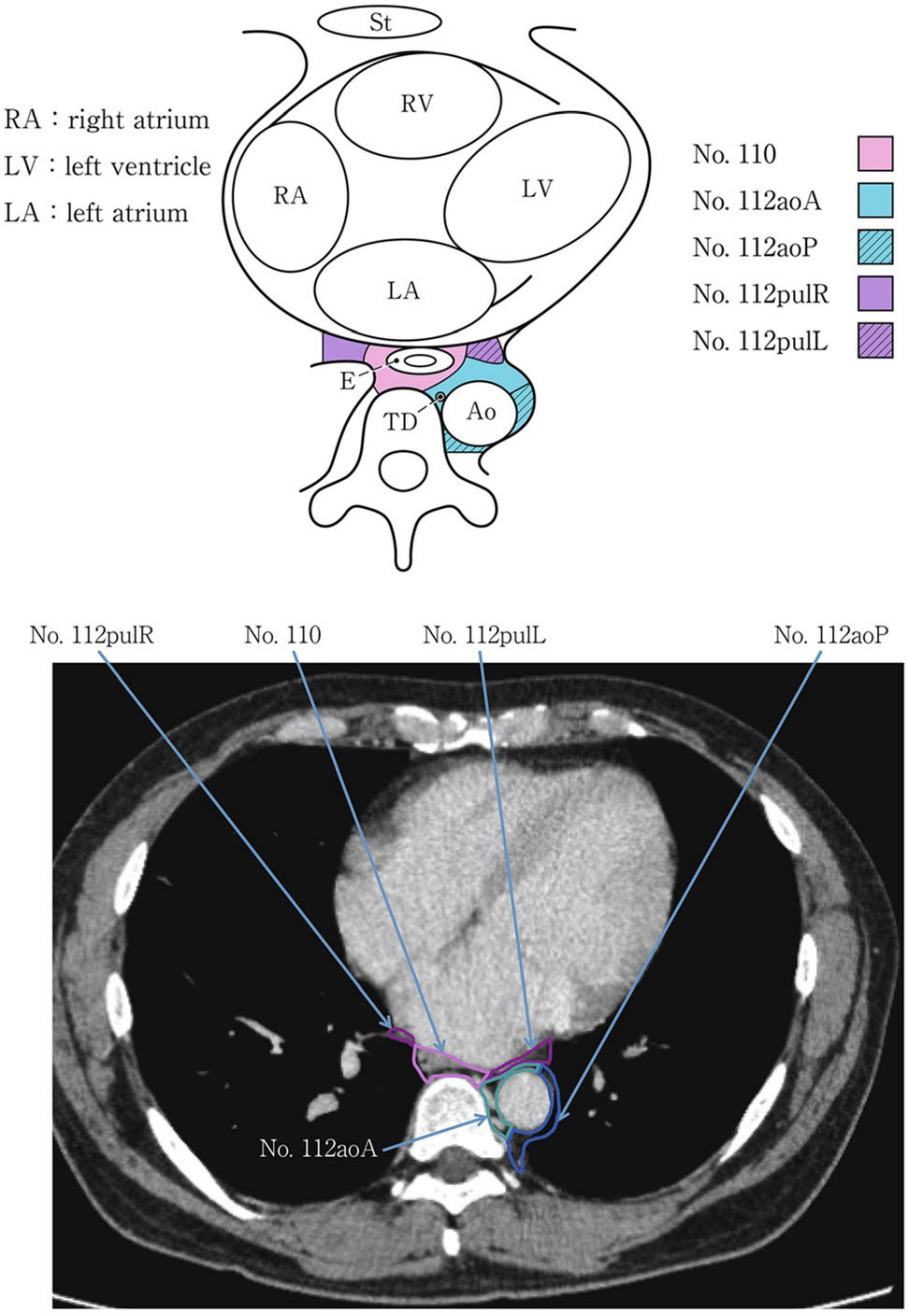
Lower mediastinal lymph nodes are in the right atrium level

**Fig. 46:**
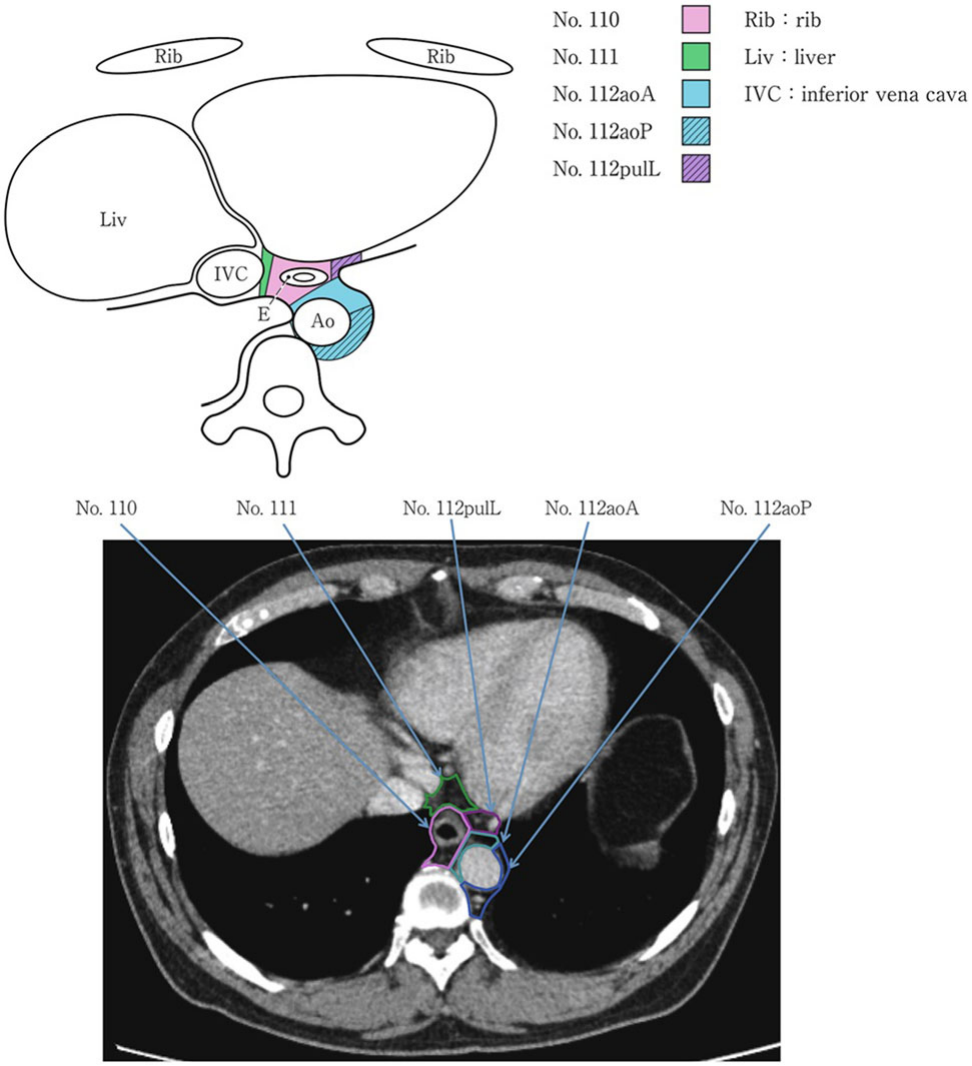
Lower mediastinal lymph nodes are in the level above the hiatus

### 19.3. Abdominal lymph nodes (Fig. 47)

The names, numbers, and extent of abdominal lymph nodes are defined by the “Japanese Classification of Gastric Carcinoma” [1]

**Fig. 47:**
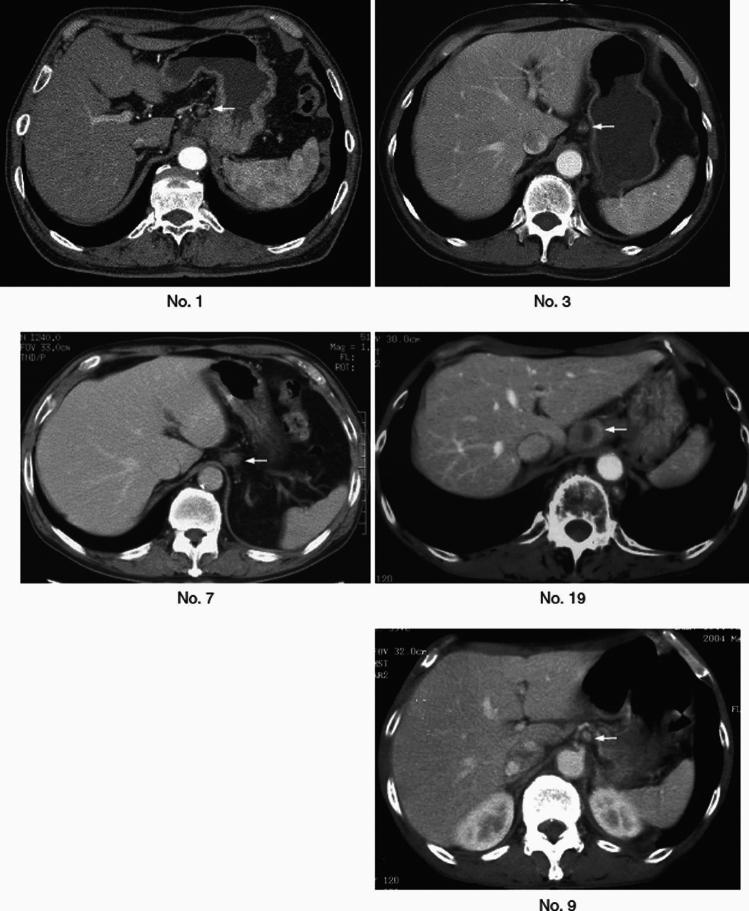
Abdominal lymph nodes

## 20. Diagnostic criteria of CT and PET–CT for lymph-node metastasis (refer to 3.1 in Part I)

The following data show the accuracy of the clinical diagnosis of lymph-node metastasis in patients with cT2-T4 tumors without neoadjuvant treatment (N = 224). Lymph nodes with a short axis ≥ 5 mm were examined.

The accuracies [sensitivity, specificity, positive predictive value (PPV), and negative predictive value (NPV)] are recorded for each cut-off value of 5, 6, and 7 mm on the short axis using CT.

PET–CT makes it difficult to set the cut-off value of SUVmax, because the values are affected by various factors (including machine, patient’s condition, protocol, and so on). Then, the accuracy (sensitivity, specificity, PPV, and NPV), which was determined based on each institution’s criteria, is recorded (Table 2).

**Table 2:** Accuracy of clinical diagnosis of lymph-node metastasis using CT or PET–CT

cT2–4	CT						PET–CT			
		Short axis	Sensitivity	Specificity	PPV	NPV	Sensitivity	Specificity	PPV	NPV
101, 106	Short axis ≥ 5 mm	5 mm	100	0	33.6	*	45	98.1	71.1	94.4
		6 mm	65.2	52.1	40.8	74.7				
		7 mm	37.5	72.5	40.9	69.6				
		8 mm	27.8	86.6	51.3	70.3				
1, 2, 3, 7	Short axis ≥ 5 mm	5 mm	100	0	60.3	*	28.8	98.3	65.2	92.4
		6 mm	81.8	39.7	67.3	59				
		7 mm	62.5	63.8	72.4	52.9				
		8 mm	52.3	74.1	75.4	50.6				

Abbreviations: *PPV* positive predictive value, *NPV* negative predictive value

## 21. Usefulness and validity of response criteria according to measurement method (refer to 14)

### 21.1. Correlation between the type of primary tumor measurement method and prognosis

**Measurements of tumor short diameter (refer to 14.1.1)**

For the primary lesion with ≥ 20 mm of long diameter in CT (axial), its short diameter is measured on the plane with longest diameter of the primary lesion.

Primary lesion reduction rate (∆tumor short diameter) = [(short diameter before treatment—short diameter after treatment) / (short diameter before treatment)] × 100%

**Fig. 48:**
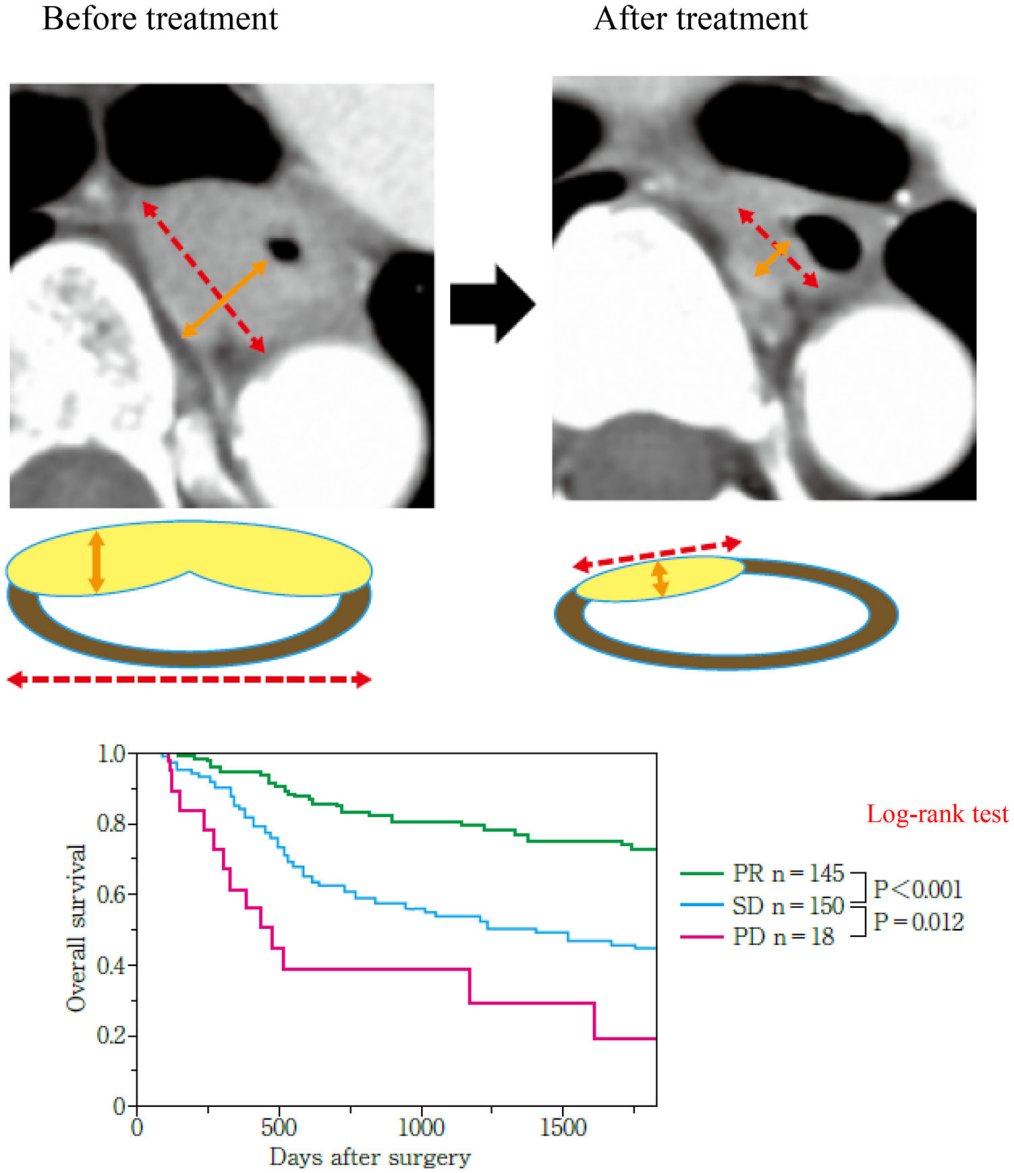
Correlation between the reduction rate of short diameter and survival

We present the study results on the correlation between treatment response and prognosis in patients with locally advanced esophageal cancer (*n* = 313) who underwent radical esophagectomy after preoperative chemotherapy [15] (Fig. 48).

**Measurements by a cross-section of the esophagus (refer to 14.1.2)**

When evaluating the primary lesion using the method of the short diameter becomes difficult, then the method using the reduction rate of the cross-section area of the esophagus can be used as an alternative for the primary lesion with ≥ 20 mm of long diameter in CT (axial) before treatment. This method is not applicable to esophagogastric junction cancer (Jz).

The cross-sectional area is calculated by multiplying the long esophageal diameter by the short esophageal diameter in the plane with the longest diameter of the primary lesion.

Reduction rate of cross-section area (∆ esophageal cross-section area) = [(the cross-section area before treatment − the cross-section area after treatment) / (the cross-section area before treatment)] × 100%

**Fig. 49:**
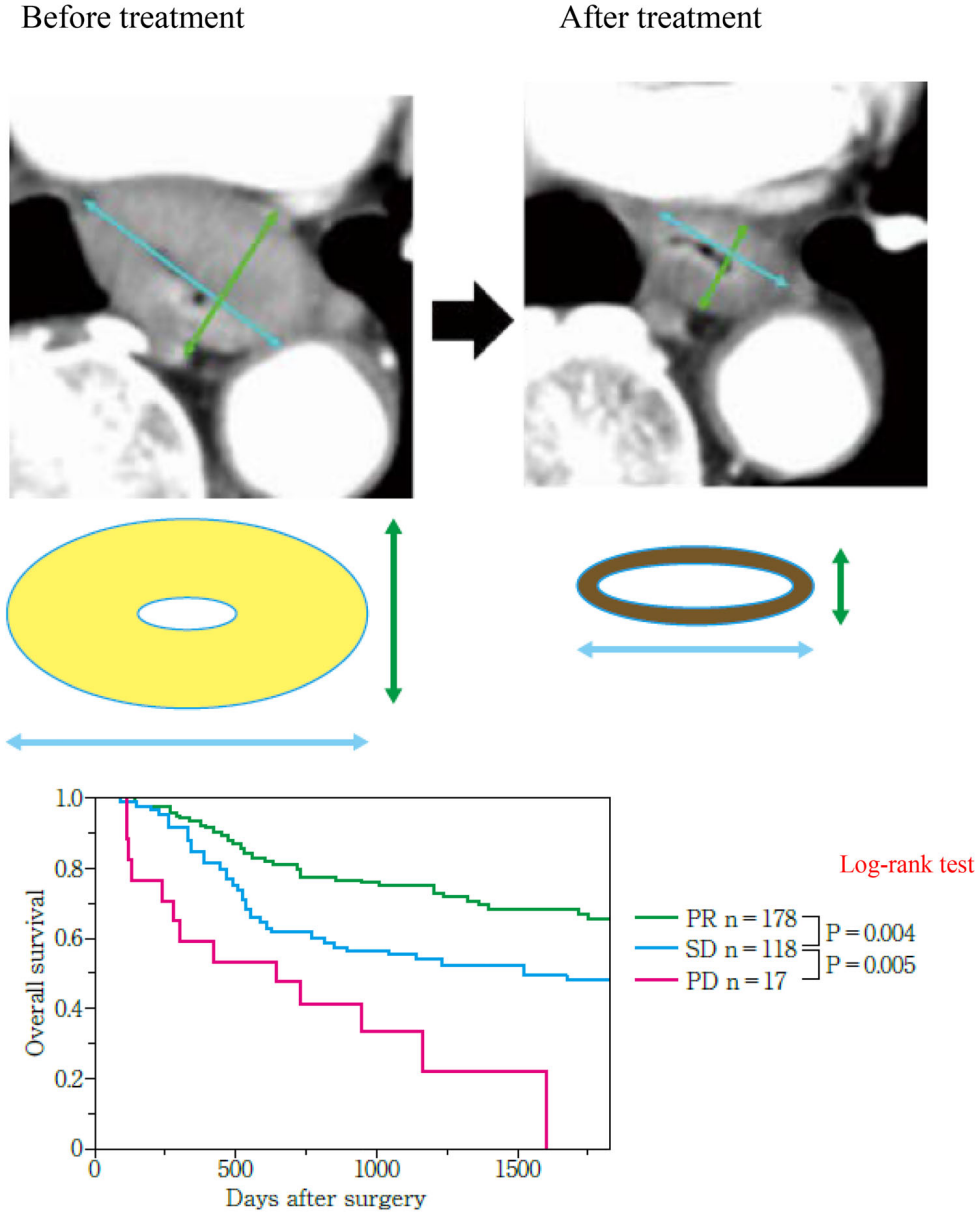
Correlation between reduction rate of the cross-section area and survival

We present our study results on the correlation between treatment response and prognosis in patients with locally advanced esophageal cancer (*n* = 313) who underwent radical esophagectomy after preoperative chemotherapy [14] (Fig. 49).

### 21.2. Correlation between the reduction rate in SUVmax and prognosis in PET–CT (refer to 14.2)

The following are the results of a study on the correlation between the reduction rate of SUVmax on PET/CT before and after treatment and the prognosis in patients with locally advanced esophageal cancer (n = 220) who underwent radical esophagectomy after preoperative chemotherapy [16].

**Fig. 50:**
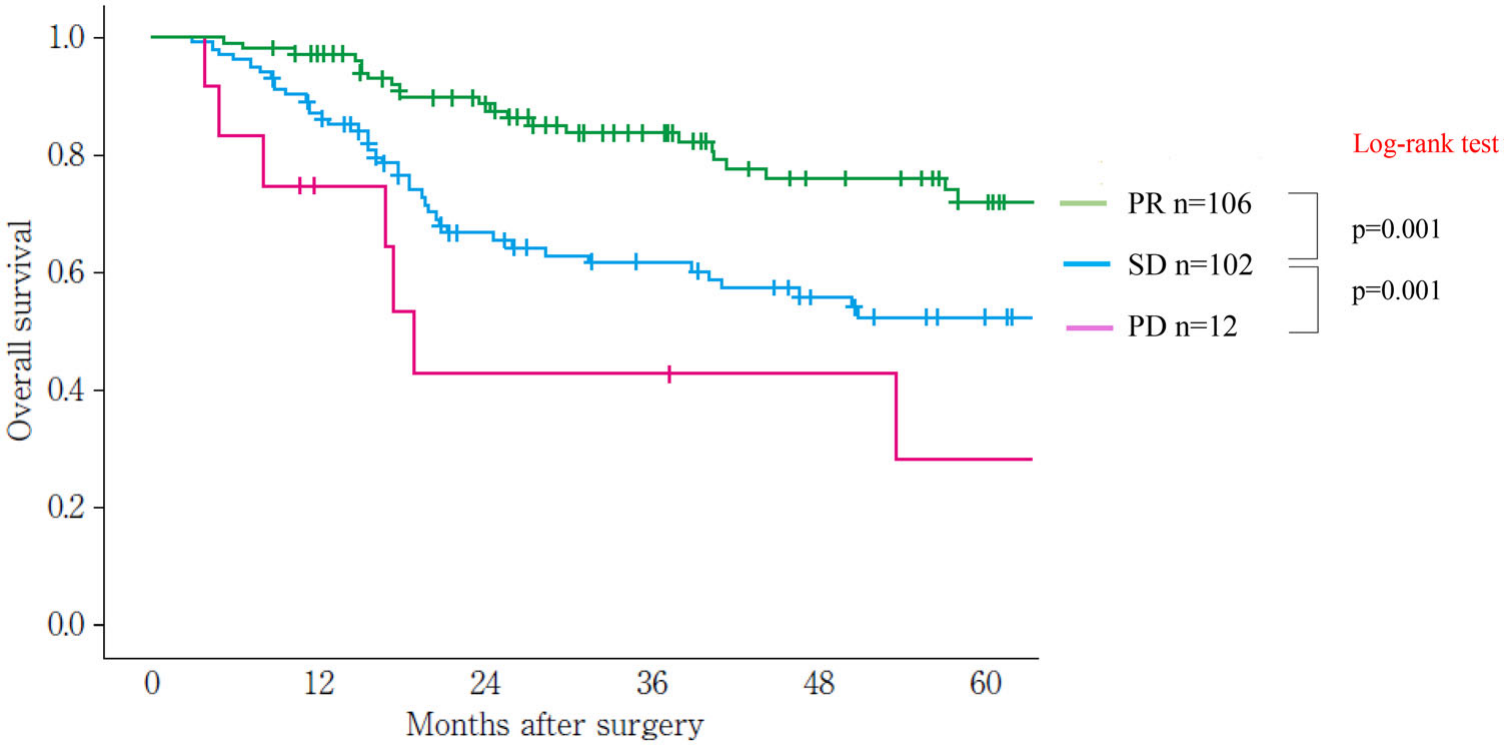
Correlation between reduction rate in SUVmax in PET–CT and survival

In the case of the primary tumor with cT2-4 and SUVmax ≥ 3 before treatment, the reduction rate of SUVmax is calculated (Fig. 50).

Reduction rate of SUVmax = [(SUVmax before treatment − SUVmax after treatment) / (SUVmax before treatment)] × 100%.

## 22. Response evaluation criteria for primary lesion using endoscopy (refer to 15.)

### 22.1 Endoscopic findings of CR cases (refer to 15.1.) (Figs. 51, 52)

**Fig. 51:**
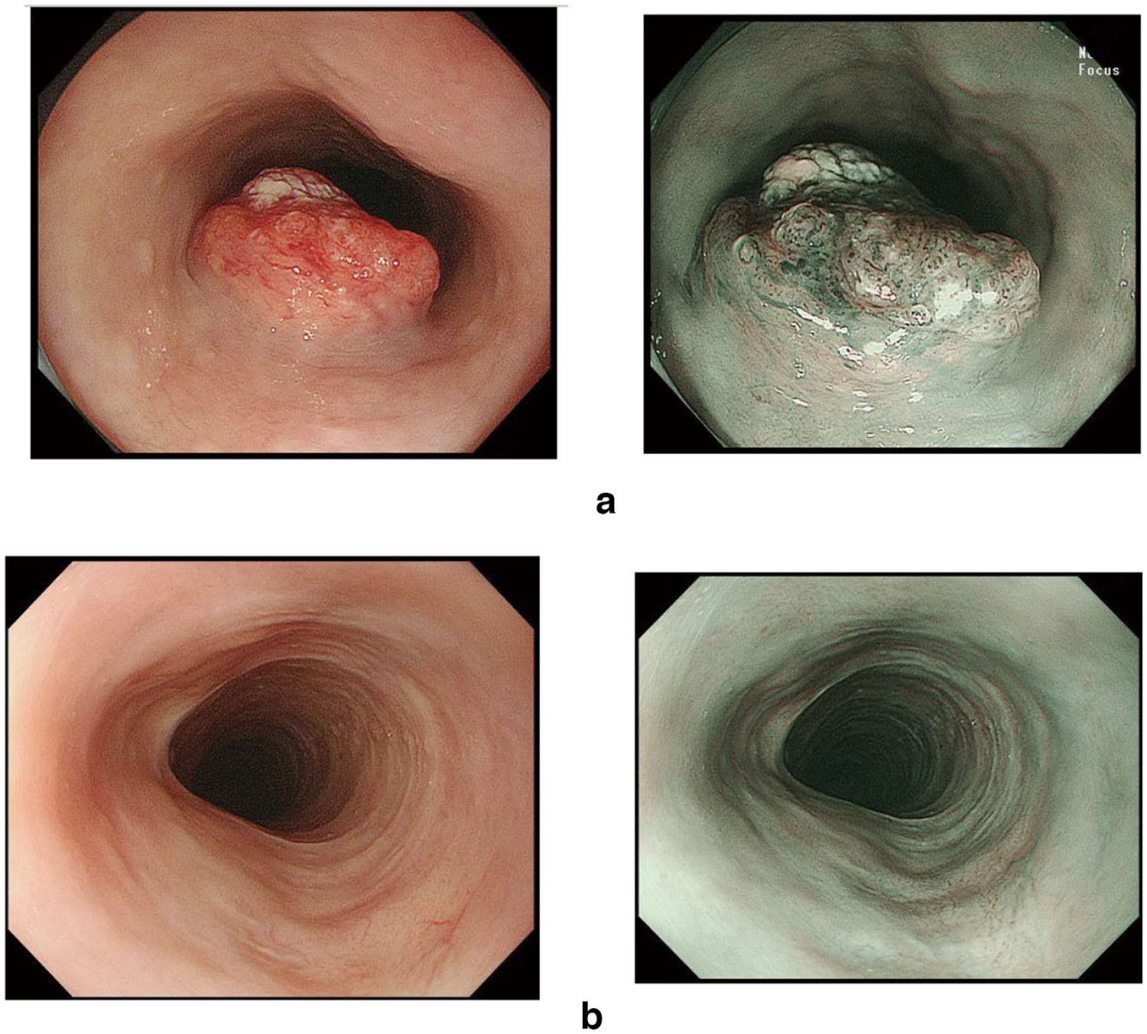
**a** Before treatment: Type 1 cT3. **b** After treatment (3 months after chemoradiation therapy (CRT)). The tumor has disappeared and only one scar remains. This case is judged to have achieved CR

**Fig. 52:**
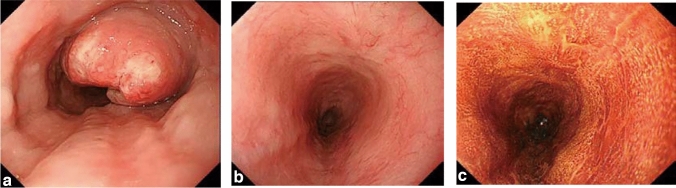
**a** Before treatment: Type 2, cStage III. **b** After treatment (6 months after CRT). The tumor has disappeared and only a scar remains. **c** After treatment (6 months after CRT). The esophageal mucosa has been stained brown. No unstained areas are present. An endoscopic biopsy from the area of the primary tumor is negative. This case is judged to have achieved CR

### 22.2 Endoscopic findings of non-CR/non-PD cases (refer to 15.2.) (Figs. 53, 54)

**Fig. 53:**
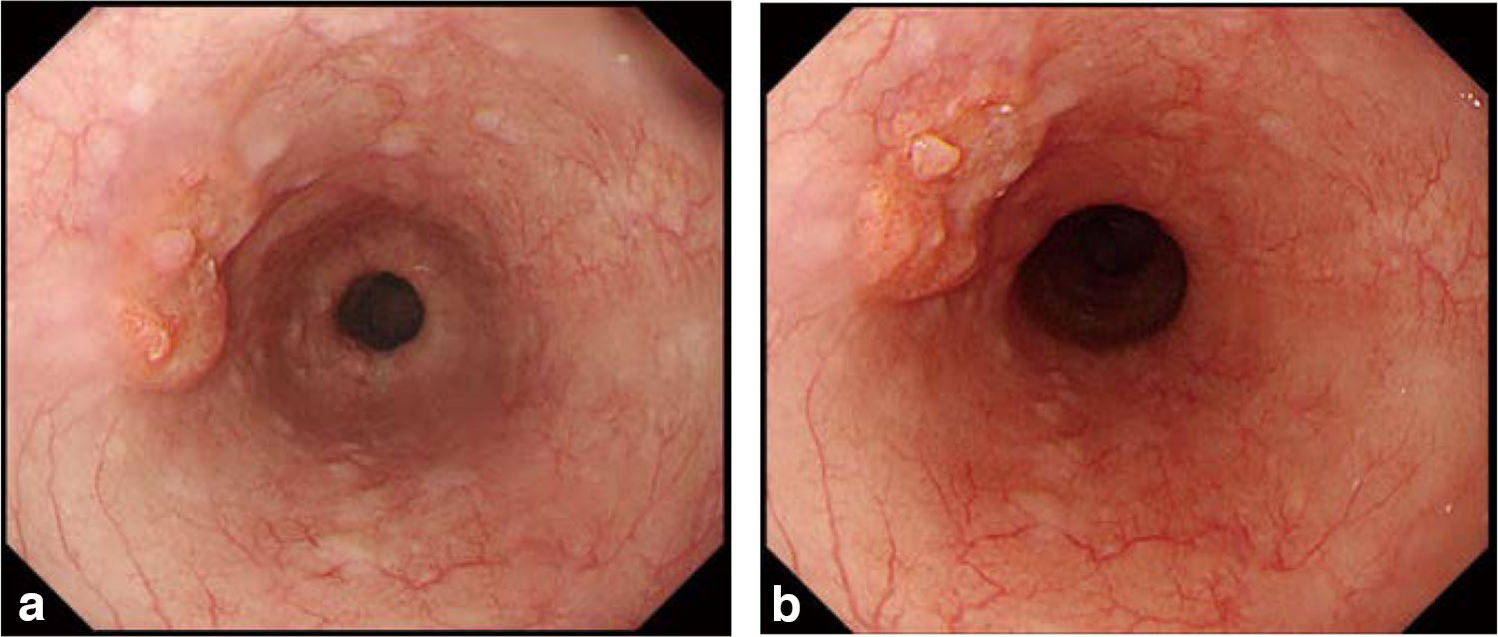
**a** Before treatment: Type 0–III cT1b. **b** After treatment (2 months after neoadjuvant chemotherapy: 5FU, cisplatin (FP)  2 courses): Tumor appears unchanged and is judged as non-CR/non-PD

**Fig. 54:**
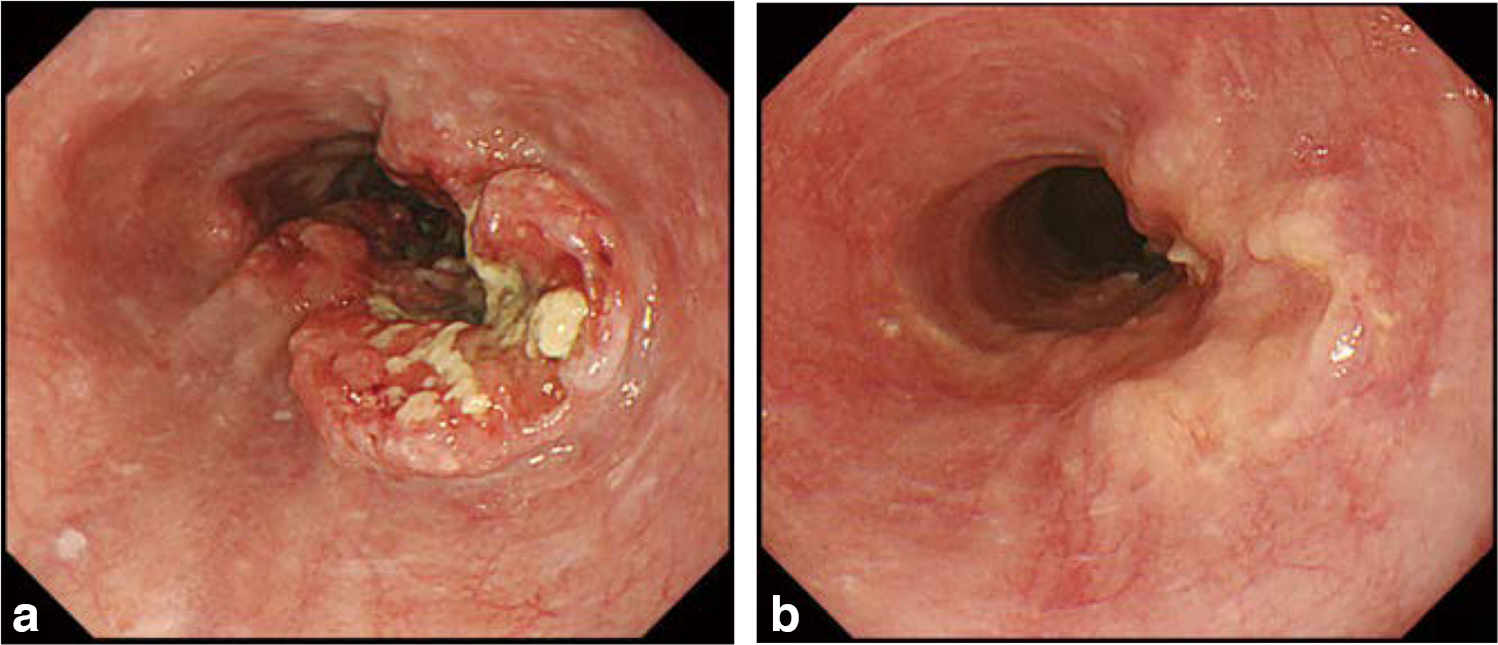
**a** Before treatment: Type 2 cT3. **b** After treatment (2 months after neoadjuvant chemotherapy: FP 2 courses): Although the tumor shrinks, it is judged as non-CR/non-PD, because it is judged that there are residuals equivalent to T2

### 22.3 Endoscopic findings of non-CR/non-PD (RR) cases (refer to 15.2.1.) (Figs. 55, 56, 57)

**Fig. 55:**
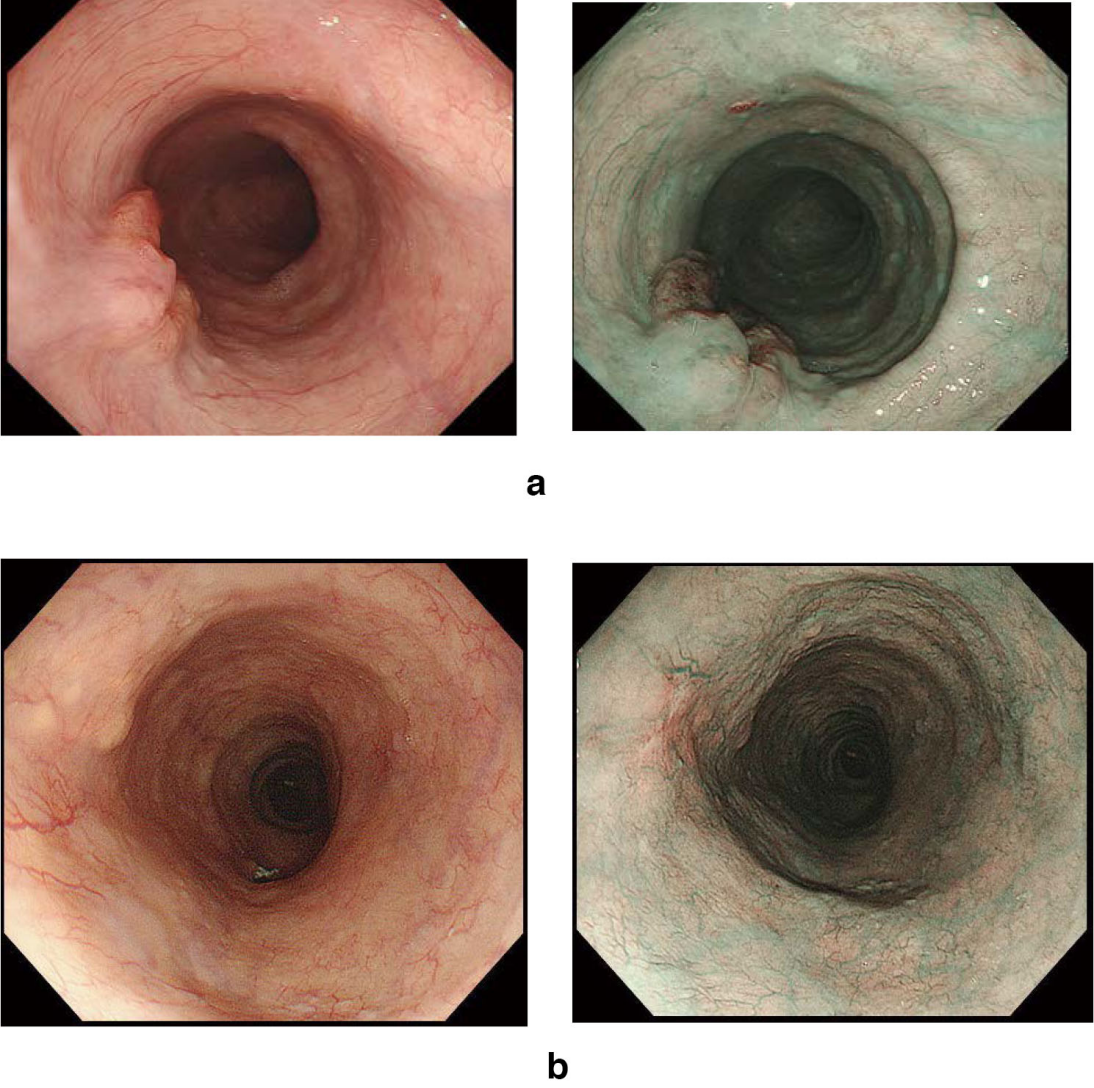
**a** Before treatment: Type 2 cT2. **b** After treatment (1 month after CRT): The tumor is markedly reduced and flattened; however, there is a submucosal-like elevation (equivalent to T1), which is determined to be RR

**Fig. 56:**
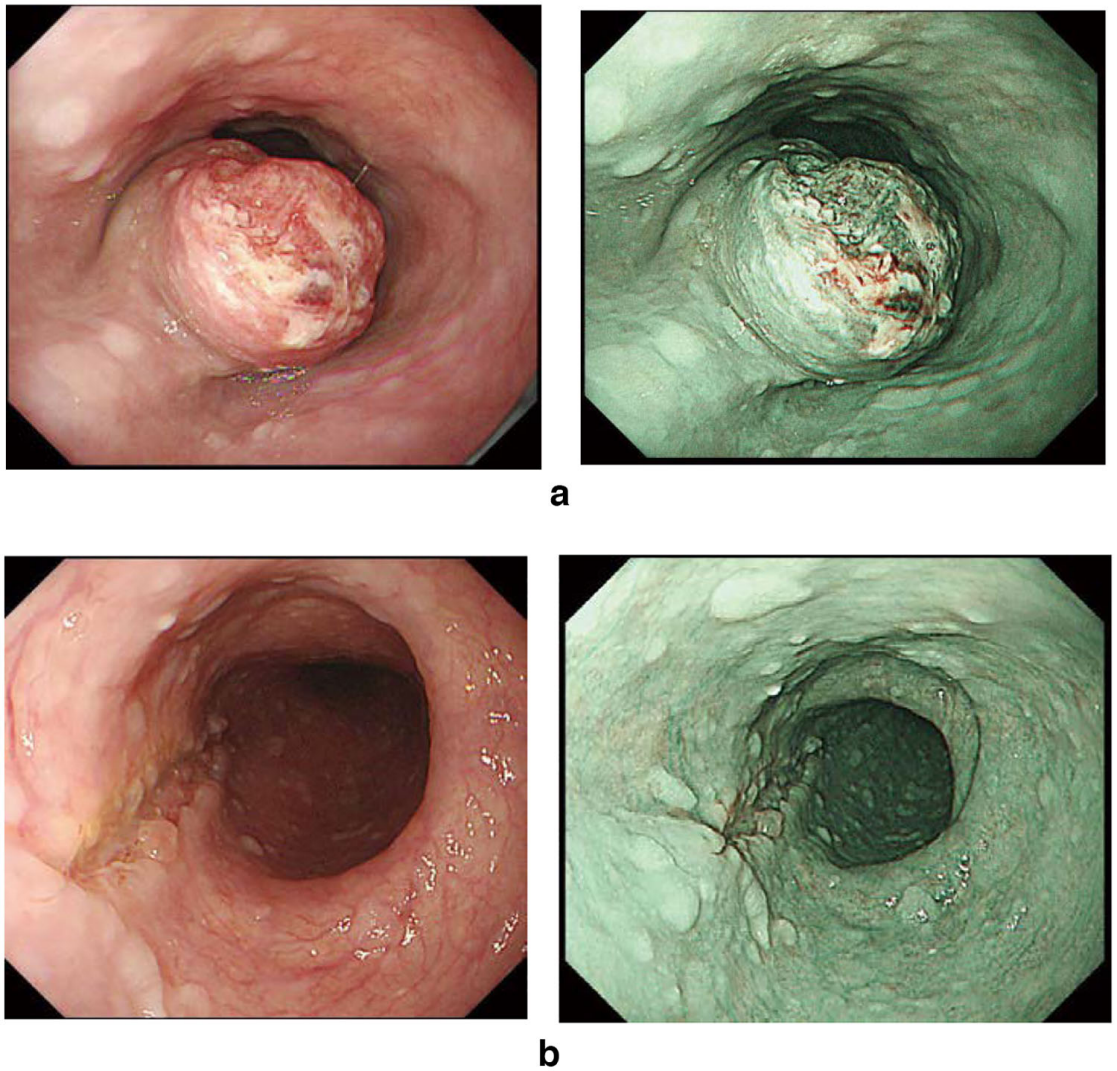
**a** Before treatment: Type 1 cT3. **b** After treatment (2 months after neoadjuvant chemotherapy: docetaxel, cisplatin, 5FU (DCF) 3 courses): The tumor shrinks markedly; however, there is a remnant equivalent to T1, which is judged to be RR

**Fig. 57:**
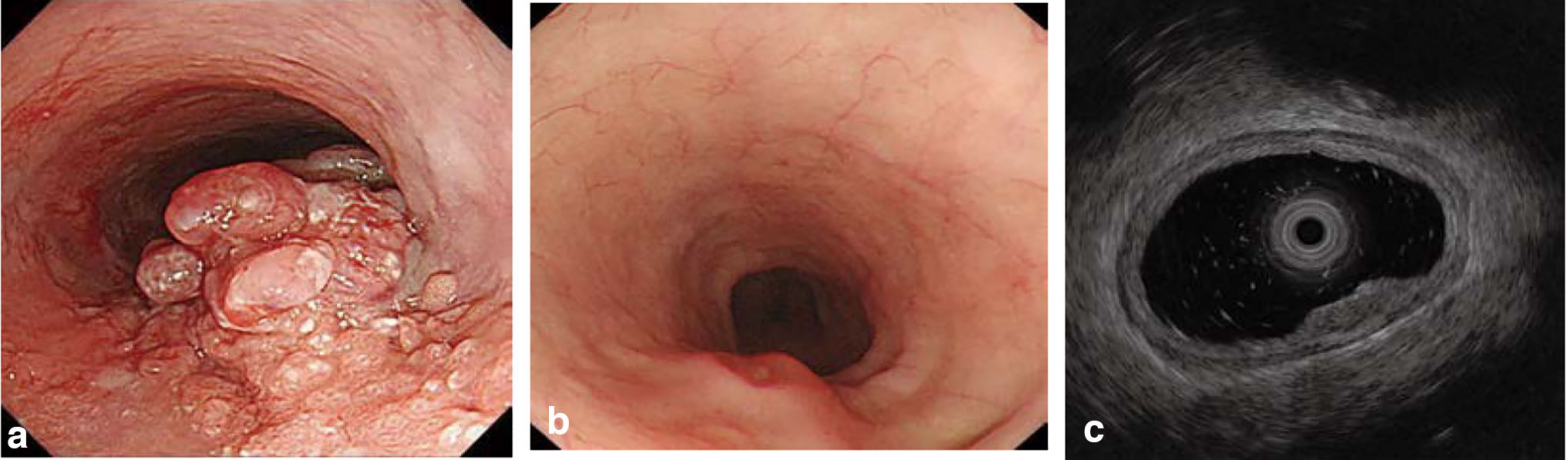
**a** Before treatment: Type 2 cT3. **b** After treatment (1 month after CRT): Tumor is markedly reduced and flattened, but there is a submucosal-like elevation (equivalent to T1), and it is judged as RR. c Endoscopic ultrasound. Ultrasound endoscopy also shows a low-absorption echo to the submucosal layer, and it is judged as RR

The tumor is markedly reduced and flattened; however, there is a submucosal-like elevation (equivalent to T1), which is determined to be RR.

### 22.4 Endoscopic findings for a PD case (refer to 15.3.)

See (Fig. 58)

**Fig. 58:**
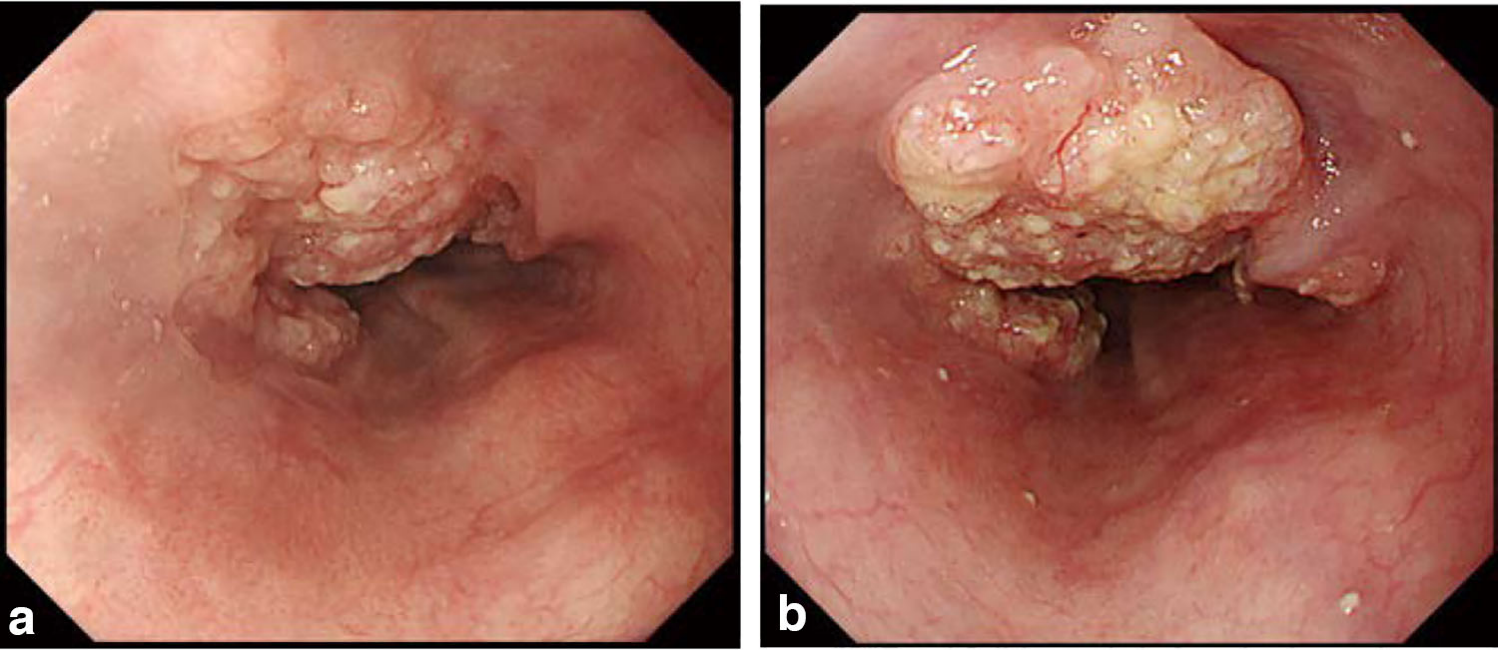
**a** Immediately after preoperative chemotherapy (DCF2 courses). **b** Immediately before surgery (2 weeks after preoperative chemotherapy). Tumor is increasing in size and is judged as PD

### 22.5 Endoscopic findings of LR cases (refer to 15.4.)

**Fig. 59:**
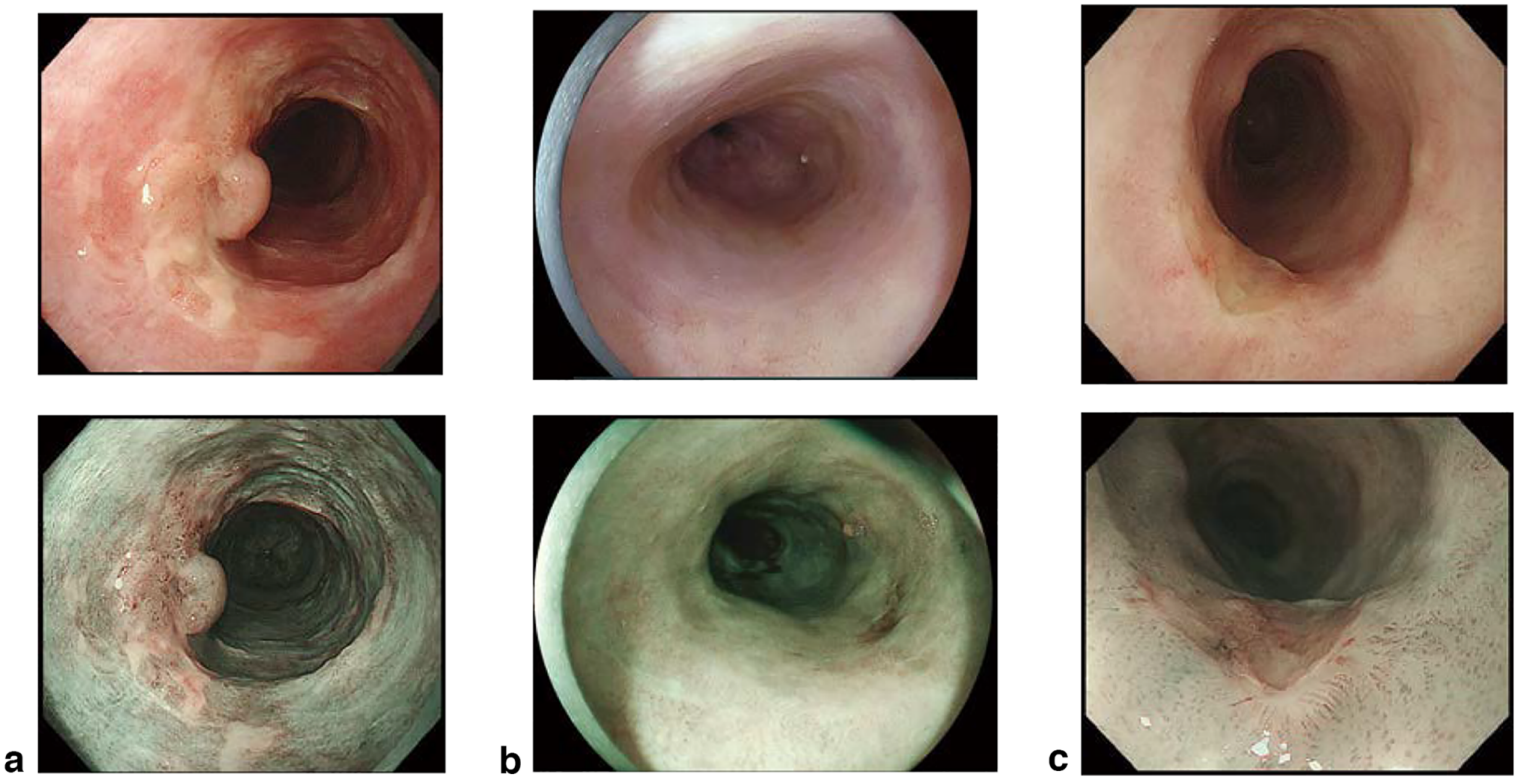
**a** Before treatment: Type 0-IIa + “0-I” cT1b. **b** After treatment (4 months after CRT): The tumor has disappeared and only a scar remains. This case is believed to be as a CR at this time. **c** Four months after CR judgment. Erosion appears at the primary tumor site. This case is judged as LR and salvage PDT is performed

**Fig. 60:**
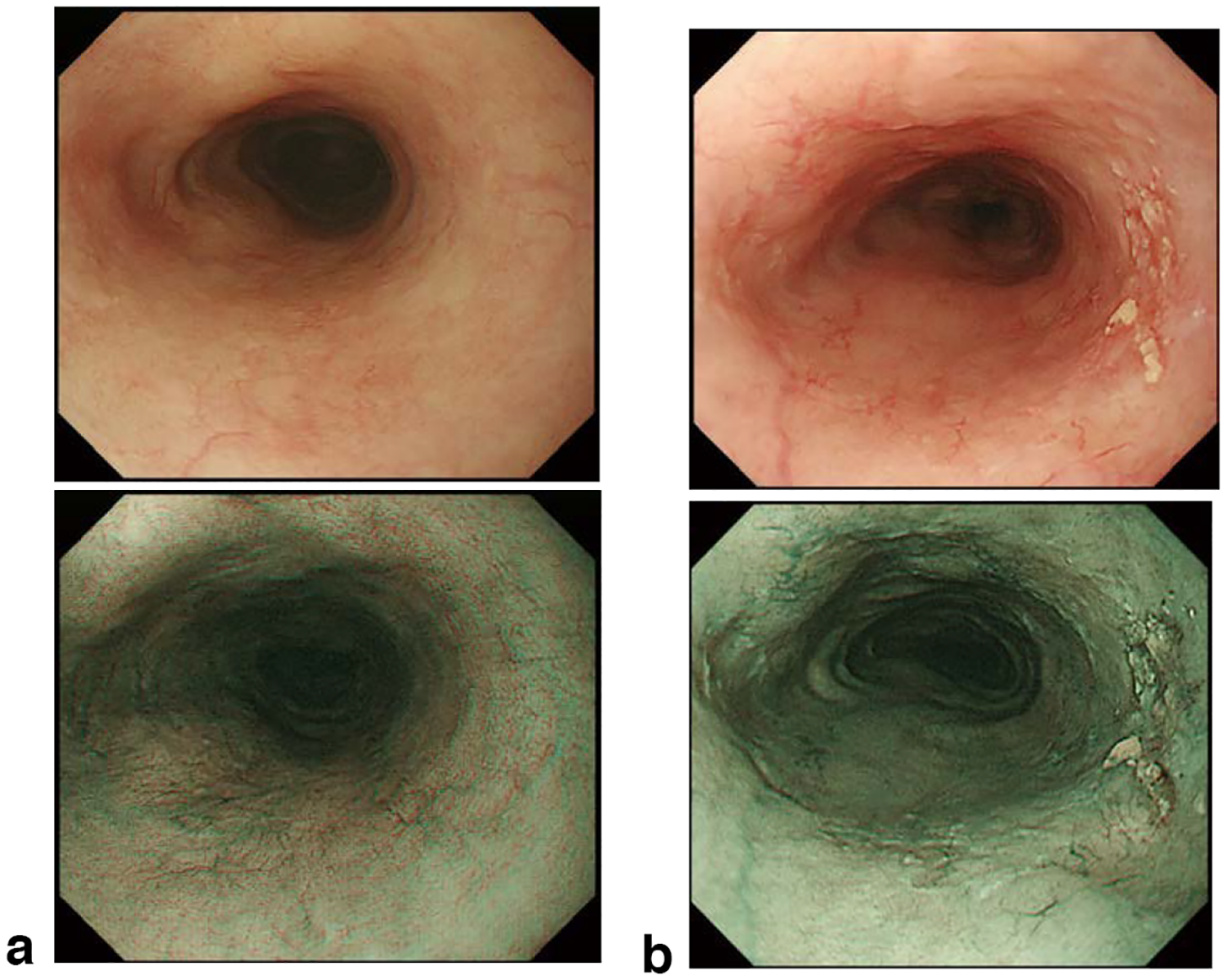
**a** After treatment (after CRT). cT1b lesion is judged to be CR. **b** Three years after CR judgment. Erosion appears at the primary tumor site. This case is judged as LR, and salvage endoscopic submucosal dissection (ESD) is performed

**Erosion** (Figs. 59, 60).

**Fig. 61:**
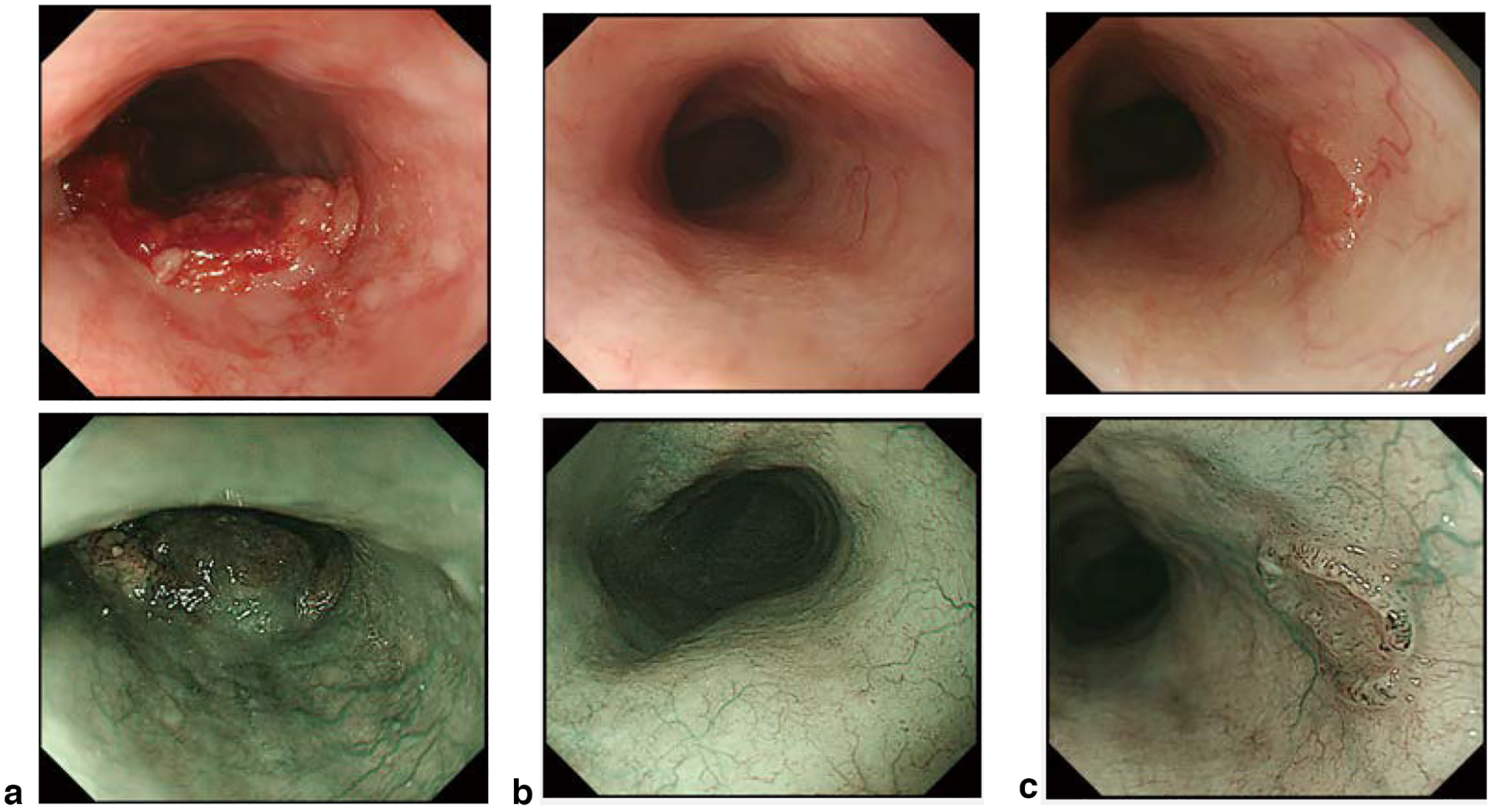
**a** Before treatment: Type 2 cT3. **b** After treatment (3 months after CRT). The tumor has disappeared and only a scar remains. This case was judged as a CR at this time. **c** Three months after CR judgment. The primary lesion is determined to be LR due to the appearance of an irregular and uneven area, and salvage EMR is performed

**Fig. 62:**
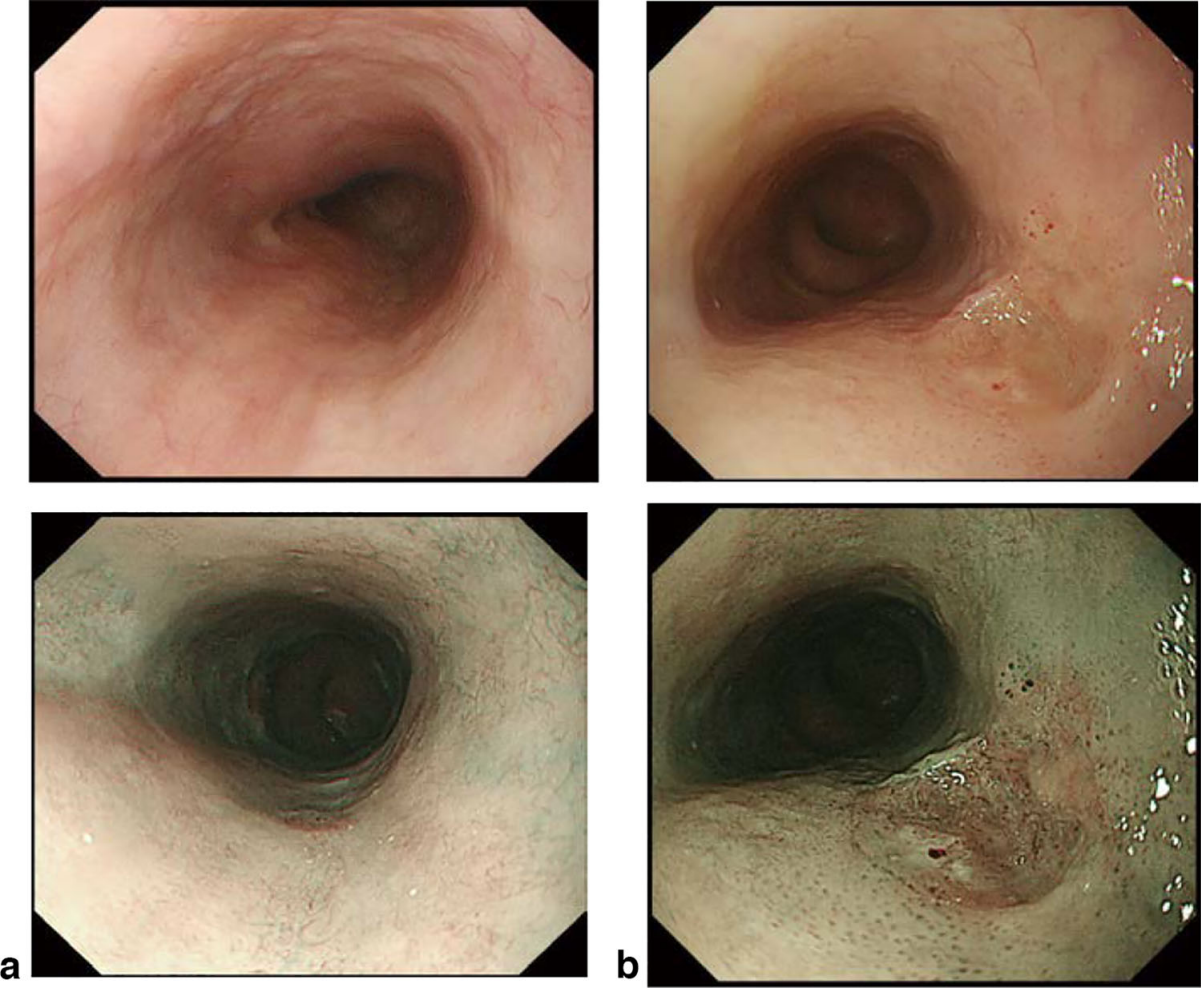
**a** After treatment (after CRT). The tumor has disappeared and only a scar remains. This case is judged as a CR at this time. **b** Two months after CR judgment. The primary lesion is determined to be LR due to the appearance of an irregular and uneven area, and salvage photodynamic therapy (PDT) is performed

**Fig. 63:**
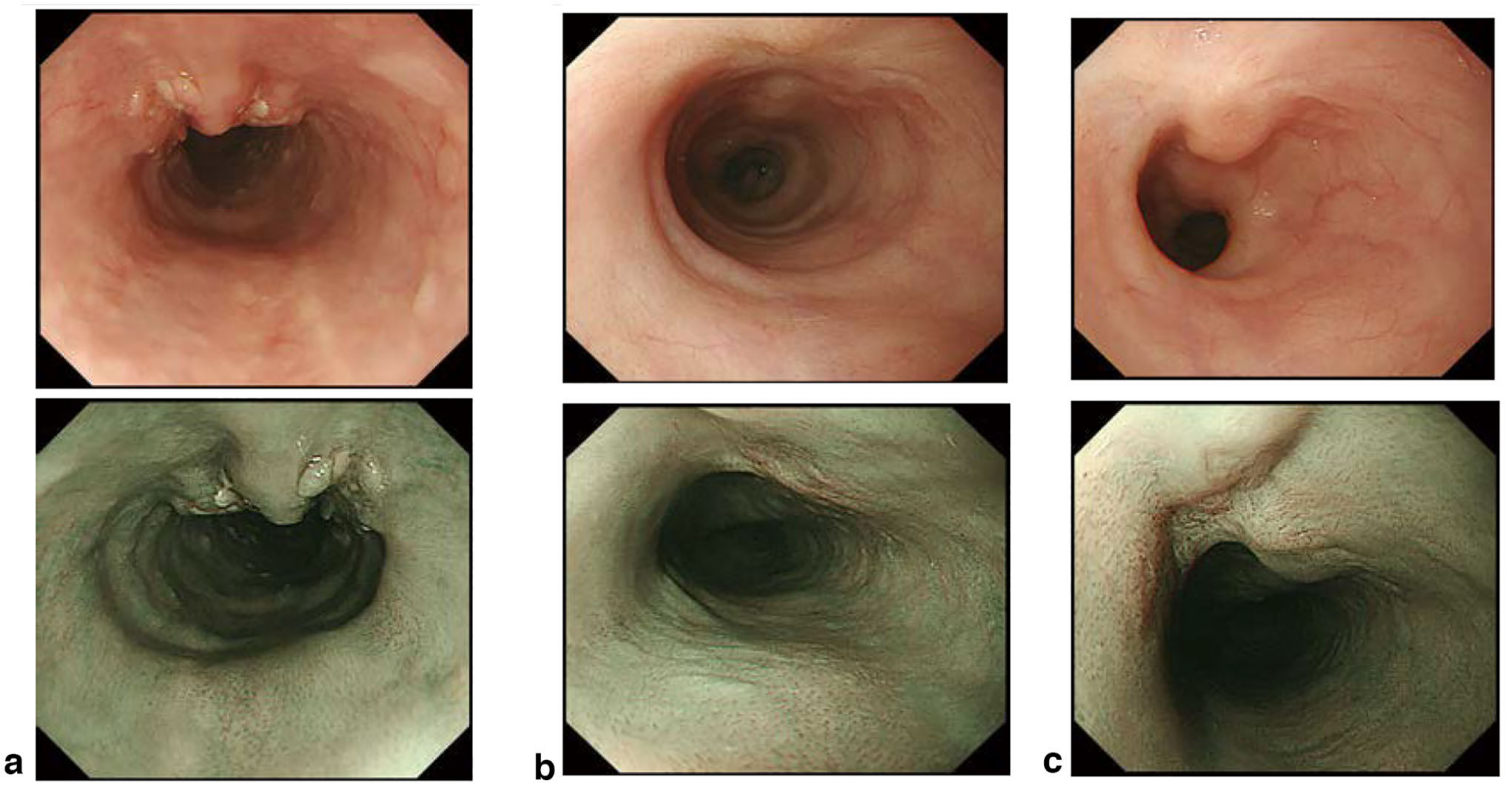
**a** Before treatment: Type 2 cT2. **b** After treatment (3 months after CRT): The tumor has disappeared and only a scar remains. This case was judged as a CR at this time. **c** Six months after CR judgment. The primary lesion is determined to be LR due to the appearance of a submucosal tumor (SMT)-like elevation, and palliative chemotherapy is performed

**Fig. 64:**
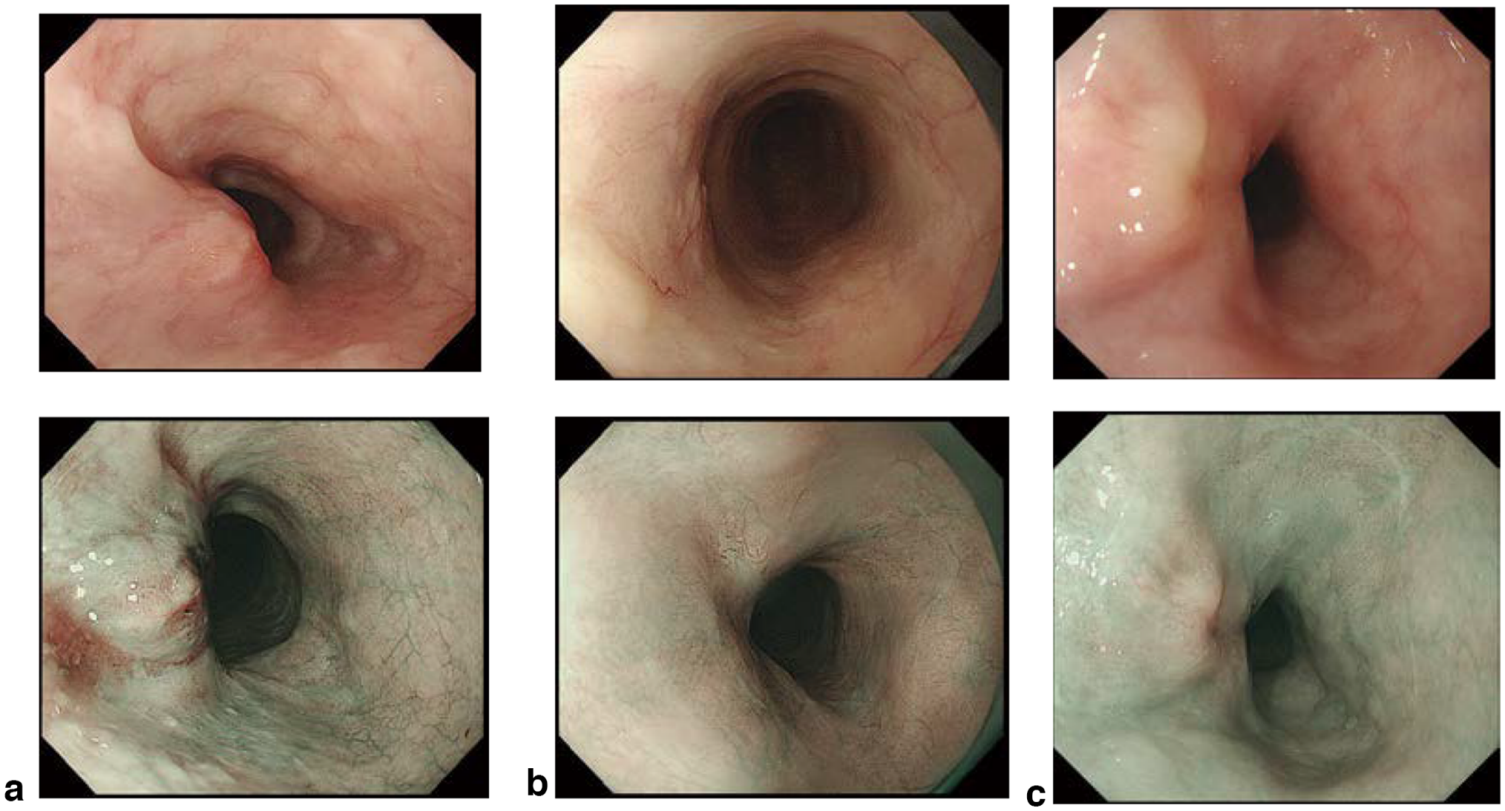
**a** Before treatment: Type 1 cT3. **b** After treatment (5 months after CRT): The tumor has disappeared and only a scar remains. This case was judged as a CR at this time. **c** Three months After CR judgment. The primary lesion is determined to be LR due to the appearance of a SMT-like elevation, and salvage surgery is performed

**Unevenness and irregularity** (Fig. 61, 62,63, 64).

## 23. Histological criteria for the effects of chemotherapy and/or radiation

**Fig. 65:**
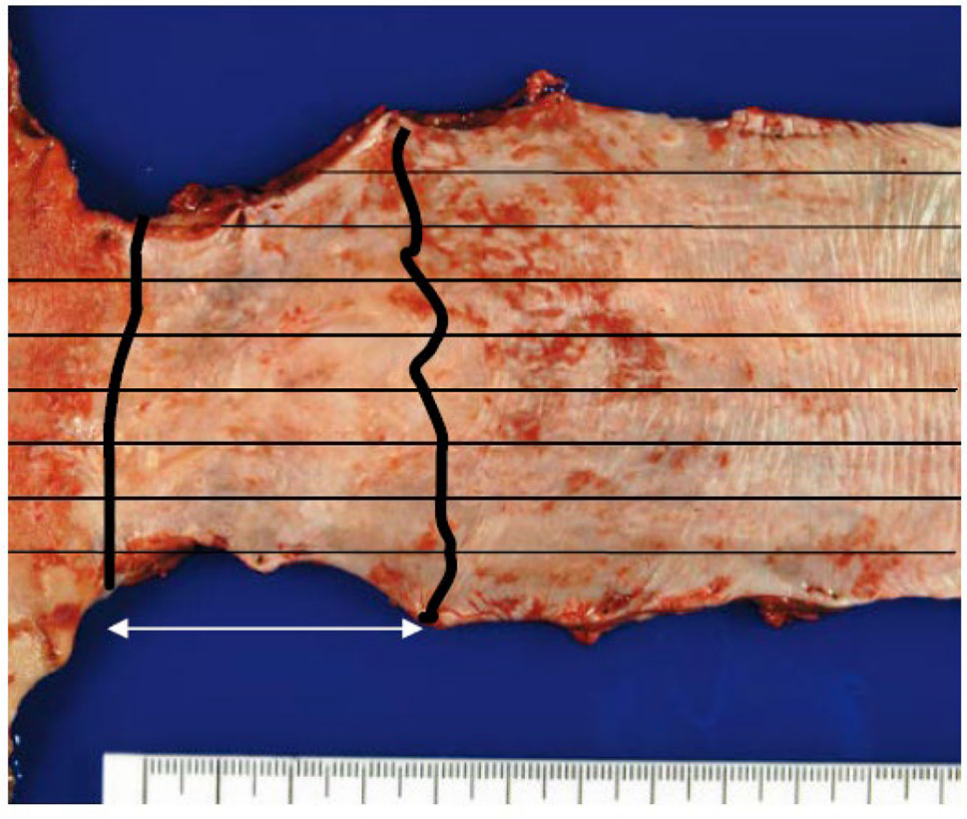
Resected specimen receiving CRT. Preoperative chemoradiotherapy was administered to Type 2 advanced cancer. In the resected specimen, only a mild stricture and wall thickening (arrow) are observed in the lower esophagus. Serial sections are prepared for the pathological examination. Histologically, fibrosis is observed in the stricture, which can be regarded as the extent of the preexisting tumor

**Fig. 66:**
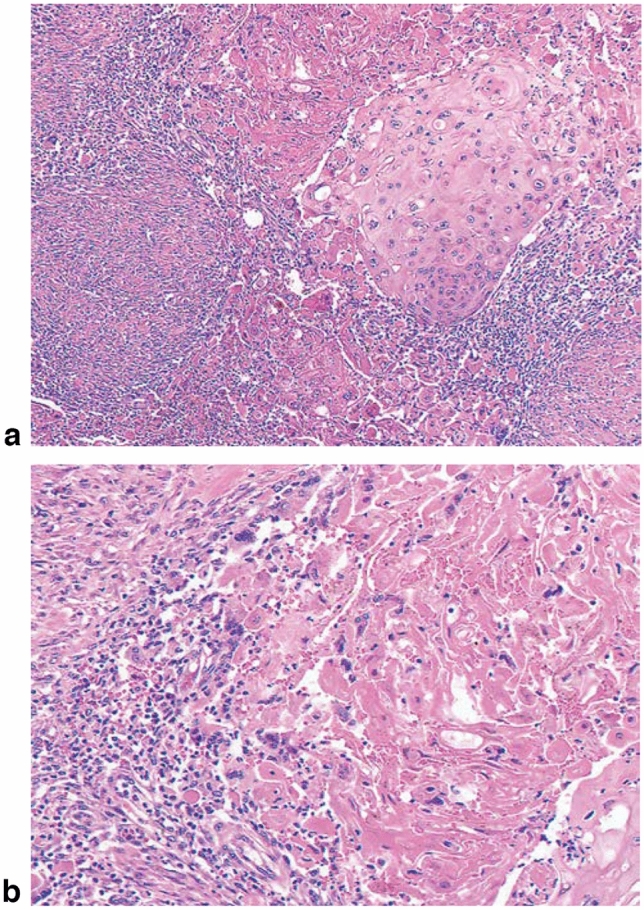
Grade 1: Slightly effective. **a** Middle-power magnification. **b** High-power magnification view of Figure 66 a

See Figs. 65 and 66.

**Fig. 67:**
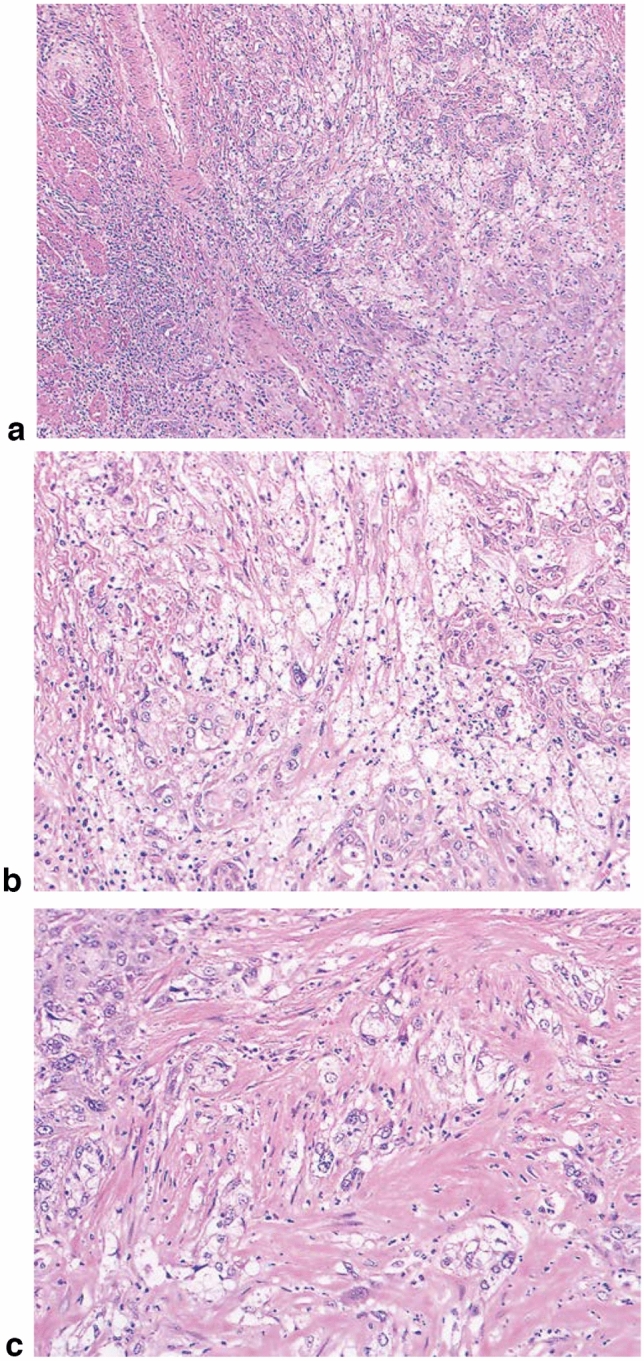
Grade 2: moderately effective. **a** Low-power magnification. **b** High-power magnification view of Figure 67a. **a** and **b** Small nests of a tumor are surrounded by macrophages with foamy cytoplasm. Most of the residual cancer cells show degeneration and decreased staining with eosin. Tumor nests are surrounded by inflammatory cells. The foamy cells are regarded as a reaction to liquefactive necrosis. **c** Another section of the same case Scattered tumor cells show vacuolation of the cytoplasm and nuclei, and are surrounded by marked fibrosis (x200)

**Fig. 68:**
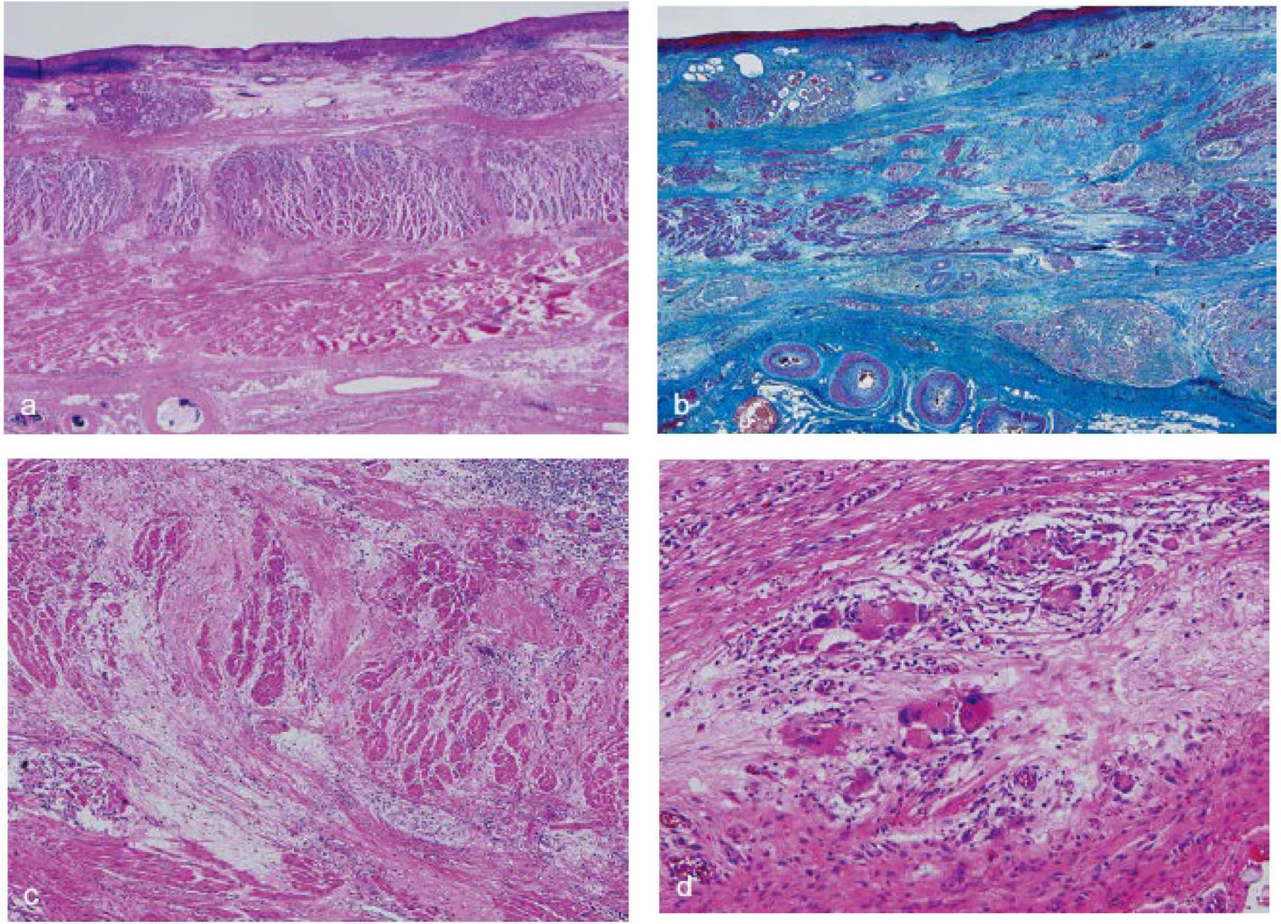
Grade 3: Markedly effective. **a** Marked fibrosis is noted beneath the squamous epithelium. **b** Masson staining reveals partial disruption of the muscular layer. Fibrosis was observed throughout the esophageal wall. The area of fibrosis can be regarded as the area of preexisting tumors. **c** Disruption of the muscularis propria and fibrosis is noted. **d** No viable cancer cell is observed, while foreign body giant cells are scattered. The therapeutic effect was grade 3

Tumor nests with abundant keratinization have unclear borders, surrounded by inflammatory cell infiltrates, giant foreign body cells, and granulation tissue with marked fibrosis. Viable tumor cells are observed at the center of the tumor. A characteristic finding is that the keratinized material directly contacts the granulation tissue without a basal layer or prickle cell layer. Numerous viable tumor nests are also observed in other sections (Figs. 67, 68).

## 24. Survival curve according to stage

**Fig. 69:**
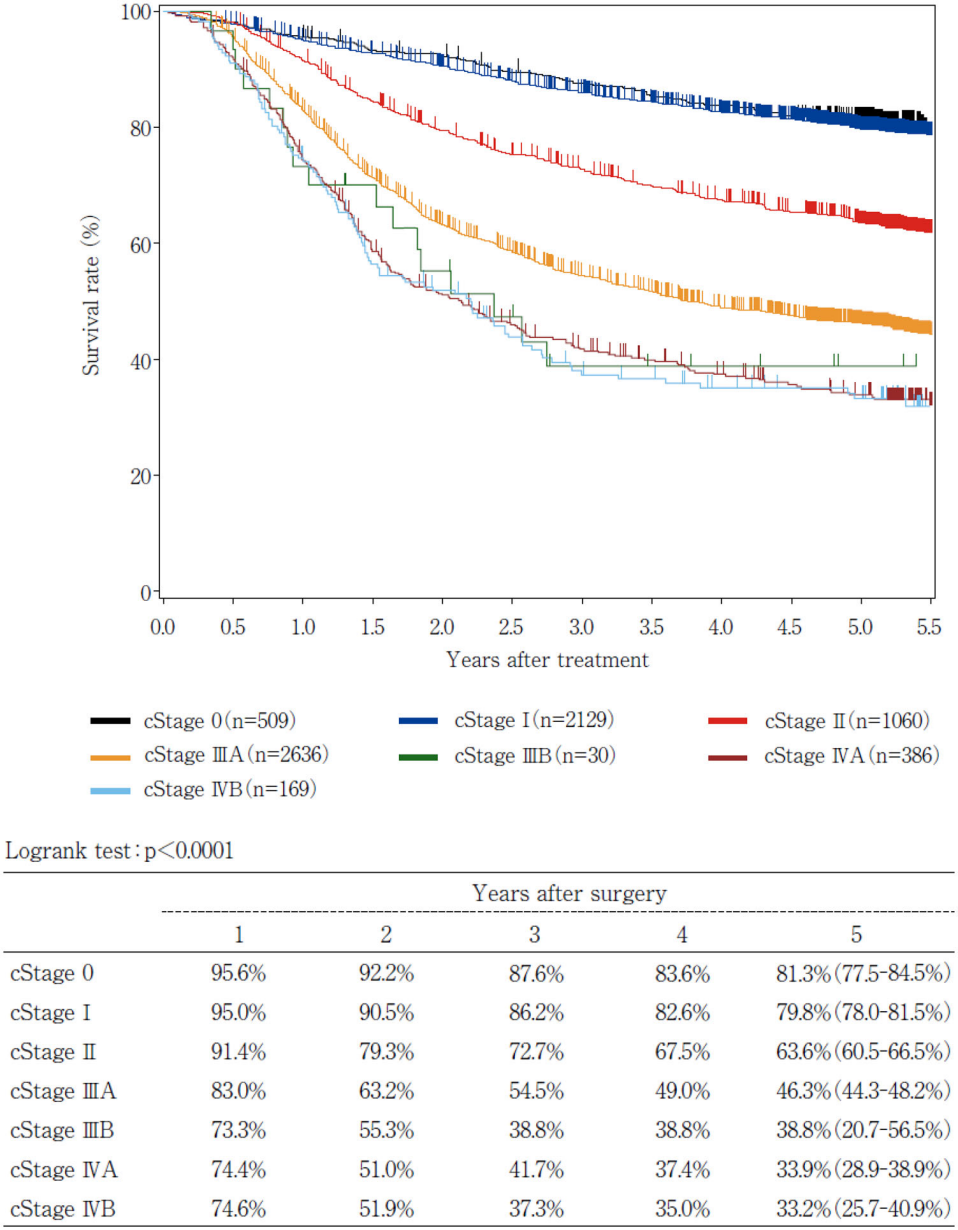
Survival curve according to clinical stage based on this 12th classification. Survival curves for surgical cases from the National Esophageal Cancer Registry 2010-2012 are shown. Figures in parentheses mean 95% confidence intervals for estimated 5-year survival rates. (Stage I: All patients; Stage II–IV: Preoperatively treated patients)

**Fig. 70:**
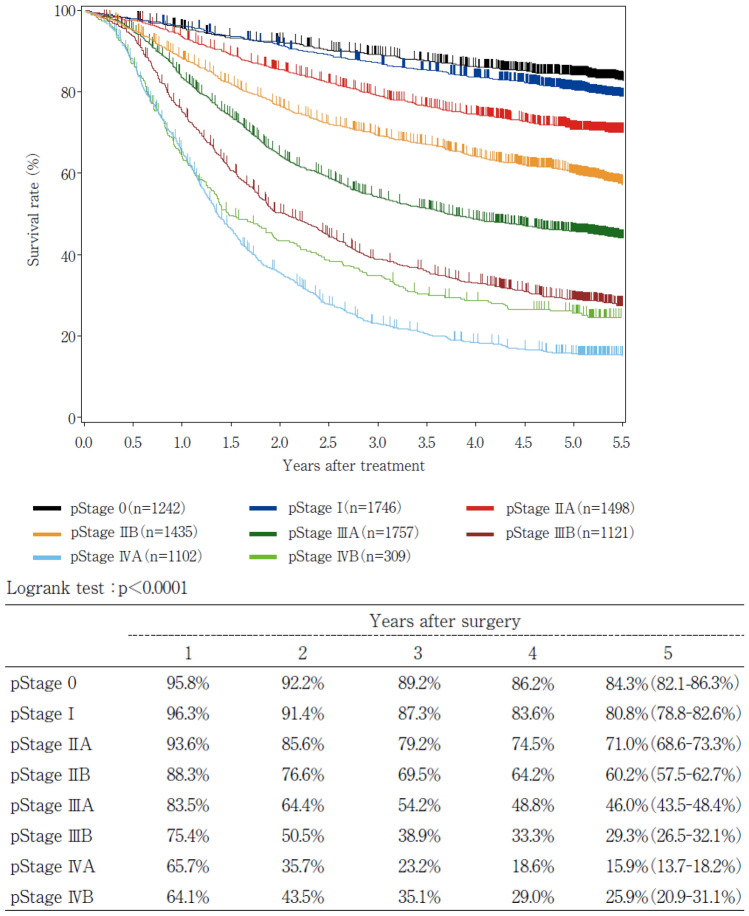
Survival curve according to pathological stage based on this 12th classification. Prognostic curves for surgical cases (including both untreated and preoperatively treated cases) from the National Esophageal Cancer Registry 2010-2012 are shown. Figures in parentheses mean 95% confidence intervals for estimated 5-year survival rates

See Figs. 69 and 70.

## Data Availability

Data sharing is not applicable to this article as no datasets were generated or analyzed during the current study.

## Abbreviations

A: Anterior
AD: Adventitia
AI: Invasion to the adjacent organs
APC: Argon plasma coagulation
BA: Brownish area
BLI: Blue laser imaging
Br: Borderline resectable
c: Clinical findings
Ce: Cervical esophagus
CR: Complete response
CRT: Chemoradiotherapy
CT: Chemotherapy
CTV: Clinical target volume
D: Extent of lymph-node dissection
DM: Distal margin
DMM: Deep muscularis mucosae
E: Esophagus
EGJ: Esophagogastric junction
EI: Esophageal invasion
EMR: Endoscopic mucosal resection
EP: Epithelium
ER: Endoscopic resection
ESD: Endoscopic submucosal dissection
f: Final findings
G: Stomach
GI: Gastric invasion
GIST: Gastrointestinal stromal tumor
HH: Hiatus hernia
HM: Horizontal margin
HT: Hyperthermia
IM: Intramural metastasis
INF: Infiltrative growth pattern
IO: Immuno-oncology drug
Jz: Zone of esophagogastric junction
Laser: Laser therapy
LPM: Lamina propria mucosae
LR: Local recurrence
LSBE: Long-segment Barrett’s esophagus
Lt: Lower thoracic esophagus
Ly: Lymphatic invasion
Ly/V: Lymphatic invasion or venous invasion
M: Distant organ metastasis
MCT: Microwave coagulation therapy
MM: Muscularis mucosae
MP: Muscularis propria
Mt: Middle thoracic esophagus
N: Grading of lymph-node metastasis
NBI: Narrow band imaging
p: Pathological findings
P: Posterior
PD: Progressive disease
PDT: Photodynamic therapy
Ph: Pharynx
PM: Proximal margin
PR: Partial response
R: Residual tumor
RECIST: Response evaluation criteria in solid tumors
RM: Radial margin
RR: Remarkable response
RT: Radiotherapy
SCE: Specialized columnar epithelium
SCJ: Squamocolumnar junction
SD: Stable disease
SIN: Squamous intraepithelial neoplasia
SM: Submucosal layer
SMM: Superficial muscularis mucosae
SSBE: Short-segment Barrett’s esophagus
T: Depth of tumor invasion
Te: Thoracic esophagus
Tis: Carcinoma in situ
Ut: Upper thoracic esophagus
V: Venous invasion
VM: Vertical margin
X: Cannot be assessed

## Terminology of the lymph nodes

R: Right
L: Left
sm: Submandibular
spf: Superficial
ac: Accessory
tr: Tracheal
up: Upper
mid: Middle
rec: Recurrent nerve
tb: Tracheobronchial
pre: Pretracheal
ao: Para-aortic
pul: Pulmonary ligament
